# GPR15-guided CD8^+^ T regulatory cells control intestinal inflammation

**DOI:** 10.1038/s41586-026-10749-4

**Published:** 2026-06-08

**Authors:** Jing Cui, Zuojia Chen, Yan H. Cheng, Jia Nie, Maria H. Zhu, Chen Xiang, Samuel Chauvin, Wiem Lassoued, Can Liu, Kartika Padhan, Jian Song, Joy A. Pai, Borja Ocón, Yifan Yang, Yikun Yao, Ann Y. Park, Justin Q. Gabrielski, Lixin Zheng, Ping Du Jiang, Yu Zhang, Huie Jing, Leilei Xu, Vicky Chen, Brent A. Calabresi, Stefania Pittaluga, David E. Kleiner, Clifton L. Dalgard, Sinan Sari, Salim Can, Elif Karakoc-Aydiner, Safa Baris, Baran Erman, Michael Field, Jocelyn A. Silvester, Gwen Saccocia, Eman K. Buhamrah, Khaled Husain, Zahra Chavoshzadeh, Nima Rezaei, Ali İşlek, Hasan Baş, Fatma Dereli Devrez, Rafah Mackeh, Ivan J. Fuss, Daniel S. Reich, Shania Bailey, Houssein Abdul Sater, James L. Gulley, Juraj Kabat, Margery Smelkinson, Sundar Ganesan, Zhenghao Chen, Peter C. Grayson, Neelam Redekar, Justin Lack, Zhiyong Lu, Bernice Lo, Hassan Abolhassani, Ahmet Ozen, Athos Bousvaros, Scott B. Snapper, Eugene C. Butcher, Juan Ravell, Rémy Bosselut, Helen C. Su, Chuan Wu, Michael J. Lenardo

**Affiliations:** 1 Molecular Development of the Immune System Section, Laboratory of Immune System Biology, National Institute of Allergy and Infectious Diseases (NIAID), National Institutes of Health (NIH), Bethesda, MD USA; 2 Clinical Genomics Program, NIAID, NIH, Bethesda, MD USA; 3 Calico Life Sciences, South San Francisco, CA USA; 4 Experimental Immunology Branch, National Cancer Institute (NCI), NIH, Bethesda, MD USA; 5 Laboratory of Immune Cell Biology and Laboratory of Integrative Cancer Immunology, Center for Cancer Research, NCI, NIH, Bethesda, MD USA; 6 Perelman School of Medicine, University of Pennsylvania, Philadelphia, PA USA; 7 Center for Immuno-Oncology, Center for Cancer Research, NCI, NIH, Bethesda, MD USA; 8 Multiscale Systems Biology Section, Laboratory of Immune System Biology, NIAID, NIH, Bethesda, MD USA; 9 Program in Biological Sciences, University of Maryland, College Park, MD USA; 10 Laboratory of Immune System Biology, NIAID, NIH, Bethesda, MD USA; 11 Laboratory of Immunology and Vascular Biology, Department of Pathology, School of Medicine, Stanford University, Stanford, CA USA; 12 Palo Alto Veterans Institute for Research, Palo Alto, CA USA; 13 Veterans Affairs Palo Alto Health Care System, Palo Alto, CA USA; 14 Division of Intramural Research, National Library of Medicine, NIH, Bethesda, MD USA; 15 Laboratory of Clinical Immunology and Microbiology, NIAID, NIH, Bethesda, MD USA; 16 Integrated Data Sciences Section, Research Technologies Branch, NIAID, NIH, Bethesda, MD USA; 17 Translational Neuroradiology Section, National Institute of Neurological Disorders and Stroke, NIH, Bethesda, MD USA; 18 Laboratory of Pathology, Center for Cancer Research, NCI, NIH, Bethesda, MD USA; 19 Department of Anatomy, Physiology and Genetics, Uniformed Services University, Bethesda, MD USA; 20 Center for Military Precision Health, The American Genome Center, Uniformed Services University, Bethesda, MD USA; 21 Department of Pediatric Gastroenterology, Gazi University Faculty of Medicine, Ankara, Türkiye; 22 Department of Pediatrics, Division of Allergy and Immunology, Marmara University, School of Medicine, Istanbul, Türkiye; 23 The Istanbul Jeffrey Modell Diagnostic Center for Primary Immunodeficiency Diseases, Istanbul, Türkiye; 24 The Isil Berat Barlan Center for Translational Medicine, Istanbul, Türkiye; 25 Institute of Child Health, Can Sucak Research Laboratory for Translational Immunology, Hacettepe University, Ankara, Türkiye; 26 Division of Gastroenterology, Hepatology and Nutrition, Department of Pediatrics, Boston Children’s Hospital, Boston, MA USA; 27 Pediatric Gastroenterology and Hepatology Department, Al-Amiri Hospital, Kuwait City, Kuwait; 28 Pediatric Gastroenterology, Hepatology and Nutrition Department, Dar Al Shifa Hospital, Kuwait City, Kuwait; 29 Allergy and Clinical Immunology Department, Mofid Children Hospital, Shahid Beheshti University of Medical Sciences, Tehran, Iran; 30 Research Center for Immunodeficiencies, Pediatrics Center of Excellence, Children’s Medical Center, Tehran University of Medical Sciences, Tehran, Iran; 31 Cukurova University Faculty of Medicine, Department of Pediatrics, Division of Gastroenterology and Hepatology, Adana, Türkiye; 32 Intergen Genetics and Rare Diseases Diagnosis Center, Ankara, Türkiye; 33 Translational Medicine Department, Research Department, Sidra Medicine, Doha, Qatar; 34 Mucosal Immunity Section, Laboratory of Clinical Immunology and Microbiology, NIAID, NIH, Bethesda, MD USA; 35 Biological Imaging Section, Research Technologies Branch, NIAID, NIH, Bethesda, MD USA; 36 Vasculitis Translational Research Program, National Institute of Arthritis and Musculoskeletal, and Skin Diseases, NIH, Bethesda, MD USA; 37 College of Health and Life Sciences, Hamad Bin Khalifa University, Doha, Qatar; 38 Division of Immunology, Department of Medical Biochemistry and Biophysics, Karolinska Institute, Stockholm, Sweden; 39 Clinic for Pediatric Immunology and Rheumatology, Center for Pediatrics and Adolescent Medicine, University Hospital Bonn, Bonn, Germany; 40 Division of Allergy and Immunology, Department of Internal Medicine, Hackensack University Medical Center, Hackensack, NJ USA; 41 Department of Internal Medicine, Hackensack Meridian School of Medicine, Nutley, NJ USA

**Keywords:** Inflammatory bowel disease, Autoimmunity

## Abstract

Inflammatory bowel disease (IBD) causes chronic suffering from gastrointestinal inflammation and dysfunction that can progress to colon cancer^1,2^. The prevalence of the disease is increasing, and there is an urgent need to better understand its pathogenic mechanisms to improve treatment. We show that GPR15—a G-protein-coupled receptor expressed in immune cells and described previously as an entry co-factor for human and simian immunodeficiency viruses^3^—is a marker and homing receptor for a subset of intramucosal GPR15-guided regulatory CD8^+^ T lymphocytes (CD8^+^ T_IGR_ cells). Deleterious GPR15 gene variants in humans cause defective homing of CD8^+^ T_IGR_ cells and are associated with severe early-onset IBD. Moreover, CD8^+^ T_IGR_ cells are reduced in the intestinal mucosa of individuals with sporadic IBD. In mice, GPR15 deficiency impairs colonic homing of CD8^+^ T_IGR_ cells, leading to accumulation of inflammatory macrophages and increased susceptibility to colitis. CD8^+^ T_IGR_ cells potently kill macrophages activated by intestinal damage or disease using Fas ligand and TNF-related weak inducer of apoptosis (TWEAK). The identification of CD8^+^ T_IGR_ cells yields new insights into organ-specific immune regulation and potential therapeutics for IBD.

## Abstract (web summary)

Intramucosal GPR15-guided regulatory CD8^+^ T lymphocytes—which are diminished by deleterious *GPR15* genetic mutations in severe, early-onset disease or by cell deficiencies in sporadic ulcerative colitis and Crohn’s disease—restrain macrophage-driven inflammation and present new potential therapeutic avenues for inflammatory bowel disease.

## Main

Inflammatory bowel disease (IBD), including ulcerative colitis and Crohn’s disease, is influenced by genetic and environmental factors^4,5^. Variants in more than 90 genes have been associated with early-onset IBD, illuminating key pathogenic mechanisms^6,7^. Identifying additional IBD-causing genes could clarify pathological mechanisms and lead to better diagnosis and therapies. G-protein-coupled receptor 15 (GPR15) was originally identified in vitro as a retroviral coreceptor^3^. Later studies in mice suggested that it mediates tissue-specific homing of CD4^+^FOXP3^+^ regulatory T (T_reg_) cells^8^ or CD4^+^ effector T (T_eff_) cells^9^. GPR15-deficient mice have worsened colitis and a susceptibility to colon cancer^8,10^. A CC motif-containing agonistic chemokine ligand for GPR15 (GPR15L) that is found in mouse and human colonic epithelia guides migration of GPR15^+^ lymphocytes^11–13^. GPR15 expressed on CD4^+^ T cells and upregulated on T_reg_ cells in individuals with ulcerative colitis may influence IBD^14,15^. How GPR15 specifically regulates human intestinal immune homeostasis or IBD is unknown.

A subset of human CD8^+^ T cells expressing inhibitory killer cell immunoglobulin-like receptors (KIRs) can selectively suppress pathogenic CD4^+^ T cells in autoimmune or infectious diseases^16^. In addition, CD8^+^ T cells among intestinal intra-epithelial lymphocytes (IELs) can express NKG2D and NKG2A and function in immune defence and regulation^17,18^. Human IELs comprise three subsets: T cell receptor (TCR)αβ^+^CD8αβ^+^, TCRαβ^+^CD4^+^ and TCRγδ^+^ cells. Mouse IELs have the same subsets plus an additional TCRαβ^+^CD8αα^+^ subset that partly expresses LY49 and ameliorates T cell-induced colitis^17,19,20^. However, whether the equivalent TCRαβ^+^CD8αα^+^ IEL subset exists in humans and its putative function remain unclear^21^.

## GPR15 variants in early-onset IBD

**Fig. 1:**
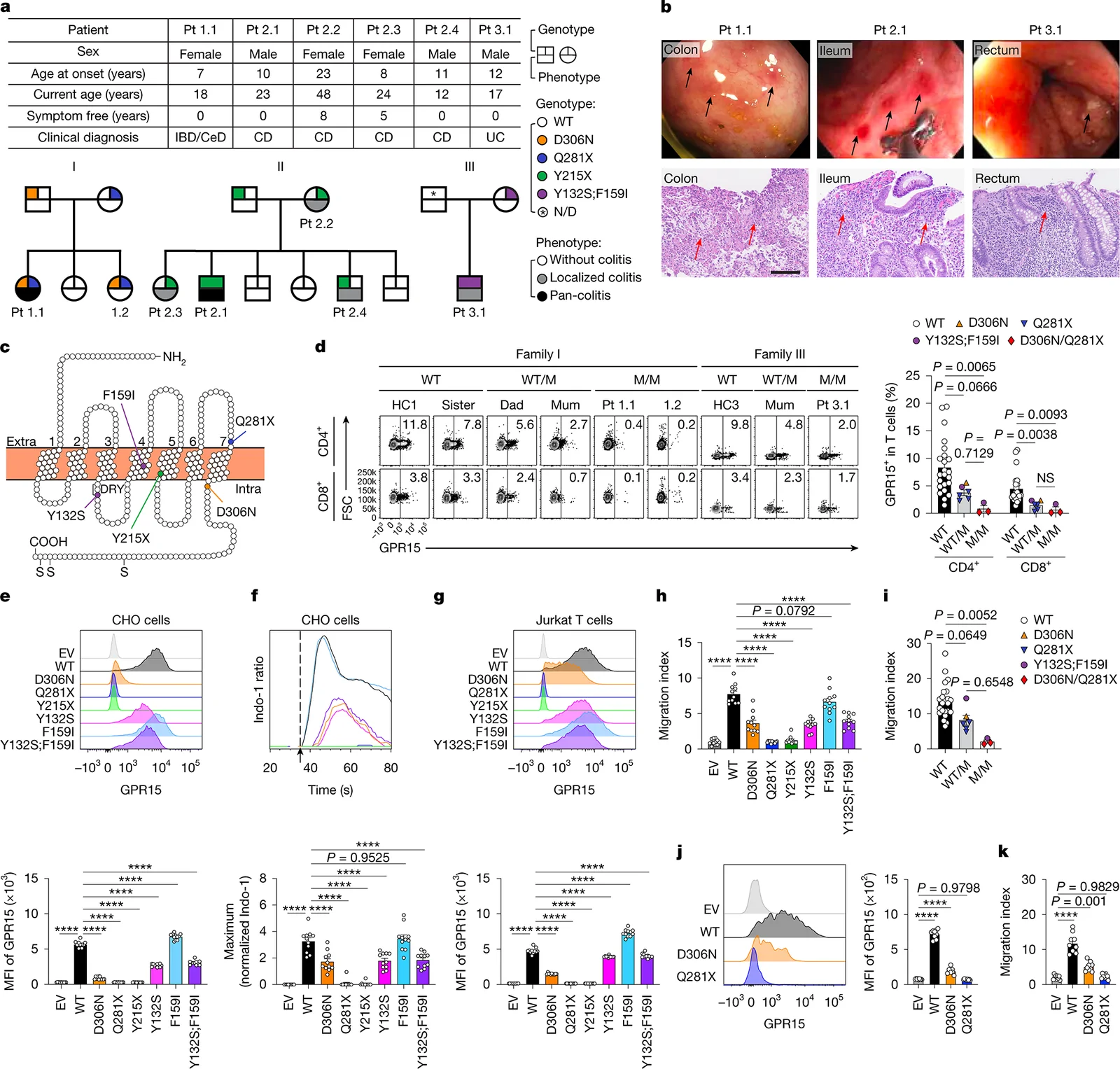
*GPR15* variants in patients with early-onset IBD. **a**, Top, patient clinical and demographic features; bottom, pedigrees of three families with IBD. **b**, Top, lower gastrointestinal endoscopy; bottom, haematoxylin and eosin (H&E)-stained biopsy sections. Black arrows, ulcers; red arrows, inflammatory infiltrates. Images are representative of two independent examinations in three patients during active IBD, with one inflamed segment shown per patient and two biopsy fragments per segment. Adjacent-section H&E staining confirmed reproducibility. **c**, GPR15 in membrane (orange) with mutant residues, extracellular (Extra) and intracellular (Intra) regions, DRY motif and C-terminal serine (S) residues. **d**, Left, GPR15 surface expression in peripheral CD4^+^ and CD8^+^ T cells from healthy individuals (HC1 and HC3) and participants from families I and III. Right, GPR15^+^ cell fraction in blood CD4^+^ and CD8^+^ T cells from families I, III and IV; WT, *n *= 23; monoallelic mutations (WT/M), *n *= 6; biallelic mutations (M/M), *n *= 3. **e**, Top, GPR15 surface expression in CHO-K1/G_α16_ cells transduced with WT or mutant gene or empty vector (EV); bottom, quantification of mean fluorescence intensity (MFI). **f**, Top, Ca^2+^ flux (Indo-1) in cells in **e** stimulated with GPR15L. Bottom, fold change of maximum ratio. **g**, As in **e** but for Jurkat T cells transduced with WT or mutant genes or EV. **h**, Migration towards GPR15L in Jurkat T cells transduced as in **g**. **i**, T cell migration towards GPR15L from **d** after anti-CD3/CD28 stimulation (9 days). **j**,**k**, GPR15 surface expression (**j**) and migration towards GPR15L (**k**) of T cells from Pt 1.1 transduced with EV or WT, D306N or Q281X genes. Data are representative (**b**; **d**,**j**, left; **e**–**g**, top) or mean ± s.e.m. pooled from three independent experiments (**e**–**h**; **j**, right; **k**) or 3–23 people (**d**,**i**); *n *= 11 per group (**e**–**h**); *n *= 9 per group (**j**,**k**). Statistics, one-way analysis of variance (ANOVA) with Dunnett’s test (**e**–**h**,**j**,**k**) or Kruskal–Wallis with Dunn’s test (**d**,**i**). NS, not significant, *P *> 0.9999; ****P < 0.0001. Scale bar, 100 μm. CD, Crohn’s disease; CeD, Coeliac disease; UC, ulcerative colitis. Source data

**Extended Data Fig. 1:**
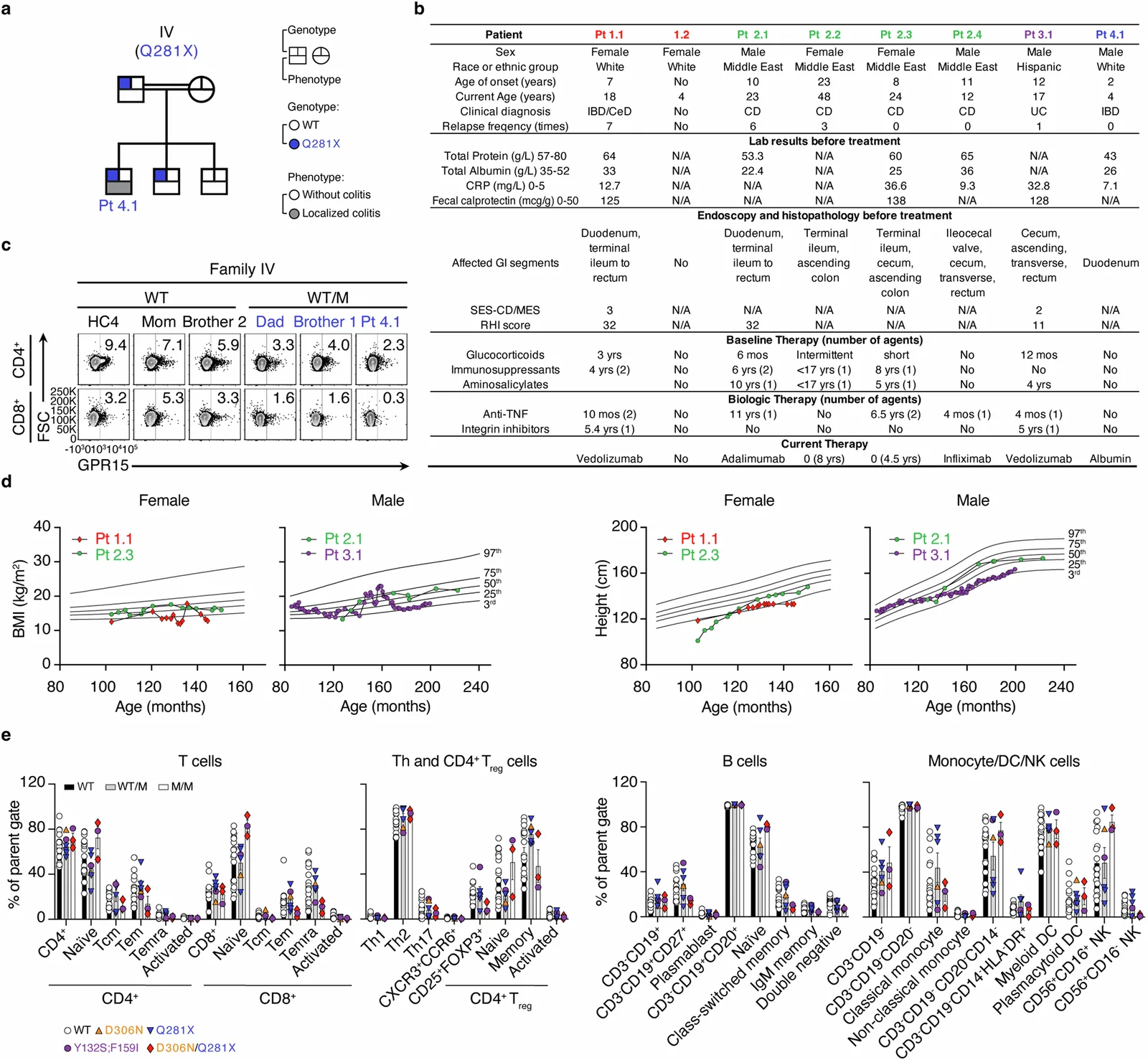
Patients with GPR15 mutations have early-onset IBD with normal peripheral immunophenotypes. **a**, Family IV pedigree showing genotypes (symbol top half) and phenotypes (symbol bottom half). Double line, consanguinity. Bottom: white, without colitis; grey, localized colitis. **b**, Extended table of clinical and demographic features of all patients from Families I-IV. SES-CD, simple endoscopic score for Crohn’s disease; MES, Mayo endoscopic subscore; RHI score, Robarts histopathology index for IBD; CRP, C-reactive protein; GI, gastrointestinal; yrs, years; mos, months; N/A, not applicable. **c**, Representative flow cytometry of GPR15 surface expression in peripheral CD4^+^ and CD8^+^ T cells from healthy controls (HC), Pt 4.1 and family members in Family IV. Wild-type, black; monoallelic mutation, blue. **d**, Growth curves of body mass index (BMI, left) and height/length (right) as a function of age (months) for Pt 1.1, Pt 2.1, Pt 2.3 and Pt 3.1. Percentile curves for age-matched controls in the general population are shown. **e**, Flow cytometry immunophenotyping of peripheral blood from HC, patients and family members in Families I, III and IV. WT, *n* = 18; Monoallelic mutations (WT/M), *n* = 6; Biallelic mutations (M/M), *n* = 3. Subsets: naïve (CCR7^+^CD45RA^+^), central memory (T_CM_) (CCR7^+^CD45RA^−^), effector memory (T_EM_) (CCR7^−^CD45RA^−^), activated (CD38^+^HLA-DR^+^) and effector memory re-expressing CD45RA (T_EMRA_) (CCR7^−^CD45RA^+^) CD4^+^ or CD8^+^ T cells, CD4^+^ T helper (Th) or T regulatory (T_reg_) cell subsets, B cell subsets, monocyte, dendritic cell (DC) and natural killer (NK) cell subsets. Data are representative (**c**) or mean ± s.e.m. (**e**) pooled from 3-18 people. Statistical significance was determined by Kruskal–Wallis with Dunn’s multiple-comparisons test (**e**), *P* > 0.05. Source data

We investigated seven individuals from four unrelated families who had been diagnosed with IBD, presenting with episodic diarrhoea, weight loss, abdominal pain, gastrointestinal upset and increased inflammatory markers (Fig. 1a, Extended Data Fig. 1a–d and Supplementary Table 1; clinical summaries are in the Supplementary Information). Symptoms began in early childhood, accompanied by failure to thrive and subnormal height and weight, except in the mother of patient (Pt) 2.1 (Pt 2.2) who developed symptoms at age 23 years (Fig. 1a and Extended Data Fig. 1b,d). Lower gastrointestinal endoscopy showed nodules, ulcers, hyperaemia, inflammation and cobblestone patterning, and histopathology showed lymphocytic infiltration and disrupted villus architecture (Fig. 1b). Pt 1.1 and Pt 2.1 exhibited severe pan-colitis from the terminal ileum to the rectum, whereas the other patients had lesions localized to specific gastrointestinal segments (Fig. 1a and Extended Data Fig. 1a,b); Pt 1.1 also had clinicopathological and genetic indices of coeliac disease (Fig. 1a and clinical summaries). Haematological parameters, immune cells and immunoglobulin profiles were largely normal, with increased TCRαβ^+^CD4^−^CD8^−^ ‘double negative’ cells in Pt 1.1 and Pt 3.1 and occasional eosinophil elevations in Pt 1.1 and Pt 2.1 (Extended Data Fig. 1e and Supplementary Table 1). Thus, disease was confined largely to the gastrointestinal tract without consistent systemic immune abnormalities.

**Extended Data Fig. 2:**
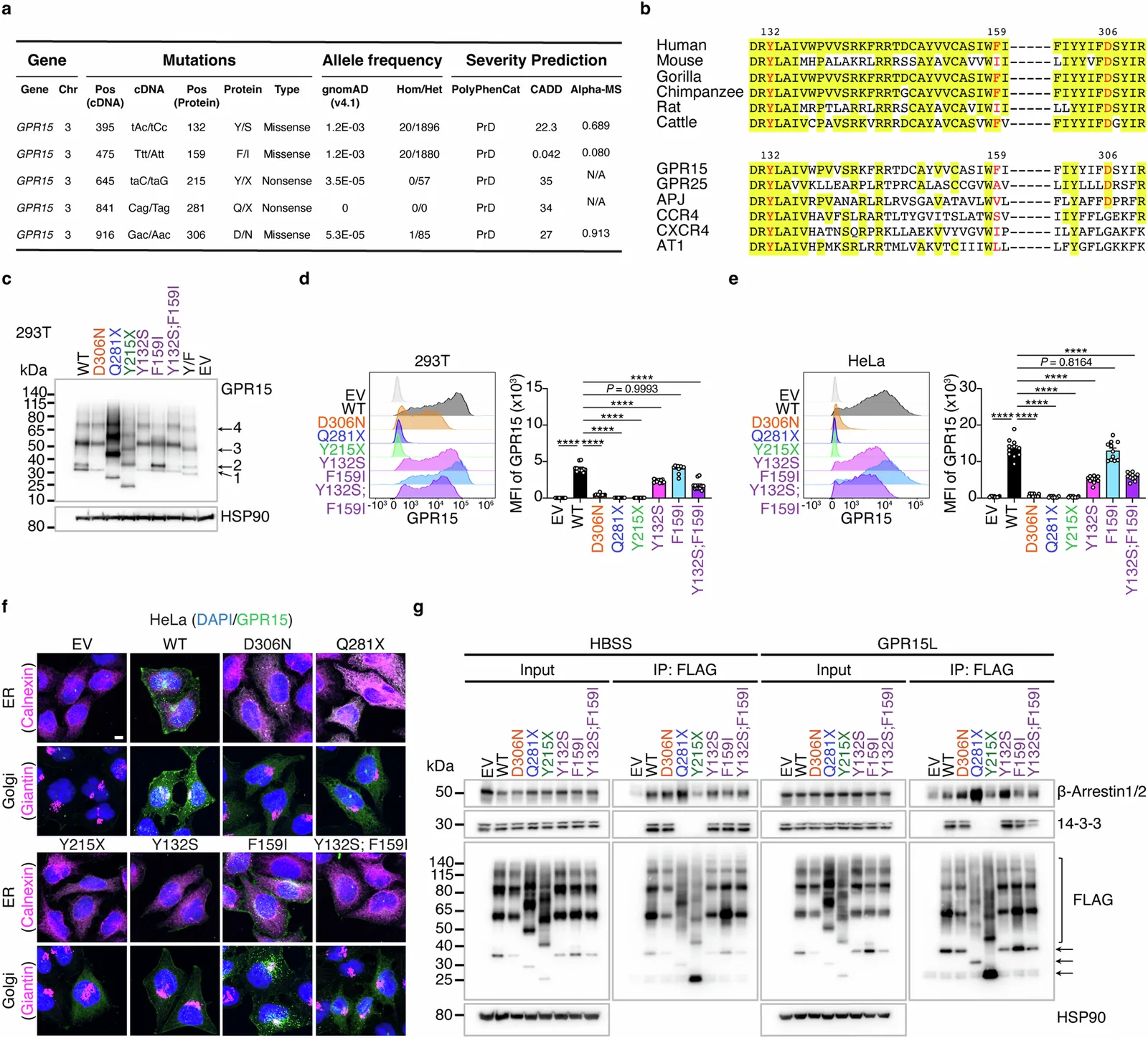
GPR15 mutations cause reduced surface expression. **a**, Bioinformatic information for GPR15 variants in four families. Chr, chromosome; Pos, position; Hom, homozygotes; Het, heterozygotes. Nucleotide changes in caps; amino acid in single-letter code (X, stop codon). Alpha-MS, AlphaMissense. **b**, Amino acid alignment illustrating conservation in GPR15 orthologs (upper) and paralogues (lower); patient variants highlighted in red. **c**, Immunoblots of GPR15 (upper) and HSP90 (loading control, lower) in HEK 293T cells transfected with constructs expressing WT, patient mutations, Y/F mutant (GPR15L binding-deficient) or empty vector (EV). Band 1, non-glycosylated protein; bands 2, 3, presumably glycosylated forms; band 4, dimers or oligomers. **d, e**, Flow cytometry of GPR15 surface expression in HEK 293T (**d**) and HeLa (**e**) cells transduced with WT, patient mutants or EV. Right, mean fluorescence intensity (MFI) quantified. *n* = 11 per group. **f**, Confocal imaging of HeLa cells as in **e** stained for GPR15, calnexin (ER marker), giantin (Golgi marker) and DAPI. Scale bars, 5 μm. **g**, Immunoblots of total lysates (Input) or immunoprecipitates (IP) pulled down with rat anti-FLAG antibody from HEK 293T cells expressing FLAG-tagged GPR15 variants or EV, probed for β-arrestin-1/2, 14-3-3, FLAG or HSP90 (loading control). Cells were stimulated with vehicle (HBSS, left) or 100 nM GPR15 ligand (GPR15L, right) for 30 min. Arrows, non-glycosylated proteins; bracket, presumably glycosylated, dimers or oligomeric forms. Data show representative (**c**; **d**, **e**, left; **f**, **g**) or mean ± s.e.m. (**d**, **e**, right) pooled from 3 independent experiments. KDa = relative migration in kilodaltons (**c**, **g**). Statistical significance was determined by one-way (ANOVA) with Dunnett’s multiple-comparisons test **(d**, **e**, right). ****P < 0.0001. Source data

Because of early onset and family history, we performed whole-exome sequencing (WES) to investigate the possibility of monogenic disease. Considering rare protein-altering variants with a minor allele frequency (MAF) of around less than 0.1% (from dbSNP, NHLBI 1000 Genomes, gnomAD and Yale exome databases; homozygous, compound heterozygous or heterozygous variants with potential dominant or haploinsufficient effects), we found that all families shared potentially deleterious variants in the *GPR15* gene (Fig. 1a,c, Extended Data Figs. 1a and 2a and Supplementary Fig. 1a). Pt 1.1 was compound heterozygous for p.D306N and p.Q281X. Sibling 1.2, the 4-year-old sister of Pt 1.1, carried the same compound heterozygous mutations but was not symptomatic. Pt 2.1 was homozygous for p.Y215X. Pt 3.1 was doubly homozygous for the p.Y132S and p.F159I missense variants (the latter predicted to be benign). The Y132S variant is present in gnomAD databases at a non-negligible frequency (MAF = 0.0012, 1,916 heterozygotes and 20 homozygotes in v.4.1.1), including homozygous people, which is inconsistent with a fully penetrant monogenic disease and suggests a hypomorphic allele with incomplete penetrance. Mild or episodic IBD symptoms were present in monoallelic carriers of p.Q281X (Pt 4.1) and p.Y215X (Pt 2.2, Pt 2.3 and Pt 2.4), whereas biallelic carriers exhibited more severe IBD symptoms, supporting a gene dosage effect. Overall, penetrance was partial in both biallelic (three of four; 75%) and monoallelic (four of ten; 40%) heterozygous carriers. The altered residues occurred throughout the protein and caused amino acid substitutions or stop codons within or near the transmembrane domains (Fig. 1c and Extended Data Fig. 2b). Moreover, residues Y132 and D306 are conserved evolutionarily, whereas F159 is isoleucine in rodents (Extended Data Fig. 2b).

## Patient *GPR15* mutations impair function

We found that GPR15 surface expression in peripheral blood mononuclear cells (PBMC) was reduced in biallelic carriers (mutant (M)/M) and intermediate in monoallelic carriers (wild type (WT)/M) compared with healthy individuals with WT GPR15 (Fig. 1d and Extended Data Fig. 1c). Biochemically, immunoblot analysis of GPR15-overexpressing 293T cells revealed four principal WT GPR15 bands (70 kDa, 55 kDa, 36 kDa and 32 kDa), with the 32-kDa band corresponding to the full-length protein and larger species reflecting oligomers or post-translational modifications^22^ (Extended Data Fig. 2c). Missense variants (D306N, Y132S and Y132S/F159I) reduced or abolished the 32-kDa and 36-kDa species, whereas stop-gain variants (Q281X and Y215X) generated shorter main species, the smallest being 27 kDa and 20 kDa, respectively. F159I alone increased the 36-kDa species (Extended Data Fig. 2c) slightly. GPR15 surface expression was reduced for all variants except F159I and was absent for truncating mutants (Extended Data Fig. 2d).

Like most G-protein-coupled receptors (GPCRs), GPR15 localizes on the plasma membrane, where it mediates chemotaxis in response to ligands such as GPR15L^11,12^. In HeLa cells, WT and F159I GPR15 localized to the Golgi and cell surface, whereas Y132S and Y132S/F159I GPR15 expression was reduced. By contrast, the D306N and Q281X variants were largely trapped in the endoplasmic reticulum, and Y215X GPR15 showed little expression (Extended Data Fig. 2e,f). These results indicate that GPR15 mutations impair its intracellular trafficking to the cell surface. It is known that 14-3-3 and β-arrestin regulate GPR15 surface expression and internalization^23,24^. Immunoprecipitation showed that 14-3-3 bound to WT and missense variants but not truncating variants (Q281X, Y215X), which lack cytoplasmic tails (Extended Data Fig. 2g, left). GPR15L stimulation had minimal effect on 14-3-3 binding, except for reduced association with the Y132S/F159I variant (Extended Data Fig. 2g, right). By contrast, β-arrestin binding differed: missense variants resembled WT, whereas Q281X GPR15 showed increased binding and Y215X GPR15 showed decreased binding (Extended Data Fig. 2g, left), indicating structural disruption by truncating mutants. GPR15L increased β-arrestin binding to most variants except F159I (Extended Data Fig. 2g, right).

We assessed GPR15L-stimulated Ca^2+^ influx assessed in CHO-K1 cells expressing WT GPR15 or patient variants. WT and F159I GPR15 showed the highest surface expression, whereas the D306N, Y132S and Y132S/F159I variants were reduced and Q281X and Y215X were absent (Fig. 1e). GPR15L stimulation induced Ca^2+^ influx in proportion to GPR15 surface expression, except for the F159I variant, indicating a quantitative signalling defect (Fig. 1f). Nonetheless, F159I GPR15 was unable to rescue the poor calcium response of the Y132S variant, indicating a dominant effect by Y132S (Fig. 1f). In Jurkat T cells lacking endogenous GPR15, variants showed the same expression defects as in CHO-K1 cells (Fig. 1g). In transwell assays, cells expressing WT and F159I GPR15 showed robust GPR15L-induced migration, whereas cells expressing the other variants were deficient in migration (Fig. 1h).

**Extended Data Fig. 3:**
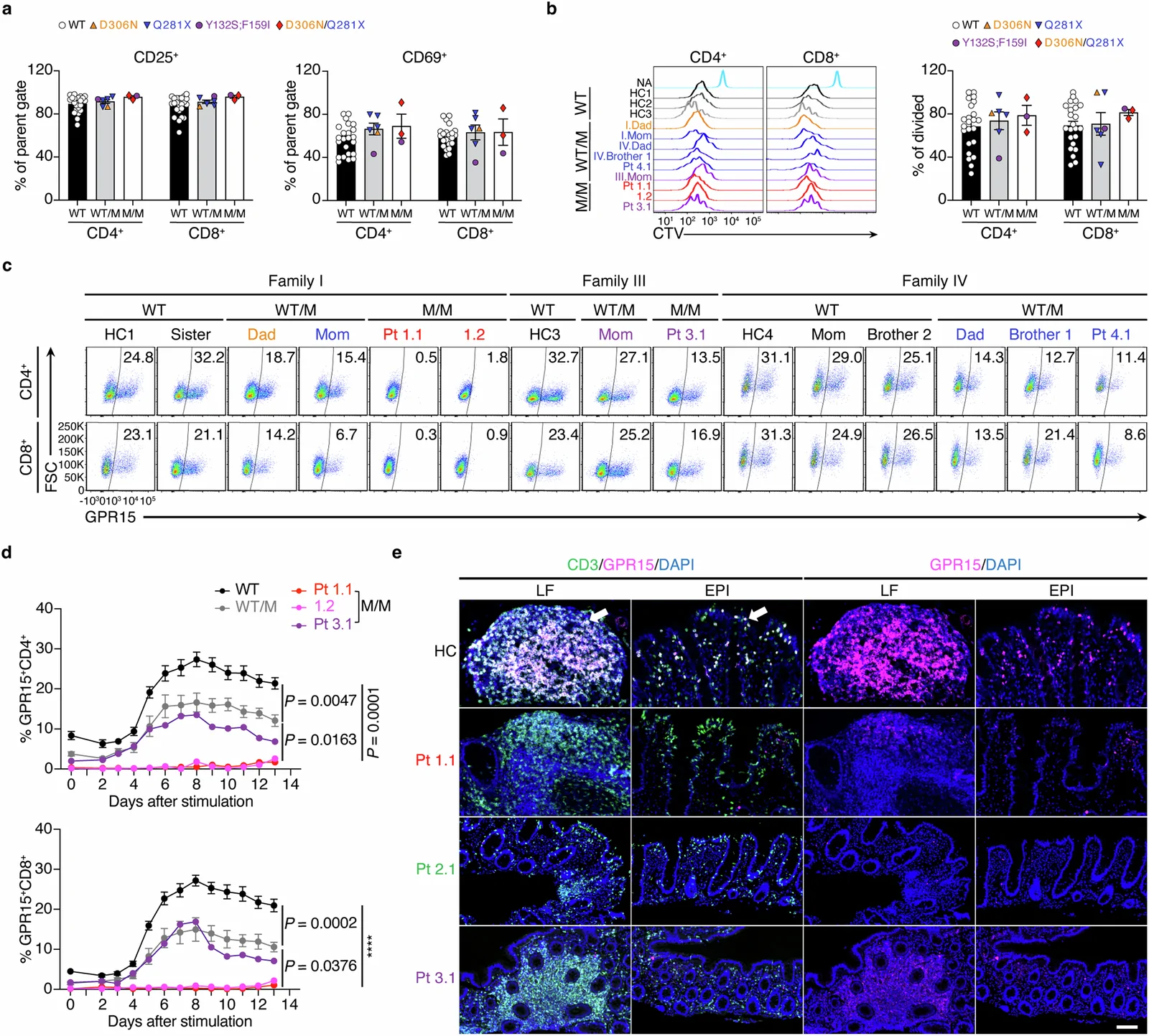
Defective GPR15 expression in patient T cells. **a**, Percentage of CD25^+^ (left) or CD69^+^ (right) cells in CD4^+^ or CD8^+^ T cells from HCs, patients and family members of Families I, III and IV after activation with anti-CD3/CD28 (1 μg/mL each, 2 days). WT, *n* = 23; WT/M, *n* = 6; M/M, *n* = 3. **b**, Flow cytometry of T cell proliferation as in **a**, measured by CellTrace Violet (CTV) dilution 3 days after activation. WT, *n* = 23; WT/M, *n* = 6; M/M, *n* = 3. NA, Non-activated. Right, quantification of divided cells (%). **c**, Flow cytometry of GPR15 surface expression in CD4^+^ (upper) and CD8^+^ (lower) T cells as in **a**, 8 days after activation. Values indicate the fraction of GPR15^+^ cells. FSC, forward scatter. **d**, Fractions of GPR15^+^ cells in CD4^+^ (upper) or CD8^+^ T cells (lower) in **c** after anti-CD3/CD28 stimulation. WT, *n* = 23; WT/M, *n* = 6; M/M, *n* = 3. **e**, Representative immunofluorescence images stained for CD3, GPR15 and DAPI in colonic biopsies from HC, Pt 1.1, Pt 2.1 and Pt 3.1. LF, lymphoid follicles; EPI, epithelium. Scale bars, 100 μm. Data show representative (**b**, left, **c**, **e**) or mean ± s.e.m. pooled from 3-23 people (**a**; **b**, right; **d**). Statistical significance was determined by Kruskal–Wallis with Dunn’s multiple-comparisons test (**a**; **b**, right) or two-way ANOVA tests (**d**). ****P < 0.0001. Source data

T cell activation and proliferation were normal in M/M and WT/M PBMCs (Extended Data Fig. 3a,b). During in vitro activation, GPR15 expression increased in CD4^+^ and CD8^+^ T cells but was attenuated in WT/M and greatly reduced in M/M cells (Extended Data Fig. 3c,d). Furthermore, migration towards GPR15L was reduced in WT/M T cells and further impaired in M/M T cells (Fig. 1i). Lentiviral reconstitution of WT GPR15 in T cells from Pt 1.1 restored surface expression and migration, whereas D306N and Q281X GPR15 showed minimal or no rescue (Fig. 1j,k). In intestinal biopsies, most GPR15^+^ cells from control individuals were CD3^+^ T cells, which were nearly absent in Pt 1.1, Pt 2.1 and Pt 3.1 (Extended Data Fig. 3e). Thus, patient mutations impaired GPR15 surface expression and function in vitro and in vivo.

## Deleterious *GPR15* alleles linked to IBD

**Extended Data Fig. 4:**
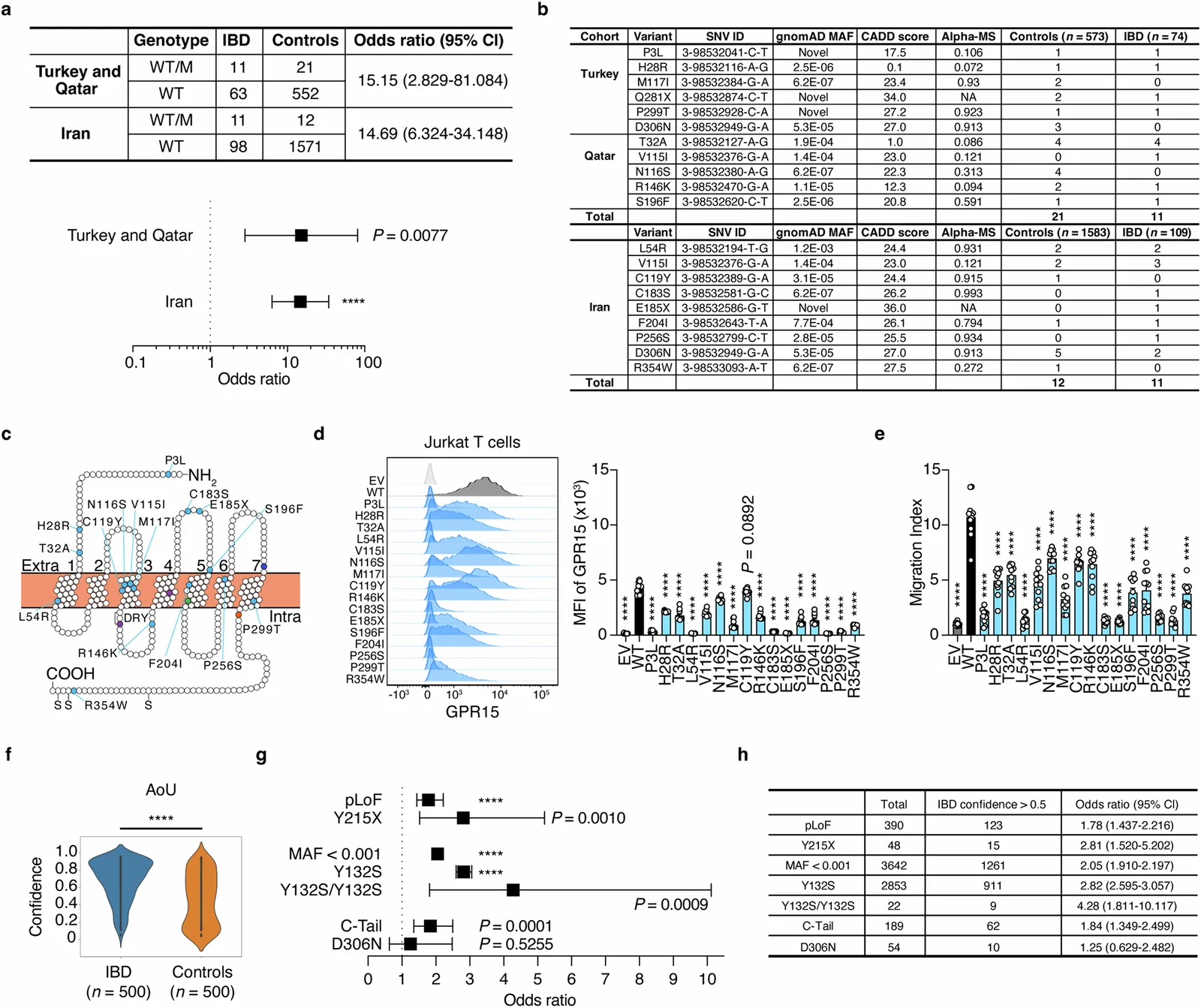
Deleterious GPR15 variants are associated with IBD in human cohorts. **a**, Table (upper) and forest plots (lower) of odds ratios with 95% confidence intervals (CIs) in Turkish/Qatari and Iranian cohorts, calculated using generalized linear mixed-effects logistic regression (Turkish/Qatari, family as a random effect) or multivariable logistic regression (Iranian) adjusted for age, sex, ethnicity or top 10 principal components (Iranian) of genomic sequencing single-nucleotide variants (SNVs). **b**, Bioinformatic summary of rare GPR15 variants in Turkish/Qatari and Iranian cohorts excluding people with known inborn errors of immunity. **c**, Schematic of GPR15 showing patient variants in Turkish/Qatari and Iranian cohorts. **d, e**, Flow cytometry of GPR15 surface staining (**d**) and migration toward GPR15L (**e**) in Jurkat T cells transduced with WT, patient mutants in **b** or EV. **d**, right: MFI quantified. *n* = 11 per group. **f-h**, Association of GPR15 variants with IBD-related phenotypes in the All of Us cohort using machine learning on people with whole exome sequencing (WES) and electronic health record (EHR) data. **f**, Violin plots of confidence score in people with IBD codes (*n* = 500) and no-IBD codes (*n* = 500). Forest plots (**g**) and table (**h**) show odds ratios (95% CIs) for predicted loss-of-function (pLoF) variants, rare missense variants (MAF < 0.001), predicted damaging variants (CADD > 20) in the C-terminal region (G285–V358) and selected patient-derived SNVs. Odds ratios were calculated with multivariable logistic regression adjusted for age, sex, race, ethnicity and top 10 principal components for genomic sequencing of SNVs. Data are representative (**d**, left) or mean ± s.e.m. (**d**, right, **e**) pooled from 3 independent experiments. Square, point estimate of odds ratios; horizontal bars, 95% CIs (**a**, **g**). Statistical significance was determined by two-sided Wald tests (**a**, **g**), one-way ANOVA with Dunnett’s multiple-comparisons test (**d**, right, **e**) or two-sided Mann–Whitney *U*-test (**f**). *P* values in (**g**) were adjusted for multiple comparisons using the Benjamini–Hochberg false discovery rate (FDR) method. ****P < 0.0001. Source data

To evaluate *GPR15* genotype risk for IBD, we performed a retrospective analysis in Turkish and Qatari family-based cohorts and an Iranian population-based cohort. The family cohorts included 647 people (74 with IBD), and the Iranian cohort included 1,692 people (109 with IBD). Comparison with control individuals without IBD identified 18 unique rare monoallelic *GPR15* variants (MAF < 0.001) that were associated significantly with IBD (Extended Data Fig. 4a,b). Most variants reduced GPR15 surface expression, except for C119Y, and all impaired GPR15L-mediated migration (Fig. 1g,h and Extended Data Fig. 4c–e). At the population level, predicted loss-of-function (pLOF) variants in *GPR15* were among the top-associated variants for a colon-related phenotype (endoscopic extirpation of lesion of colon; *β* = 0.216, SKAT-O (optimal sequence kernel association test) *P* = 7.15 × 10^−5^) in the Genebass UK Biobank analysis^25^. In the All of Us (AoU) cohort (more than 250,000 people), the aggregated burden of pLOF variants was associated significantly with ulcerative colitis (*β* = 2.17; SKAT-O *P* = 0.03)^26^, one of the classic IBD diagnostic categories. To capture underdiagnosed cases, we developed a machine learning model for predicting IBD-related phenotypes using AoU records. After training, IBD-related phenotype confidence scores produced by the model were significantly higher in people with IBD codes than in those without (Extended Data Fig. 4f). Using this model, pLoF variants (including Y215X), the Y215X variant itself, rare missense variants (MAF < 0.001), Y132S variants and homozygous Y132S/Y132S variants were all associated significantly with IBD-related phenotypes (confidence score > 0.5), whereas the D306N variant was not, probably owing to low carrier numbers (Extended Data Fig. 4g,h). Aggregation of predicted deleterious variants (combined annotation-dependent depletion (CADD) scores > 20) in the C-terminal tail region (G283–V358) (including D306N) was associated significantly with the IBD-related phenotypes (Extended Data Fig. 4g,h). Together, the cohort and population studies associated deleterious GPR15 variants with IBD and related phenotypes.

## GPR15 guides CD8^+^ T cells to the colon

**Fig. 2:**
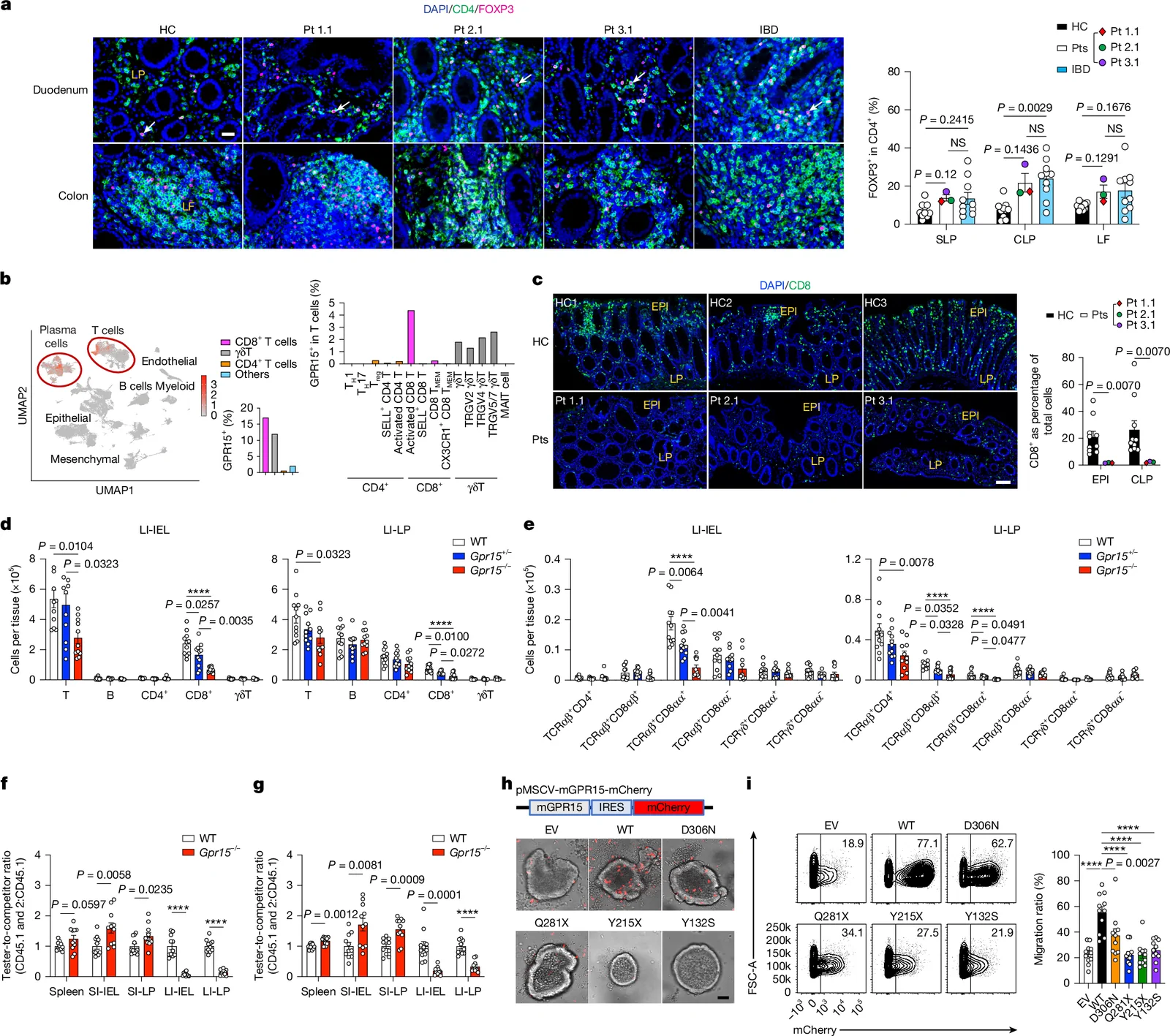
GPR15 regulates CD8α^+^ T cell migration to colon. **a**, Left, immunofluorescence of T_reg_ cells (arrows: CD4^+^FOXP3^+^) in duodenal and colonic biopsies from healthy control individuals (*n* = 10), Pt 1.1, Pt 2.1, Pt 3.1 and patients with sporadic IBD (*n* = 10); 4′,6-diamidino-2-phenylindole (DAPI, blue); CD4 (green) and FOXP3 (magenta). Right, FOXP3^+^ cell fractions among CD4^+^ T cells in small intestinal LP (SLP), CLP and LF. **b**, Uniform manifold approximation and projection (UMAP) of healthy human intestinal scRNA-seq showing GPR15^+^ cells across seven clusters. Middle, GPR15^+^ fraction in indicated cells. Right, GPR15^+^ T cell subset fractions among T cells. **c**, Left, CD8 staining in colonic biopsies from healthy individuals (*n* = 10), Pt 1.1, Pt 2.1 and Pt 3.1. Right, CD8^+^ T cell fractions in EPI and LP. **d**,**e**, Lymphocyte (**d**) and IEL subset (**e**) cell numbers in LI-IEL and LI-LP of WT, *Gpr15*^+/−^ and *Gpr15*^−/−^ mice at 2–3 months old. **f**,**g**, Tester-to-competitor ratios of TCRαβ^+^CD8αα^+^ (**f**) and TCRαβ^+^CD8αβ^+^ cells (**g**) in *Rag2*^−/−^*Il2rg*^−/−^ mice reconstituted with CD45.1^+^CD45.2^+^ tester cells (WT, *Gpr15*^−/−^) and WT CD45.1^+^ competitors. **h**, Top, mouse GPR15 retroviral vector with mCherry; bottom, confocal images of *Gpr15*^−/−^ CD8^+^ T cells expressing WT, mutants or EV, co-cultured with intestinal organoids. Red, mCherry^+^ cells. **i**, Flow cytometry of mCherry^+^ T cells in organoids from **h**. Right, migration ratio = (mCherry^+^ cells in organoids)/(total mCherry^+^ cells). Data are representative (**a**,**c**,**i**, left; **h**) or mean ± s.e.m. for three to ten people (**a**,**c**, right) or pooled from three to five independent experiments (**d**–**g**; **i**, right); *n* = 11 per group (**d**–**i**). Statistics, Kruskal–Wallis with Dunn’s test (**a**, right), two-sided Mann–Whitney *U*-test (**c**, right), one-way ANOVA with Tukey’s (**d**,**e**) or Dunnett’s (**i**, right) test or unpaired two-sided *t*-test (**f**,**g**). ****P < 0.0001; NS, *P* > 0.9999. Scale bars, 25 μm (**a**), 100 μm (**c**) and 30 μm (**h**). LF, lymphoid follicles; IRES, internal ribosome entry site. Source data

**Extended Data Fig. 5:**
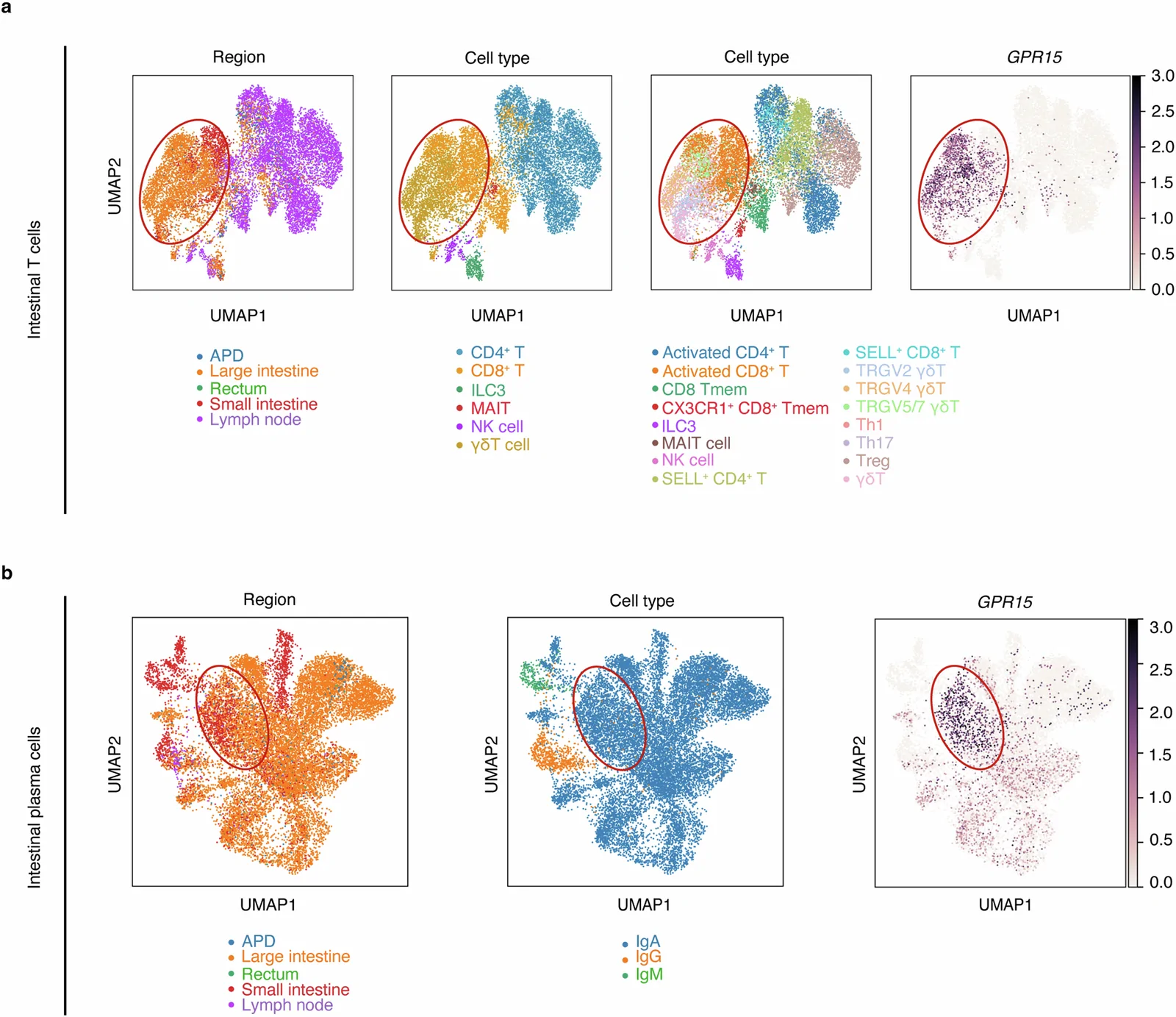
GPR15 is highly expressed in CD8^+^ T cells, γδ T cells and IgA plasma cells in the human gut. **a, b**, UMAPs and feature plots from healthy human intestinal single-cell RNA-seq (https://www.gutcellatlas.org/) showing GPR15^+^ cells in T cells (**a**) and plasma cells (**b**) across 5 human intestinal regions (left). APD: accessory pancreatic duct. Middle, unsupervised clustering identified 16 T cell clusters (**a**) and 3 plasma cell clusters (**b**). Right, feature plots showing *GPR15* expression with the densest region circled in red.

To investigate the impact of reduced GPR15 on intestinal lymphocyte migration, we analysed intestinal biopsies from three patients with GPR15 biallelic mutations. Despite the role of GPR15 in mouse CD4^+^ T_reg_ cell migration^8^, we observed a high FOXP3^+^ T_reg_ cell ratio among CD4^+^ T cells in patient duodenal and colonic biopsies. These frequencies mirror the increased CD4^+^ T_reg_ cell levels typically found in the inflamed colonic lamina propria (CLP) of patients with sporadic IBD^14^ (Fig. 2a). Thus, GPR15 seems to be dispensable for intestinal homing of CD4^+^ T_reg_ cells in humans. Analysis of published single-cell RNA sequencing (scRNA-seq) data from human intestinal specimens^27^ showed that *GPR15* mRNA was enriched in CD8^+^ T cells, γδ^+^ T cells and plasma cells, but expressed in only a small fraction of CD4^+^ T_reg_ cells. Among T cells, expression was highest in activated CD8^+^ T cells (Fig. 2b). GPR15^+^ T cells were found mainly in the colon, whereas GPR15^+^ IgA plasma cells were present in both the small intestine and large intestine (Extended Data Fig. 5a,b). CD8^+^ T cells were markedly reduced in both the epithelium (EPI) and lamina propria (LP) of colonic biopsies from the three patients compared with control individuals (Fig. 2c). Therefore, in humans, the GPR15–GPR15L axis governs colonic migration of a class of CD8^+^ T cells, not CD4^+^ T_reg_ cells.

**Extended Data Fig. 6:**
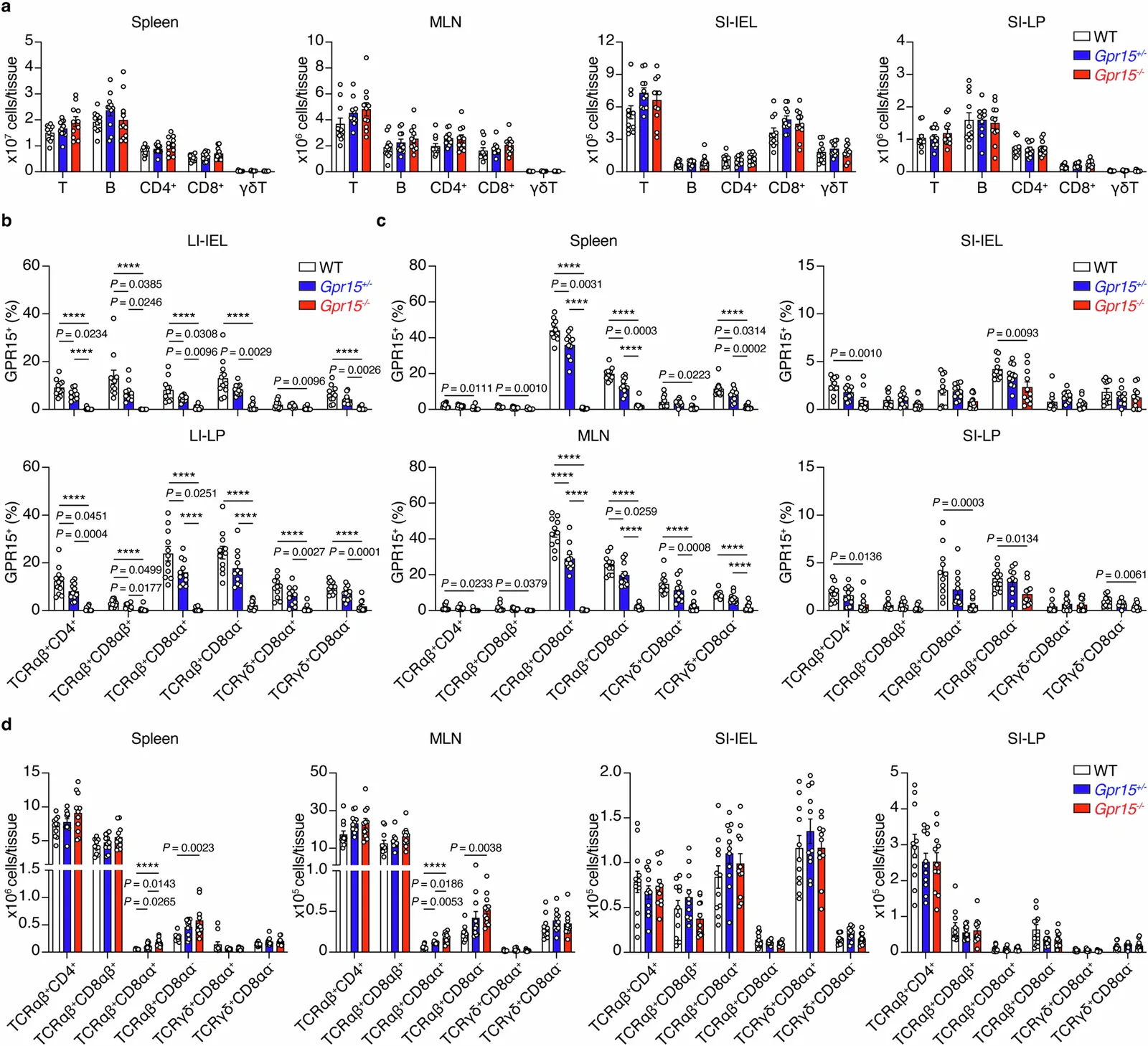
Immunophenotyping in *Gpr15*^*−/−*^ mice. **a**, Numbers of indicated lymphocytes from spleen, mesenteric lymph nodes (MLN), small intestinal epithelium (SI-IEL) or lamina propria (SI-LP) of WT, *Gpr15*^*+/−*^ and *Gpr15*^*−/−*^ mice. **b**, Fractions of GPR15^+^ cells among indicated lymphocytes in large intestinal epithelium (LI-IEL, upper) or lamina propria (LI-LP, lower) of mice in **a**. **c**, Fractions of GPR15^+^ cells among indicated lymphocyte subsets from spleen, MLN, SI-IEL or SI-LP of mice in **a**. **d**, Numbers of indicated lymphocyte subsets in **c**. Data are mean ± s.e.m. pooled from 3-5 independent experiments (**a-d**). Mice were 2 to 3 months old, *n* = 11 per group (**a-d**). Statistical significance was determined by one-way ANOVA with Tukey’s multiple-comparisons test (**a-d**). ****P < 0.0001. Source data

Given this CD8^+^ T cell deficit in patients, we examined *Gpr15*^−/−^ mice and found a striking and selective deficit of CD8^+^ T cells among large intestinal (LI)-IEL and LI-LP lymphocytes, with intermediate reductions in *Gpr15*^*+/−*^ heterozygous mice (Fig. 2d). Other lymphocyte subsets in the LI or spleen, mesenteric lymph nodes (MLN) and small intestine (SI) lymphocyte populations were unaffected (Fig. 2d and Extended Data Fig. 6a). Thus, humans and mice that lack GPR15 share the same defect in colonic mucosal CD8^+^ T cells. Detailed analysis of IEL subsets showed that GPR15^+^ cell percentage was highest in TCRαβ^+^ cells (CD8αα^+^ or CD8αα^−^), followed by TCRγδ^+^, TCRαβ^+^CD4^+^ and TCRαβ^+^CD8αβ^+^ cells in the LI-LP, spleen and MLN, but was low in the SI (Extended Data Fig. 6b,c). In the LI EPI, where GPR15L is highly expressed^12^, surface GPR15 may be underestimated owing to ligand-induced internalization^28^ (Extended Data Fig. 6b). Also, *Gpr15*^*−/−*^ mice showed a marked and selective reduction in TCRαβ^+^CD8αα^+^ cells in the LI-IEL and smaller reductions in TCRαβ^+^CD4^+^, TCRαβ^+^CD8αβ^+^ and TCRαβ^+^CD8αα^+^ cells in the LI-LP. *Gpr15*^*+/−*^ mice showed moderate reductions in TCRαβ^+^CD8αα^+^ cells in the LI-IEL and the LI-LP and of TCRαβ^+^CD8αβ^+^ cells in the LI-LP (Fig. 2e). IEL subsets were unaffected in the SI (Extended Data Fig. 6d). In the spleen and MLN, TCRαβ^+^CD8αα^+^ and TCRαβ^+^CD8αα^−^ cells increased in *Gpr15*^*−/−*^ mice, with moderate increases in TCRαβ^+^CD8αα^+^ cells in *Gpr15*^*+/−*^ mice, suggesting that cells may be misdirected from the colon (Extended Data Fig. 6d).

To examine whether GPR15 regulates migration of TCRαβ^+^CD8αα^+^ cells to the LI EPI, we adoptively transferred a 1:1 mix of allelically marked tester (WT or *Gpr15*^*−/−*^, CD45.1^+^CD45.2^+^) and WT competitor (CD45.1^+^CD45.2^−^) TCRαβ^+^CD8αα^+^ IEL precursors (IELp)^19^ into *Rag2*/*Il2rg*-deficient mice that lack lymphocytes and lymphoid cells. After 2 weeks, we found that WT tester and competitor IELp cells contributed equally to TCRαβ^+^CD8αα^+^ IELs. By contrast, *Gpr15*^*−/−*^ tester IELps generated fewer TCRαβ^+^CD8αα^+^ cells in the LI but more in the SI (Fig. 2f). Similarly, TCRαβ^+^CD8αβ^+^ migration was reduced in the LI but increased in the spleen and SI (Fig. 2g). Finally, to test how patient GPR15 mutations affect migration to the large intestinal EPI, we transduced the corresponding patient variants into *Gpr15*^*−/−*^ mouse CD8^+^ T cells with an mCherry reporter and co-cultured them with WT intestinal organoids. Immunofluorescence showed that WT cells intravasated into organoids more often than mutant cells (Fig. 2h, Supplementary Fig. 1d and Supplementary Videos 1–6). Flow cytometry confirmed reduced migration for all variants (Fig. 2i). Together, these findings indicate that deleterious GPR15 variants cell-autonomously impair CD8^+^ T cell migration to the colonic mucosa.

## Molecular attributes of CD8^+^ T_IGR_ cells

**Fig. 3:**
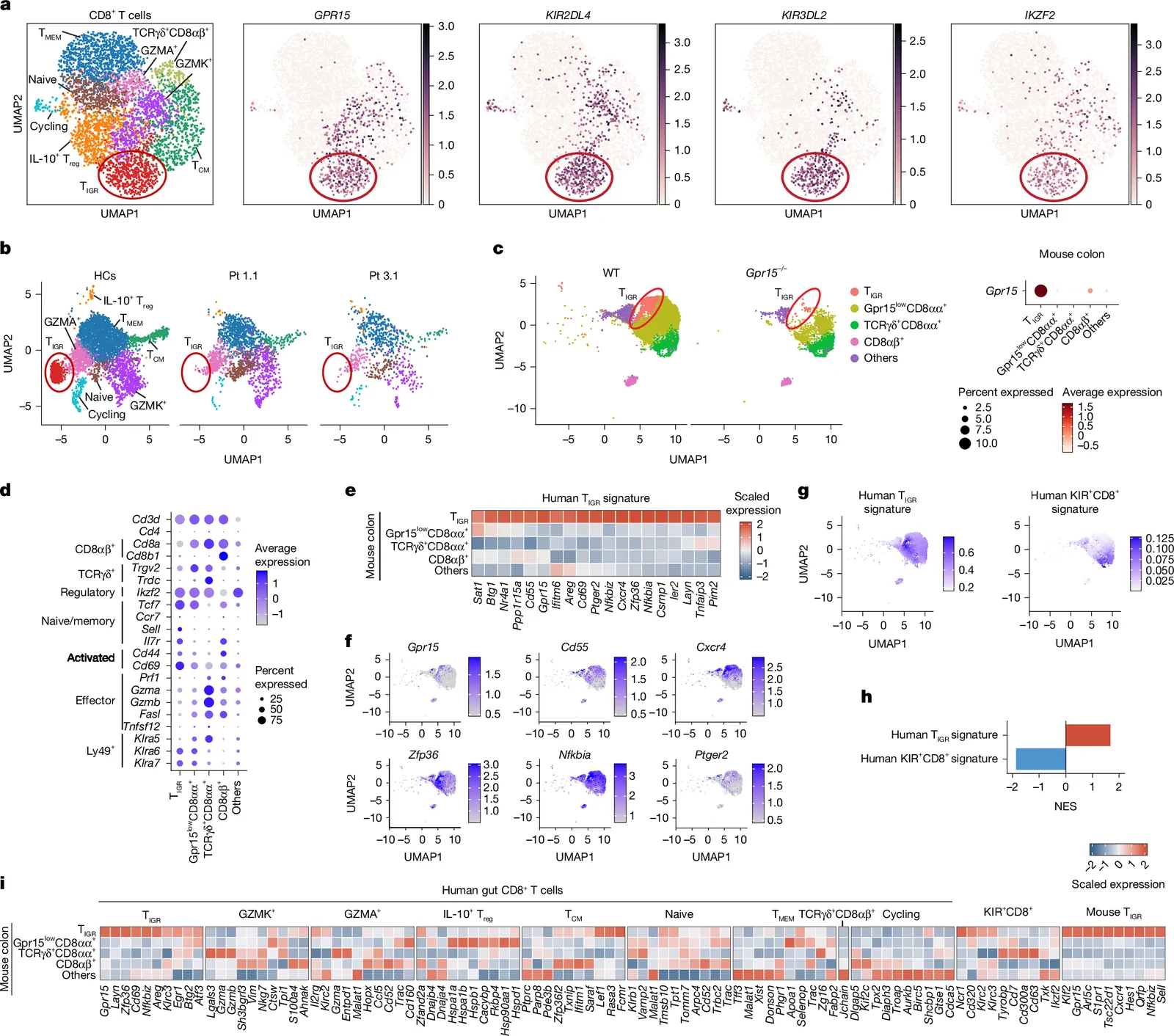
Transcriptomics of CD8^+^ T cells from human and mouse colon. **a**, Left, UMAP of human intestinal scRNA-seq data (https://www.gutcellatlas.org/) showing nine CD8^+^ T cell clusters identified by unsupervised clustering. Right, feature plots show *GPR15*, *KIR2DL4*, *KIR3DL2* and *IKZF2* expression. The intensity scale represents normalized expression. Red ellipse, GPR15^+^ CD8^+^ T_IGR_ subset. **b**, UMAPs of human intestinal scRNA-seq data showing eight CD8^+^ T cell clusters from control individuals (*n* = 16) in **a**, Pt 1.1 and Pt 3.1. The CD8^+^ T_IGR_ population is indicated (red ellipse). **c**, Left, UMAPs of mouse scRNA-seq data showing five CD8α^+^ IEL clusters from WT and *Gpr15*^−/−^ colon. Red ellipse, CD8^+^ T_IGR_ cells. Right, dot plots of *Gpr15* expression in the cells indicated. Dot colour, average expression; size, percentage of cells expressing transcript. **d**, Dot plots of mouse scRNA-seq of selected marker genes in colonic cell types. **e**, Heatmap of mouse scRNA-seq showing shared gene signatures between human and mouse CD8^+^ T_IGR_ in mouse colonic cell types. **f**, Feature plots of mouse scRNA-seq showing expression of selected CD8^+^ T_IGR_ marker genes (*Gpr15*, *Cd55*, *Cxcr4*, *Zfp36*, *Nfkbia* and *Ptger2*) in WT colonic CD8α^+^ IELs. **g**, UMAPs of mouse scRNA-seq showing human CD8^+^ T_IGR_ cell and human KIR^+^CD8^+^ T cell gene signatures in WT colonic CD8α^+^ IELs. **h**, Gene set enrichment analysis (GSEA) of human CD8^+^ T_IGR_ cell and human KIR^+^CD8^+^ T cell gene signatures in mouse CD8^+^ T_IGR_ cells. **i**, Heatmap of top ten genes defining human intestinal CD8^+^ T clusters in **a**, mouse CD8^+^ T_IGR_ cells in **c** and human KIR^+^CD8^+^ T cells mapped onto mouse colonic scRNA-seq clusters. Source data

**Extended Data Fig. 7:**
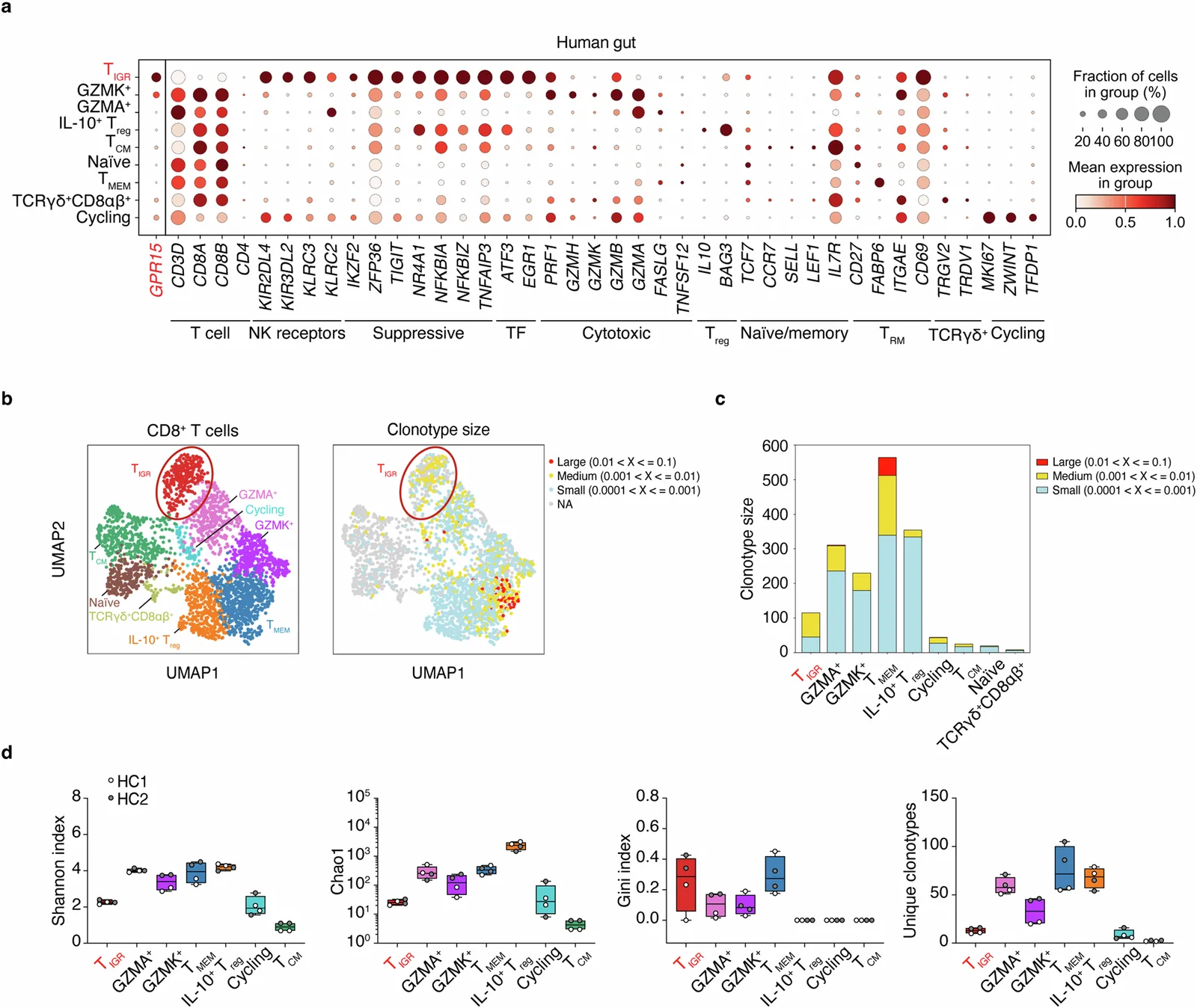
scRNA-seq and scTCR-seq analysis of CD8^+^ T cells in human gut. **a**, Dot plots of published human intestinal scRNA-seq data (https://www.gutcellatlas.org/) showing expression and cellular detection of marker genes across 9 CD8^+^ T cell clusters identified by unsupervised clustering. Dot color, average expression; size, percentage of cells expressing transcript. T_reg_, regulatory T cells; T_CM_, central memory T cells; T_MEM_, memory T cells; T_RM_, tissue-resident memory T cells; TF, transcription factor. **b**, Left, UMAP of CD8^+^ T cells from healthy human intestinal biopsies (*n* = 2) showing 9 clusters. Right, UMAP of T cell clone expansion from scTCR-seq, colored by clonotype size to show expanded clonotype distribution. CD8^+^ T_IGR_ cells are encircled in red. **c**, Distribution of clonotype sizes across clusters in **b**. **d**, Box plots of TCR diversity metrics (Shannon, Chao1 and Gini) and numbers of unique clonotypes in the indicated CD8^+^ T cell subsets from 4 subjects (2 healthy donors in **b**, 2 subjects per donor), evaluated by VDJtools. The box plots show the median (center line), 25^th^ and 75^th^ percentiles (box limits), whiskers to minima and maxima within 1.5 times the interquartile range and outliers beyond this range (points). Source data

We analysed published human intestinal scRNA-seq data^27^ to define the CD8^+^ T cell subsets and identified nine reproducible clusters in control samples. One cluster comprised GPR15^+^ intestinal CD8^+^ T cells, whereas the other eight contained smaller *GPR15* fractions: CD8^+^ memory (T_MEM_), central memory (T_CM_), naive, GZMA^+^ effector, GZMK^+^ effector, IL10^+^ T_reg_, cycling and TCRγδ^+^CD8αβ^+^ cells (Fig. 3a and Extended Data Fig. 7a). Although the GPR15^+^ cluster did not fit a previously identified phenotype, it expressed KIR2DL4 and KIR3DL2, which are highly expressed in peripheral KIR^+^CD8^+^ regulatory T cells in autoimmune disease and infection^16^ (Fig. 3a and Extended Data Fig. 7a). The GPR15^+^ cluster expressed higher levels of other natural killer (NK)-associated (for example, *KLRC3* and *KLRC2*), tissue residency (for example, *CD69* and *ITGAE*), immunosuppressive (for example, *IKZF2*, *ZFP36*, *TIGIT*, *NR4A1*, *NFKBIA*, *NFKBIZ* and *TNFAIP3*), early-response transcription factor (for example,* ATF3* and *EGR1*) and memory T cell genes, with lower expression of naive T cell genes (Extended Data Fig. 7a). Moreover, scRNA-seq analysis of intestinal biopsies from Pt 1.1 and Pt 3.1 showed that the GPR15^+^ cluster was absent compared with 16 published control datasets^27^ (Fig. 3b). As the GPR15^+^ cluster identifies a unique CD8^+^ T cell subset that requires GPR15 for localization to the EPI and LP portions of the mucosa and possibly controls intestinal inflammation, we termed the subset ‘intramucosal GPR15-guided regulatory CD8^+^ T cells’ (CD8^+^ T_IGR_). TCR repertoire analysis from two control individuals showed that human CD8^+^ T_IGR_ have oligoclonal expansions that are enriched for medium- and small-size clonotypes (Extended Data Fig. 7b,c). Clonal diversity analyses showed that CD8^+^ T_IGR_ had a lower Shannon index, Chao1 richness and number of unique clonotypes, but a higher Gini index than GZMA^+^ and GZMK^+^ effector cells (Extended Data Fig. 7d). Thus, the TCR repertoire of CD8^+^ T_IGR_ is diverse, but less so than other CD8^+^ subsets.

**Extended Data Fig. 8:**
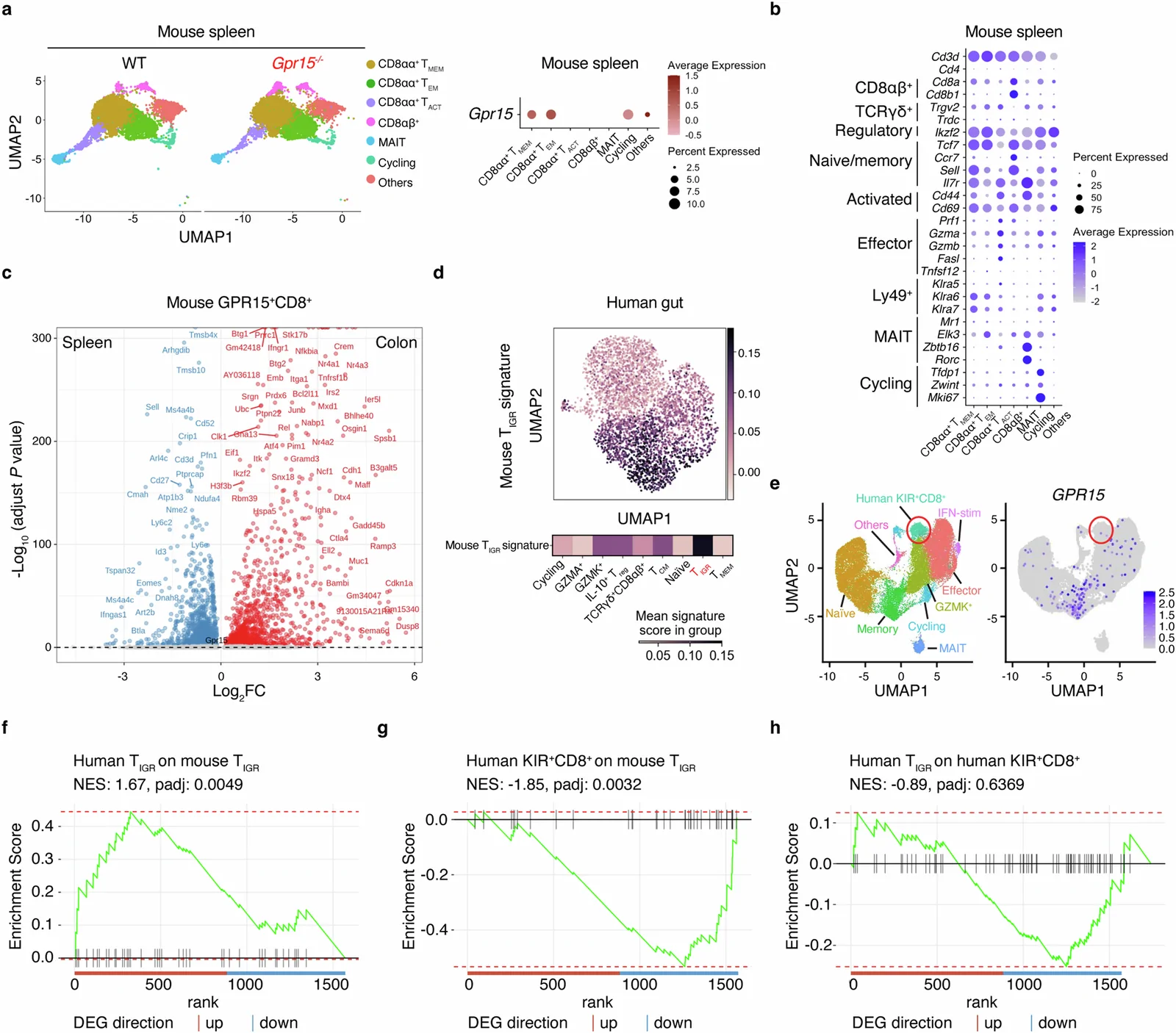
scRNA-seq analysis of CD8^+^ T cells in mouse spleen and human gut. **a**, Left, UMAP of mouse scRNA-seq data showing 7 CD8αα^+^ T cell clusters from WT or *Gpr15*^*−/−*^ spleens identified by unsupervised clustering. Right, Dot plots of *Gpr15* expression across indicated cell types. **b**, Dot plots of mouse scRNA-seq showing expression and cellular detection rate of selected cluster marker genes in mouse spleen. **c**, Volcano plot of differentially expressed genes between colonic T_IGR_ cells (red) and splenic GPR15^+^CD8αα^+^ T cells (combined CD8αα^+^ T_MEM_ and CD8αα^+^ T_EM_, blue). Differentially expressed genes identified using a two-sided Wilcoxon Rank Sum test, with p-values adjusted for multiple comparisons using Bonferroni correction. Adjust *p* < 0.05. **d**, Feature plots and heatmap of human scRNA-seq showing expression of the mouse CD8^+^ T_IGR_ signature across human intestinal CD8^+^ T cell types. **e**, Left, UMAP of published human scRNA-seq data showing 9 CD8^+^ T cell subpopulations in blood from HC (*n* = 10), multiple sclerosis patients (*n* = 6) and COVID-19 patients (*n* = 25) identified by unsupervised clustering^16^. Right, feature plots of *GPR15* expression. Intensity scale, normalized gene expression. The human KIR^+^CD8^+^ T cell subset (red circle) lacks GPR15 expression. **f-h**, Gene Set Enrichment Analysis of human CD8^+^ T_IGR_ (**f**) or human KIR^+^CD8^+^ T cell (**g**) gene signatures in the mouse CD8^+^ T_IGR_ cluster and human CD8^+^ T_IGR_ signature in the human KIR^+^CD8^+^ T cells (**h**). Normalized enrichment score (NES) calculated using a weighted Kolmogorov–Smirnov-like running sum statistic, with empirical p-values computed via an adaptive multi-level Monte-Carlo scheme and corrected for multiple testing using the Benjamini–Hochberg procedure. Padj, adjusted *P* value.

To characterize the mouse counterpart of human CD8^+^ T_IGR_, we performed scRNA-seq on WT or *Gpr15*^*−/−*^ CD8^+^ LI-IELs and TCRαβ^+^CD8αα^+^ splenocytes. We found that cells segregated by organ and genotype (Fig. 3c and Extended Data Fig. 8a). In WT mice, unsupervised clustering identified five colonic CD8^+^ T cell clusters: T_IGR_, *Gpr15*^low^CD8αα^+^, TCRγδ^+^CD8αα^+^, CD8αβ^+^ and others (Fig. 3c,d). The CD8^+^ T_IGR_ cluster expressed the highest *Gpr15* levels, and this cluster was reduced greatly in *Gpr15*^*−/−*^ mice (Fig. 3c). The CD8^+^ T_IGR_ cluster expressed CD8α chains, often without CD8β, suggesting similarity to CD8αα^+^ colitis-protective T cells^20^ (Fig. 3d). The CD8αβ^+^ and *Gpr15*^low^CD8αα^+^ clusters expressed *Gpr15* weakly and were slightly reduced in *Gpr15*^*−/−*^ mice. By contrast, splenic TCRαβ^+^CD8αα^+^ cells identified seven clusters—CD8αα^+^ memory (T_MEM_), CD8αα^+^ effector memory (T_EM_), activated CD8αα^+^ (T_ACT_), CD8αβ^+^, mucosal-associated invariant T cell (MAIT), cycling T cells and others—but no CD8^+^ T_IGR_ cluster (Extended Data Fig. 8a,b). Although *Gpr15* was expressed in CD8αα^+^ T_MEM_, CD8αα^+^ T_EM_, cycling T cells and others, their cluster sizes were unaffected in *Gpr15*^*−/−*^ mice, implying a specific effect on CD8^+^ T_IGR_. Differential expression analysis of colonic CD8^+^ T_IGR_ versus splenic GPR15^+^CD8αα^+^ T cells (CD8αα^+^ T_MEM_ and CD8αα^+^ T_EM_) from WT mice identified 1,227 and 1,331 significantly upregulated genes, respectively (adjusted *P* < 0.05) (Extended Data Fig. 8c). The colonic T_IGR_ cluster upregulated *Ikzf2* (encoding HELIOS) and the nuclear orphan receptor genes *Nr4a1*, *Nr4a2* and *Nr4a3*, key regulators of T_reg_ differentiation and function. By contrast, splenic GPR15^+^CD8αα^+^ T cells expressed memory-associated genes (*Sell*, *Cd27*, *Eomes* and *Id3)*, but *Gpr15* expression did not differ between these tissues.

We next compared transcriptional profiles of mouse and human T_IGR_ clusters. Mouse T_IGR_ expressed high levels of Ly49 receptor genes (for example, *Klra6* and *Klra7*), which are homologues of human KIR genes^16^ (Fig. 3d). Gene signature analysis based on upregulated genes revealed a shared signature between mouse and human T_IGR_ clusters, including surface markers (for example, *Cd55*, *Cxcr4* and *Ptger2*), T cell activation genes (for example, *Cd69*) and immunosuppressive genes (for example, *Zfp36*, *Nfkbia*, *Nfkbiz*, *Tnfaip3 *and *Nr4a1*) (Fig. 3e,f). These genes were expressed less in non-T_IGR_ CD8^+^ clusters in the mouse colon (Fig. 3e) and enriched in the human T_IGR_ cluster relative to other intestinal CD8^+^ T cell clusters (Extended Data Fig. 8d). Previously defined peripheral regulatory KIR^+^CD8^+^ T cells^16^ do not express *GPR15*, indicating that T_IGR_ represents a distinct CD8^+^ T cell population (Extended Data Fig. 8e). Furthermore, the mouse T_IGR_ cluster showed enrichment of the human T_IGR_ signature but not the KIR^+^CD8^+^ T cell signature (Fig. 3g,h and Extended Data Fig. 8f,g). KIR^+^CD8^+^ T cells also lacked enrichment of the human T_IGR_ signature (Extended Data Fig. 8h). To further compare mouse T_IGR_ with human T_IGR_ and KIR^+^CD8^+^ T cells, we mapped the top genes defining human intestinal CD8^+^ clusters onto mouse colonic scRNA-seq clusters (Fig. 3i). Mouse T_IGR_ showed the highest expression of the ‘mouse T_IGR_’ signature and strong enrichment of the ‘human T_IGR_’ signature, confirming conserved transcriptional programmes. Although the mouse T_IGR_ cluster expresses some hallmark genes of KIR^+^CD8^+^ T cells (for example, *Klrc2/3*, *Ncr1*), overall similarity to KIR^+^CD8^+^ T cells was low. Thus, T_IGR_ cells are conserved evolutionally in humans and mice and are molecularly distinct from the previously described KIR^+^CD8^+^ T cells^16^.

## GPR15 loss in CD8^+^ T cells causes colitis

**Fig. 4:**
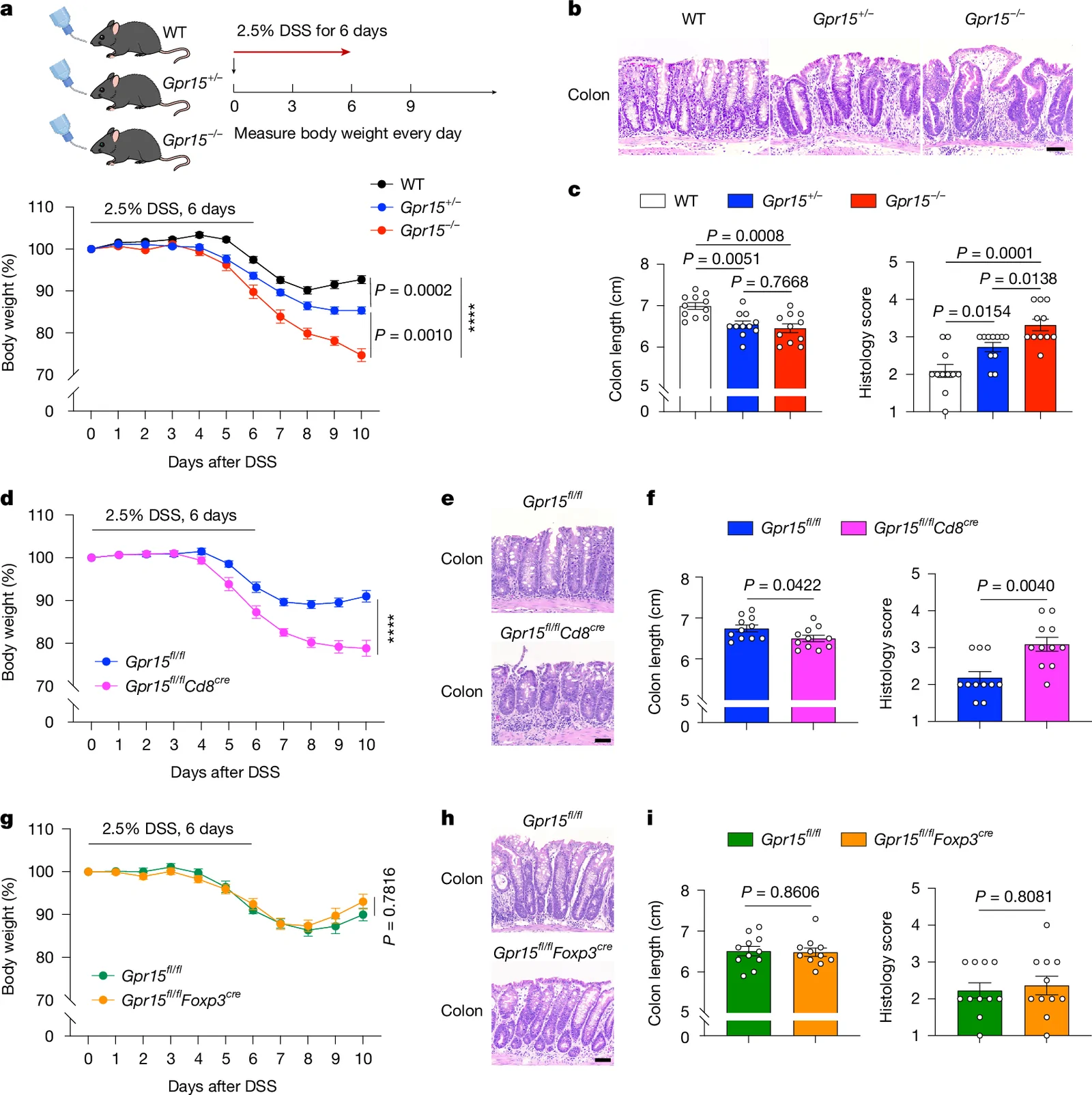
CD8^+^ T_IGR_ cells, rather than FOXP3^+^ T cells, are essential to prevent colitis. **a**–**c**, DSS-induced colitis in WT, *Gpr15*^+/−^ and *Gpr15*^−/−^ mice. **a**, Top, experimental schematic; bottom, body weight changes as a function of time. **b**, Representative H&E-stained colonic sections on day 10. **c**, Colon length (left) and histological scores (right) on day 10. **d**–**f**, DSS-induced colitis in *Gpr15*^*fl/fl*^ and *Gpr15*^*fl/fl*^*Cd8*^*cre*^ mice. **d**, Body weight changes as a function of time. **e**, Representative H&E-stained colonic sections on day 10. **f**, Colon length and histological scores on day 10. **g**–**i**, DSS-induced colitis in *Gpr15*^*fl/fl*^ and *Gpr15*^*fl/fl*^*Foxp3*^*cre*^ mice. **g**, Body weight changes as a function of time. **h**, Representative H&E-stained colonic sections on day 10. **i**, Colon length and histological scores on day 10. Data are representative (**b**,**e**,**h**) or mean ± s.e.m. pooled from three independent experiments (**a**,**c**,**d**,**f**,**g**,**i**); *n* = 11 per group (**a**–**i**). Statistics, two-way ANOVA (**a**, bottom; **d**,**g**), one-way ANOVA with Tukey’s test (**c**, left), two-sided Mann–Whitney *U*-test (**c**,**f**,**i**, right), or unpaired two-sided *t*-test (**f**,**i**, left). ****P < 0.0001. Scale bars, 50 μm. Illustrations in **a** created in BioRender; Cui, J. https://biorender.com/x1n4a8m (2026). Source data

Unlike CD4^+^ T_reg_ cells, CD8^+^ T_IGR_ cells express NK receptors, reflecting innate-like features. Both TCRαβ^+^CD8αα^+^ and Ly49^+^ regulatory CD8^+^ T cells possess innate-like characteristics, enabling rapid suppression of immune responses^29–31^. To explore the functional consequences of GPR15 deficiency during innate immune activation in gut mucosa, we used a dextran sodium sulfate (DSS)-induced colitis model, which triggers epithelial injury and innate inflammation in the early stages^32,33^. Following 2.5% DSS, *Gpr15*^*−/−*^ mice showed accelerated weight loss, increased inflammation, disrupted colonic architecture and greater colon shortening compared with WT mice, whereas *Gpr15*^*+/−*^ mice showed intermediate changes (Fig. 4a–c). These findings suggested that CD8^+^ T_IGR_ cells suppress innate inflammation-driven colitis. Because GPR15 has also been implicated in CD4^+^ T_reg_ cell homing and immunosuppression in the mouse colon^8^, we next assessed DSS-induced colitis in mice with *Gpr15* selectively deleted in CD8^+^ T cells (*Gpr15*^*fl/fl*^*Cd8*^*cre*^) or T_reg_ cells (*Gpr15*^*fl/fl*^*Foxp3*^*cre*^). We observed worsened DSS-induced colitis after selective deletion of *Gpr15* from CD8^+^ T cells (Fig. 4d–f) but not from T_reg_ cells (Fig. 4g–i), supporting a protective role for CD8^+^ T_IGR_ cells in intestinal inflammation.

## CD8^+^ T_IGR_s kill macrophages through FasL and TWEAK

**Fig. 5:**
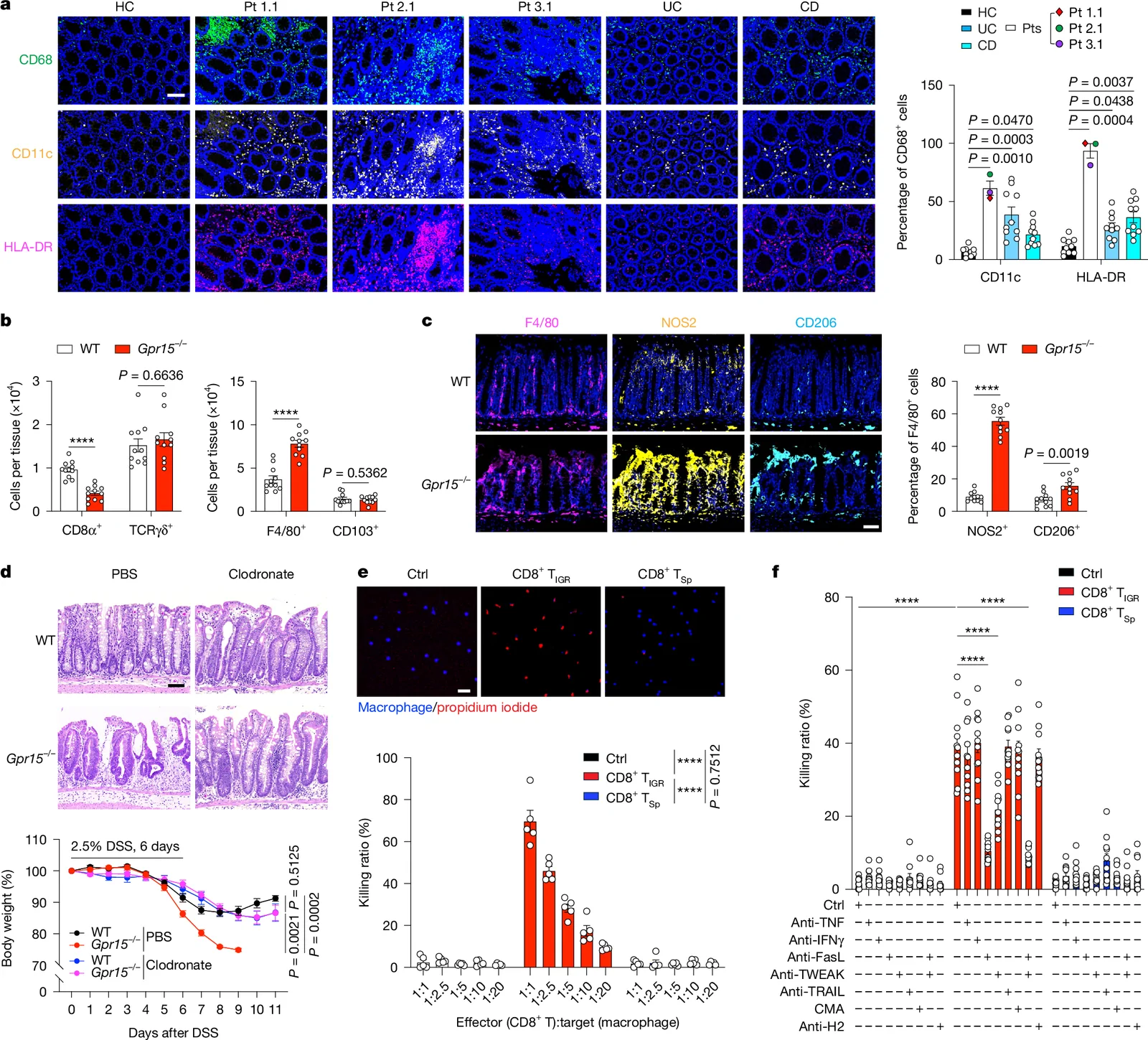
Macrophages that accumulate in colitis can be killed by CD8^+^ T_IGR_ cells by death receptor signalling. **a**, Left, immunofluorescence of CD68, CD11c or HLA-DR in representative sections of colonic biopsies from healthy individuals (*n* = 10), Pt 1.1, Pt 2.1, Pt 3.1 and patients with sporadic ulcerative colitis (*n* = 10) or Crohn’s disease (*n* = 10). Right, fractions of CD11c^+^ or HLA-DR^+^ cells among CD68^+^ cells. **b**, Numbers of TCRαβ^+^CD8αα^+^ or TCRγδ^+^ cells (left) and F4/80^+^ macrophages or CD103^+^ dendritic cells (right) in colons of WT and *Gpr15*^−/−^ mice 3 days after DSS treatment. **c**, Left, immunofluorescence of F4/80, NOS2 and CD206 in mouse colonic sections 5 days after DSS treatment. Right, NOS2^+^ or CD206^+^ fractions of F4/80^+^ cells. **d**, DSS-induced colitis in WT and *Gpr15*^−/−^ mice treated with clodronate or control liposomes (PBS). Top, H&E-stained colonic sections 9 days after DSS treatment. Bottom, body weight changes. **e**, LPS-activated macrophages co-cultured with anti-CD3/CD28 bead-activated CD8^+^ T cells from WT mouse colon (CD8^+^ T_IGR_) or spleen (CD8^+^ T_Sp_). Top, images of macrophages (blue) stained for dead cells (propidium iodide, red). Bottom, fractions of dead macrophages after co-culture with CD8^+^ T cells at indicated ratios; *n* = 5 per group. **f**, Fractions of dead macrophages after co-culture with anti-CD3/CD28 bead-activated CD8^+^ T_IGR_ or CD8^+^ T_Sp_ cells (1:2.5) with indicated blocking antibodies or inhibitors. CMA, concanamycin A. Data are representative (**a**,**c**, left; **d**,**e**, top) or mean ± s.e.m. pooled from three to ten people (**a**, right) or three to five independent experiments (**b**; **c**, right; **d**, **e**, bottom; **f**); *n* = 11 per group (**b**–**d**,**f**). Statistics, Kruskal–Wallis with Dunn’s test (**a**, right), unpaired two-sided *t*-test (**b**; **c**, right), two-way ANOVA (**d**,**e**, bottom) or one-way ANOVA with Tukey’s and Dunnett’s tests (**f**). ****P < 0.0001. Scale bars, 50 μm. Source data

**Extended Data Fig. 9:**
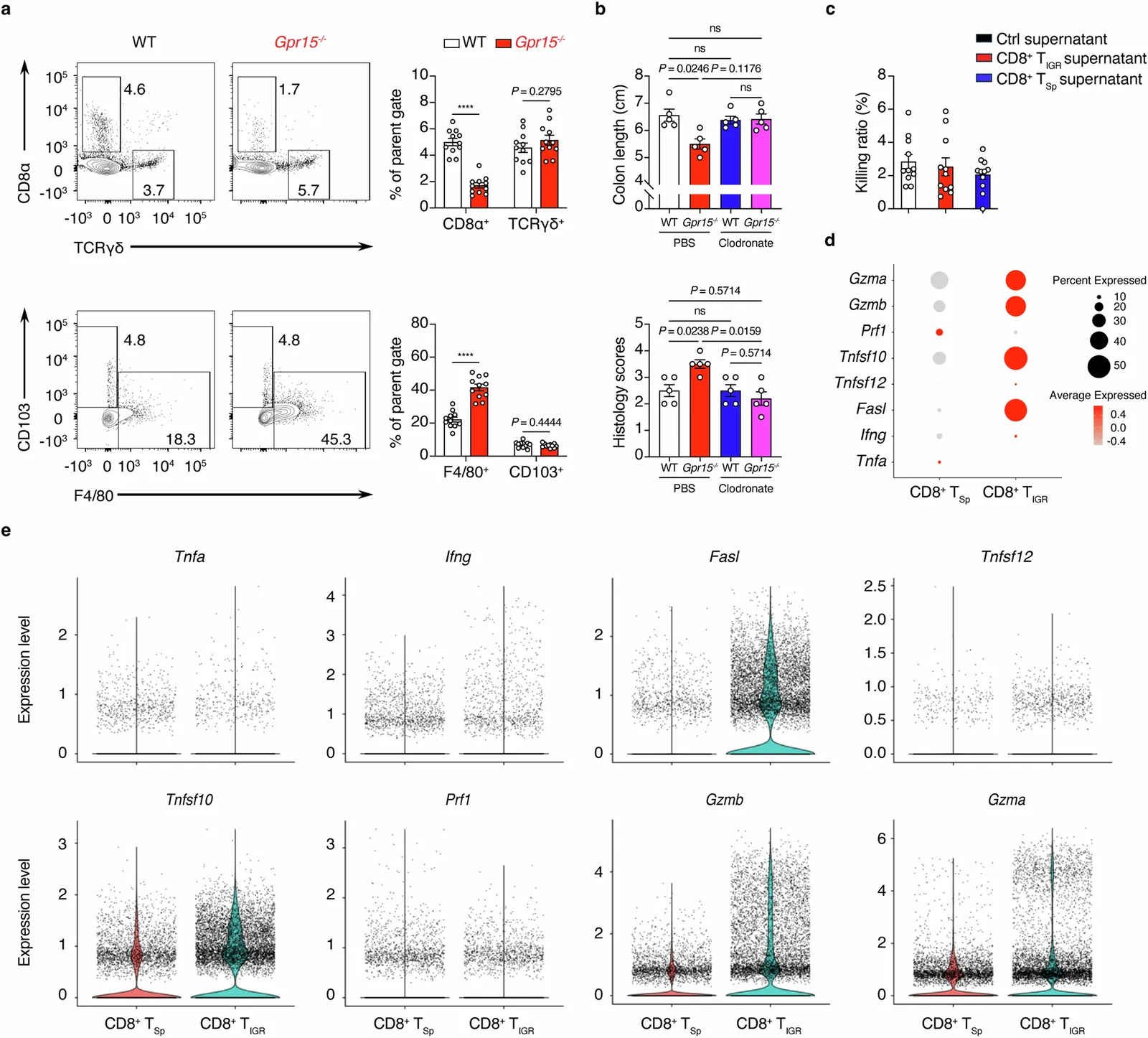
Macrophage accumulation in GPR15-deficiency colitis and death receptor expression in CD8^+^ T_IGR_. **a**, Representative contour plots of TCRαβ^+^CD8αα^+^ or TCRγδ^+^ cells (upper) and F4/80^+^ macrophages or CD103^+^ DCs (lower) from colons of WT and *Gpr15*^*−/−*^ mice 3 days after DSS treatment. Right, percentage of indicated populations quantified. *n* = 11 per group. **b**, DSS-induced colitis in WT and *Gpr15*^*−/−*^ mice treated with clodronate or control liposomes (PBS). Colon length (upper) and histological scores (lower) 9 days after DSS. *n* = 5 per group. **c**, Ratios of dead macrophages after treatment of cultures with supernatant from anti-CD3/CD28 bead-activated CD8^+^ T cells from WT mouse colon (CD8^+^ T_IGR_) or spleen (CD8^+^ T_Sp_) for 24 h. *n* = 11 per group. **d, e**, Dot (**d**) and violin plots (**e**) showing expression of *Tnfa, Ifng, Fasl, Tnfsf12, Tnfsf10, Prf1, Gzmb* or *Gzma* in CD8^+^ T_IGR_ or CD8^+^ T_Sp_ from WT mice. Data are representative (**a**, left) or mean ± s.e.m. pooled from 3-5 independent experiments (**a**, right; **b**, **c**). Statistical significance was determined by unpaired two-sided *t*-test (**a**, right), Kruskal–Wallis with Dunn’s multiple-comparisons test (**b**, upper, **c**) or two-sided Mann–Whitney *U*-test (**b**, lower). Non-significant (ns), *P* > 0.9999; ****P < 0.0001. Source data

In active IBD, macrophages accumulate in large numbers and are key drivers of inflammation^34–36^. Colonic biopsies from Pt 1.1, Pt 2.1 and Pt 3.1 exhibited marked increases in CD68^+^, CD11c^+^ and HLA-DR^+^ macrophages, exceeding levels in samples from patients with sporadic ulcerative colitis and Crohn’s disease (Fig. 5a), which suggests that pro-inflammatory monocyte accumulation may trigger colitis in GPR15-deficient patients. To evaluate whether CD8^+^ T_IGR_ cells suppress these cells, we first examined LI-LP lymphocytes and monocytes in *Gpr15*^*−/−*^ mice 3 days after DSS, before adaptive responses emerge. As in patients, mouse CD8α^+^ T cells were reduced, but other T cell subsets such as TCRγδ^+^ cells were unchanged (Fig. 5b (left) and Extended Data Fig. 9a (upper)). Moreover, F4/80^+^ macrophages were increased, but CD103^+^ dendritic cells were not (Fig. 5b (right) and Extended Data Fig. 9a (lower)). These macrophages showed strong activation with intense NOS2 and CD206 staining (Fig. 5c), suggesting that loss of CD8^+^ T_IGR_ cells permits their expansion. We tested the role of these macrophages in colitis by depleting them with clodronate liposomes and observed attenuated DSS-induced colitis in *Gpr15*^*−/−*^ mice (Fig. 5d and Extended Data Fig. 9b). In vitro, activated mouse CD8^+^ T_IGR_cells, but not splenic CD8^+^ T (CD8^+^ T_Sp_) cells, killed LPS-activated macrophages rapidly (Fig. 5e). Supernatants from CD8^+^ T_IGR_ or CD8^+^ T_Sp_ cells did not induce killing, suggesting a requirement for cell–cell contact (Extended Data Fig. 9c).

We found that CD8^+^ T_IGR_ cells expressed higher levels of cytotoxic mediator transcripts, including *Fasl*, *Tnfsf12*, *Tnfsf10*, *Gzmb* and *Gzma*, than CD8^+^ T_Sp_ cells (Extended Data Fig. 9d,e). To identify how CD8^+^ T_IGR_ cells might kill macrophages, we tested inhibitors of these cytotoxic pathways (Fig. 5f). We found that blocking Fas ligand (FasL) or TNF-related weak inducer of apoptosis (TWEAK) reduced macrophage killing (Fig. 5f). Combined anti-FasL and anti-TWEAK inhibited killing comparable with anti-FasL alone, suggesting a dominant role for FasL. Perforin inhibitor concanamycin or blocking TNF, IFNγ or TRAIL did not inhibit death (Fig. 5f). Blocking class I major histocompatibility complex with anti-H2 antibodies did not affect cytotoxicity, suggesting that killing was class I major histocompatibility complex-independent (Fig. 5f). Together, our data suggested that CD8^+^ T_IGR_ cells prevent colitis by killing inflammatory macrophages through Fas–FasL or TWEAK signalling.

## Potential wider role of CD8^+^ T_IGR_ in IBD

**Extended Data Fig. 10:**
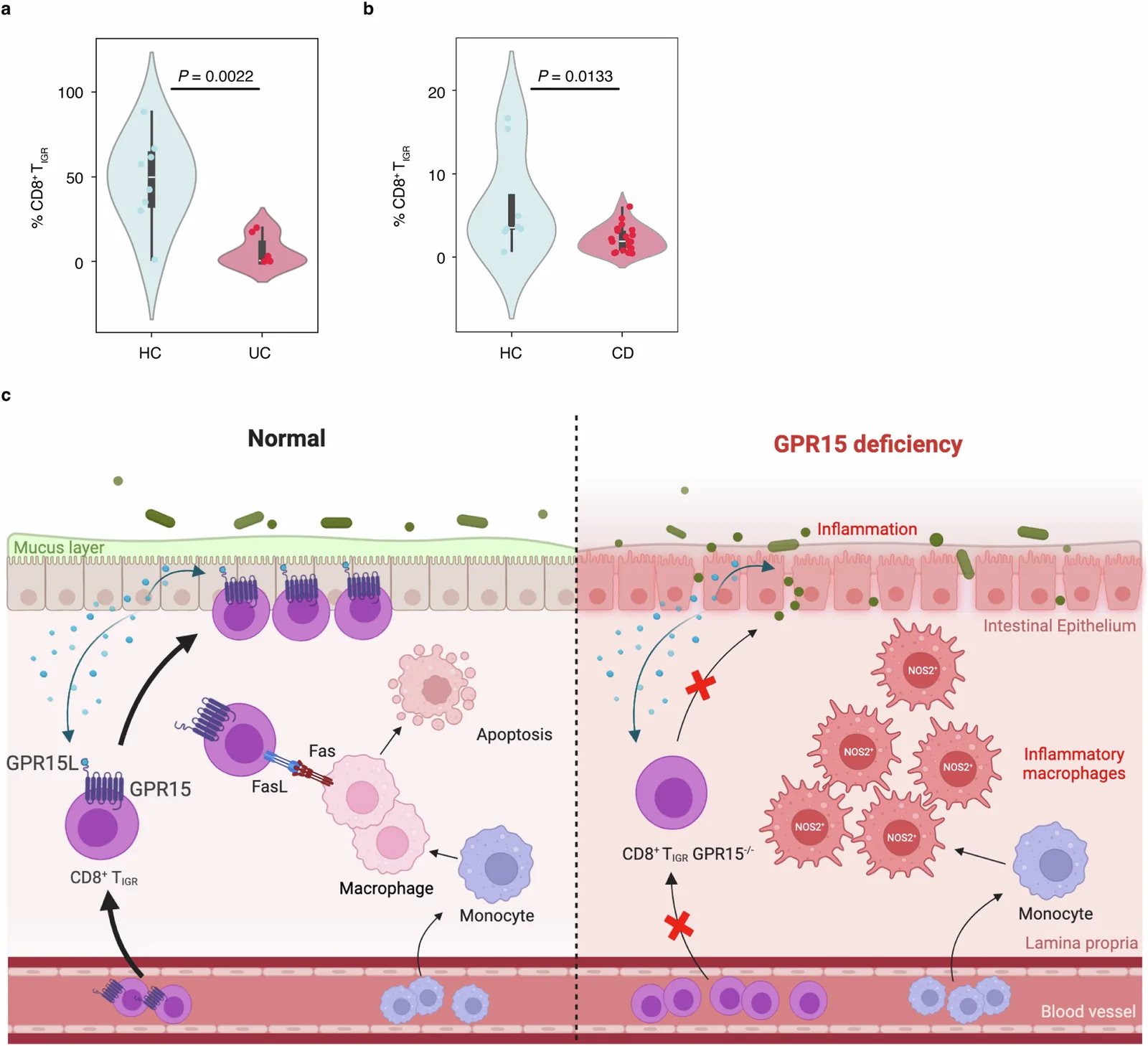
Reduced CD8^+^ T_IGR_ in sporadic IBD and a provisional model of GPR15 function in IBD. **a**, Violin plots of CD8^+^ T_IGR_ ratios in colonic biopsies from a published ulcerative colitis (UC) dataset^37^. HC, *n* = 8; UC, *n* = 7. **b**, Violin plots of CD8^+^ T_IGR_ ratios in intestinal biopsies (terminal ileum) from a published Crohn’s disease (CD) dataset^38^. HC, *n* = 8; CD, *n* = 20. Statistical significance was determined by two-sided Mann–Whitney *U*-test (**a, b**). The box plots show the median (center line), 25^th^ and 75^th^ percentiles (box limits), whiskers to minima and maxima within 1.5 times the interquartile range and outliers beyond this range (points). **c**, Conceptual model of GPR15 regulating CD8^+^ T_IGR_ migration to the colonic mucosa in immune homeostasis. Illustrations in **c** created in BioRender; Lenardo, M. https://biorender.com/rlxiin7 (2025). Source data

To understand the clinical relevance of CD8^+^ T_IGR_ cells, we characterized this population within the intestinal mucosa of patients with sporadic IBD^37,38^. In an ulcerative colitis scRNA-seq dataset, CD8^+^ T_IGR_ cells were reduced by 42% among CD8^+^ T cells in colonic biopsies (Extended Data Fig. 10a). Similarly, CD8^+^ T_IGR_ cells were reduced significantly in terminal ileum biopsies from patients with Crohn’s disease (Extended Data Fig. 10b). These findings suggest that CD8^+^ T_IGR_ cells may contribute to pathogenesis in both forms of sporadic IBD. CD8^+^ T_IGR_ cell reduction could reflect impaired GPR15–GPR15L-mediated migration, limiting local suppression of macrophage-driven inflammation.

## Discussion

When CD8 (Lyt-2) cells were identified by monoclonal antibodies against this cell-surface protein nearly five decades ago, cellular and phenomenological evidence suggested that they contained both immunosuppressive and cytotoxic subsets^39–41^. Molecular analyses confirmed the cytotoxic function, but clarification of CD8^+^ T cells as suppressor or regulatory cells remained elusive^39^. Although the focus has shifted to CD4^+^FOXP3^+^ T_reg_ cells, immunomodulatory functions of CD8^+^ T cells have continued to emerge^16,19,20,42,43^. Although a pro-inflammatory role for GPR15 in human CD4^+^ effector T cells has been proposed^9,44–46^, our finding that monogenic loss-of-function *Gpr15* mutations are associated with early-onset IBD indicates that GPR15 can serve an anti-inflammatory function. GPR15 was previously suggested to mediate CD4^+^ T_reg_ homing and immunosuppression in the mouse colon^8^. However, contrary to our expectation, patients carrying *Gpr15* mutations showed a reduction in CD8^+^ T cells but not CD4^+^ T_reg_ cells in their colons. Human and mouse intestinal scRNA-seq identified *Gpr15* as marking a unique subset of activated CD8^+^ T cells—CD8^+^ T_IGR_ cells—with NK-associated and immunosuppressive features. We found that GPR15 guides CD8^+^ T_IGR_ cell homing to the colonic mucosa in humans and mice through local expression of GPR15 ligand^11,13^. Moreover, CD8^+^ T_IGR_ cells suppress intestinal inflammation by killing activated macrophages, and their loss due to GPR15 deficiency leads to inflammatory macrophage accumulation and colitis. Macrophages are essential for maintaining intestinal homeostasis, yet they transition from peacekeepers to powerful aggressors in IBD by an unknown mechanism^34^. Mechanistically, CD8^+^ T_IGR_ cells induce macrophage death through FasL–Fas and TWEAK signalling. Thus, CD8^+^ T_IGR_ cells show that CD8^+^ T cell suppression exists and can be focused anatomically to control innate rather than adaptive inflammatory processes. The newfound role of GPR15 may provide a molecular approach to improve the prognosis, diagnosis and treatment of specific forms of IBD. Mouse CD8^+^ T_IGR_ cells are a subset of TCRαβ^+^CD8αα^+^ IELs implicated in protection against intestinal inflammation and characterized by restricted TCR repertoires that may recognize self-, diet- or microbiota-derived antigens during epithelial damage and mucosal inflammation^17^. Further research is needed to determine whether similar cell subsets exert an analogous suppressive role in other systems or general autoimmune diseases and whether these cells contribute to host defence against pathogens, and to define the antigens that drive their activation during inflammation.

GPR15-mediated homing of CD8^+^ T_IGR_ cells illuminates how genetic variants can affect a human disease such as IBD (Extended Data Fig. 10c). We find that both biallelic and monoallelic deleterious *Gpr15* variants are associated with IBD with incomplete penetrance. The penetrance of clinical manifestations seems to be affected by age, ancestry and environment. For example, the younger sibling of Pt 1.1, who carries the same compound heterozygous mutations, remained asymptomatic, whereas Pt 2.4 developed IBD only later in childhood. Early-onset IBD is less prevalent in people of African ancestry, which may reflect differences in genetic background^47^. For example, *Nod2* risk alleles are enriched in white compared with African populations^48^. Our studied families span diverse ancestries (Turkish, Kuwaiti and Hispanic). Although the Y215X and Y132S variants of GPR15 are more frequent in people of African ancestry, both Y132S (MAF = 0.0003) and Y215X (MAF < 0.0001) are rare in gnomAD among people of non-African or non-African American ancestry. Moreover, the IBD phenotypes observed in patients with Gpr15 mutations reflect a gene dosage effect, implying an additive co-dominant mode of inheritance. Patients with biallelic loss-of-function mutations developed severe pan-colitis with frequent relapses, whereas those with biallelic missense mutations or monoallelic pLoF mutations that partially impair GPR15L–GPR15 signalling showed moderate, localized IBD. Until now, most of the patients in our study have responded to anti-TNF therapy (Infliximab or Adalimumab), although some have required switching to the integrin inhibitor vedolizumab owing to non-response (Pt 1.1) or adverse effects (Pt 3.1, neutropenia) (Extended Data Fig. 1b). Therefore, controlling pathogenic macrophage activity with either anti-TNF therapy or a chemotaxis inhibitor may benefit patients with GPR15-associated IBD.

GPCRs represent one of the most highly druggable protein families in modern medicine because of their membrane localization, specific architecture and diverse signalling properties^49^. The deficit of CD8^+^ T_IGR_ cells we identify in sporadic ulcerative colitis and Crohn’s disease suggests that targeting the GPR15 pathway may improve T cell control over macrophage-driven inflammation, presenting a potential therapeutic avenue for IBD.

## Methods

### Human participants

Study subjects, including patients, relatives and control individuals, provided written informed consent under National Institutes of Health (NIH) protocol 06-I-0015 (ClinicalTrials.gov NCT00246857), protocol 1500768 approved by the Sidra Medicine and Marmara Medical Center Institutional Review Board (IRBs), and protocol IR.TUMS.CHMC.REC.1398.030 approved by the Tehran University of Medical Sciences Ethics Committee, in accordance with local ethics and IRB regulations. Collection of blood and leftover biopsy samples (collected when medically indicated) is covered by these protocols. Blood samples from anonymized healthy donors were obtained at the NIH Clinical Center under approved protocols. All participants consented to the publication of research results.

### Mice

All animal protocols (ASP nos.: LISB-7E, EIB-104 and EIB-105) were approved by the Animal Care and Use Committee of the National Institute of Allergy and Infectious Diseases (NIAID) or the National Cancer Institute (NCI) in accordance with NIH and Department of Health and Human Services (HHS) guidelines. *Gpr15*^*−/−*^ mice were a kind gift from E. C. Butcher and were generated by backcrossing *B6;129P2-Gpr15*^*tm1.1Litt/J*^ (The Jackson Laboratory, strain no. 008769) to C57BL/6 wild-type mice over 12 generations. *Rag2*^*−/−*^*Il2rg*^*−/−*^ mice (line no. 4111), CD45.1 C57BL/6 mice (line no. 4007), and CD45.2 C57BL/6 mice (line no. B6) were purchased from Taconic. *Gpr15*^*fl/fl*^ (strain no. 028324), *CD8-cre* (strain no. 008766), and *Foxp3-cre* (strain no. 016959) mice were purchased from The Jackson Laboratory. Mice were housed in a specific pathogen-free facility under a 12-h light:dark cycle at 22 ± 2 °C with 70% humidity. After weaning, mice were separated by sex and co-housed by genotype. Sex-matched male and female littermates aged 8–12 weeks were used for all experiments. Sample sizes were not predetermined by statistical calculation. Mice were assigned randomly to experimental groups, and investigators were not blinded.

### Cell lines

HEK 293T cells (catalogue no. CRL-3216), Jurkat T cells, clone E6-1 (catalogue no. TIB-152) and HeLa 229 cells (catalogue no. CCL-2.1) were purchased from the American Type Culture Collection (ATCC). Lenti-X 293T cells (Takara Bio, catalogue no. 632180) and Platinum-E (Plat-E) cells (Cell Biolabs, catalogue no. RV-101) were purchased commercially. CHO-K1 cells stably expressing G_α16_ were a gift from E. C. Butcher. HEK 293T, HeLa 229, Lenti-X, Plat-E and CHO cells were cultured in complete Dulbecco’s modified Eagle’s medium (cDMEM) medium consisting of DMEM (Gibco, catalogue no. 10566016) supplemented with 10% fetal bovine serum (FBS) (Gibco, catalogue no. 16140071), 2 mM l-glutamine (Gibco, catalogue no. 25030081) and penicillin–streptomycin (10 U ml^−1^ and 100 μg ml^−1^, respectively; Gibco, catalogue no. 15140122). Jurkat cells were cultured in complete RPMI 1640 (cRPMI) medium (Gibco, cat: 11875093) supplemented identically. All cells were cultured at 37 °C with 5% CO_2_. Cell lines were not tested for mycoplasma contamination.

### PBMC isolation and primary T cell culture

Human PBMCs were isolated by Ficoll-Paque PLUS (Cytiva, catalogue no. GE17-1440-02) density-gradient centrifugation, washed twice with PBS, and resuspended at 1 × 10^6^ cells per ml in cRPMI. PBMCs were activated with 1 μg ml^−1^ anti-CD3 (clone HIT3a, BioLegend, catalogue no. 300334) and 1 μg ml^−1^ anti-CD28 (clone CD28.2, BioLegend, catalogue no. 302944) or with Dynabeads Human T-Activator CD3/CD28 (Gibco, catalogue no. 11132D). After 2 days, cells were washed with PBS (Gibco, catalogue no. 10010023), activation beads (when used) were removed magnetically and cells were cultured in cRPMI supplemented with 100 U ml^−1^ recombinant human IL-2 (Teceleukin, catalogue no. Ro 23-6019). Medium was refreshed every 2 days.

### Human intestinal lymphocyte isolation

Frozen biopsies were thawed at 37 °C, washed in cRPMI and minced into small pieces. IELs were isolated by rotating tissues in Hank’s balanced salt solution (HBSS) containing 10% fetal calf serum, 5 mM EDTA and 5 mM dithiothreitol (Sigma, cat: 10197777001) at 37 °C for 40 min, then filtered through 100-μm cell strainers. Remaining tissue was digested in RPMI containing 1 mg ml^−1^ collagenase IV (Sigma, catalogue no. C5138) and 10 U ml^−1^ DNase I (Roche, catalogue no. 04716728001) at 37 °C for 1 h to isolate LP cells. Cells were filtered and centrifuged on a 40% Percoll gradient at 1,800 rpm, 4 °C for 10 min.

### Calcium flux assay

CHO-K1/G_α16_ cells stably transduced with WT or mutant human *Gpr15* were dissociated with 0.05% trypsin (Gibco, catalogue no. 25300054), washed with PBS and equilibrated in HBSS containing Ca^2+^ and Mg^2+^ (Gibco, catalogue no. 14025092) for 10 min. A total of 2 × 10^6^ cells were stained with 1 μM indo-1 acetoxymethyl ester (Invitrogen, catalogue no. I1223) and 1× PowerLoad Concentrate (Invitrogen, catalogue no. P10020) in HBSS for 30 min at room temperature in the dark. Cells were washed, resuspended in 500 μl HBSS and equilibrated for 5 min at room temperature. Calcium responses were analysed by flow cytometry following stimulation with 100 nM recombinant human GPR15L (PeproTech, catalogue no. 300-71). Baseline fluorescence was acquired for 30 s, followed by 1 min after GPR15L addition. Calcium levels were calculated as the ratio of indo-1 fluorescence in the bound (395 nm) to the unbound (525 nm) states.

### Transwell migration assay

Activated human T cells or Jurkat T cells stably transduced with WT or mutant human *Gpr15* were cultured overnight in RPMI containing 10% charcoal-stripped FBS (CS-FBS) (Gibco, catalogue no. A3382101). A total of 5 × 10^4^ cells in 25 μl medium were seeded onto the upper membrane of a 5-μm-pore (human T cells) or 8-μm-pore (Jurkat T cells) ChemoTx Disposable Chemotaxis System (NeuroProbe, catalogue no. 106-5, 106-8). Medium (30 μl) with or without 100 nM human GPR15L was added to the bottom chamber. After 3 h, cells from the upper membrane and bottom chamber were collected and counted by flow cytometry. Migration index = (number of cells in the bottom chamber/total number of cells in the transwell) with ligand/(number of cells in the bottom chamber/total number of cells in the transwell) with medium control.

### Mouse cell isolation

Spleen, MLN, thymus, small intestine and large intestine were collected from euthanized mice. Spleen, MLN and thymus were dissociated mechanically and filtered through 70-µm cell strainers in RPMI to generate single-cell suspensions. For intestinal cell isolation, visible fat and luminal contents were removed, intestines were opened longitudinally and Peyer’s patches were removed from the small intestine. Tissues were cut into 0.5–1-cm pieces and incubated at 37 °C with shaking (20 rpm) for 30 min in HBSS containing 5 mM EDTA and 1 mM dithiothreitol to isolate intestinal epithelial cells and IELs. Cell suspensions were filtered through 100-µm mesh filters, washed with RPMI containing 3% FBS, and centrifuged at 800*g* for 10 min. Intestinal epithelial cells and IELs were separated by centrifugation in 40% Percoll (GE Healthcare, catalogue no. GE17-0891-01) at 1,800 rpm for 10 min. Remaining intestinal tissue was minced and digested at 37 °C for 40 min in RPMI containing 0.1 mg ml^−1^ Liberase (Roche, catalogue no. 05401020001) and 10 U ml^−1^ DNase I. Digested tissue was filtered through a 70-µm cell strainer and centrifuged at 500*g* for 5 min. Intestinal LP lymphocytes were isolated by centrifugation in 40% Percoll at 1,800 rpm for 10 min.

### Mouse T cell culture

Mouse splenic T cells were activated with Dynabeads Mouse T-Activator CD3/CD28 (Gibco, catalogue no. 11452D) in cRPMI at 1 × 10^6^ cells ml^−1^. After 2 days, activation beads were removed magnetically and cells were cultured in cRPMI supplemented with 100 U ml^−1^ mouse IL-2 (BioLegend, catalogue no. 575404). Medium was refreshed every 2 days.

Mouse IELs isolated from intestine were stimulated with Dynabeads Mouse T-Activator CD3/CD28 in cRPMI supplemented with 10 U ml^−1^ IL-2, 100 ng ml^−1^ IL-15 (BioLegend, catalogue no. 566302), 100 U mL^−1^ IL-3 (BioLegend, catalogue no. 575504) and 200 U mL^−1^ IL-4 (BioLegend, catalogue no. 574304). After 2 days, activation beads were removed and cells were cultured in cRPMI supplemented with 10 U ml^−1^ IL-2 at 5 × 10^5^ cells ml^−1^. Medium was refreshed every 3–4 days.

### Mouse macrophage and CD8^+^ T cell co-culture

Bone marrow-derived macrophages were generated from femurs and tibias of WT C57BL/6 mice in cDMEM supplemented with 20 ng ml^−1^ macrophage colony-stimulating factor (M-CSF; GenScript, catalogue no. Z03275) for 7 days. Differentiated bone marrow-derived macrophages were detached with 0.05% trypsin (Gibco, catalogue no. 25300054), seeded into 24-well tissue culture-treated plates at 1 × 10^5^ cells per well, and stimulated with 100 ng ml^−1^ lipopolysaccharide (LPS; InvivoGen, catalogue no. tlrl-eblps) for 24 h. CD8α^+^ T cells isolated from WT C57BL/6 spleens (CD8^+^ T_Sp_) or GPR15^+^ CD8α^+^ T cells isolated from colonic IELs (CD8^+^ T_IGR_) were purified by flow cytometric sorting and stimulated with Dynabeads Mouse T-Activator CD3/CD28 in the same cytokine cocktail for IELs as described above. After 24 h, activation beads were removed.

Activated CD8^+^ T cells and macrophages were stained with CellTrace CFSE (Invitrogen, catalogue no. C34554) and CellTrace Violet (Invitrogen, catalogue no. C34571), respectively, at 37 °C for 20 min. After washing, CD8^+^ T cells were added to macrophages at ratios of 1:1, 1:2.5, 1:5, 1:10 and 1:20. Where indicated, 10 μg ml^−1^ anti-mouse TNF (BioLegend, catalogue no. 935903), anti-mouse IFNγ (BioLegend, catalogue no. 505708), anti-mouse FasL (BioLegend, cat: 106611), anti-mouse TWEAK (BioLegend, cat: 120009), anti-mouse TRAIL (BioLegend, catalogue no. 109315), anti-mouse H2 (BioLegend, catalogue no. 125502) or 100 nM concanamycin A (Sigma, catalogue no. C9705) were added. After 5 h of co-culture, CD8α^+^ T cells were removed and macrophages were stained with propidium iodide (BioLegend, catalogue no. 421301) for 5 min before imaging on a ZEISS Axio Vert.A1 TL/FL-LED inverted microscope using ZEN software (v.3.10, ZEISS). Images were analysed using ImageJ (v.2.14.0). Killing ratio was calculated as propidium iodide^+^ macrophages divided by total macrophages.

### Mouse intestinal organoid generation

Intestinal organoids were generated from WT C57BL/6 mice as described previously^50^. In brief, the intestines were opened longitudinally, washed with cold PBS, cut into 2-mm pieces, and incubated in Gentle Cell Dissociation Reagent (STEMCELL Technologies, catalogue no. 100-0485) at room temperature with shaking (20 rpm) for 15 min. Crypts were enriched using 70-μm cell strainers, centrifuged at 290*g* for 5 min, and suspended in a 1:1 mixture of IntestiCult Organoid Growth Medium (OGM; STEMCELL Technologies, catalogue no. 06005) and Matrigel Matrix (Corning, catalogue no. 354230). A total of 500 crypts in 50 μl suspension were seeded into pre-warmed 24-well plates. After polymerization, OGM was added and refreshed every 3–4 days. Organoids were maintained at 37 °C with 5% CO_2_ and passaged weekly.

### Mouse intestinal organoid and CD8^+^ T cell co-culture

Intestinal organoids were cultured for 2 days before co-culture with IELs. Organoids were released from Matrigel using Cell Recovery Solution (Corning, catalogue no. 354253) on ice for 30 min, washed and mixed with CD8^+^ T cells at a ratio of 100 organoids to 1 × 10^5^ cells in 200 μl cDMEM. After incubation at 37 °C for 30 min and centrifugation at 200*g* for 5 min, pellets were resuspended in a 1:1 mixture of Matrigel and OGM supplemented with 100 U ml^−1^ IL-2, 10 ng ml^−1^ IL-7 (BioLegend, catalogue no. 577804) and 10 ng ml^−1^ IL-15. A total of 30 μl suspension was plated into eight-well chambered coverslips (ibidi, catalogue no. 80824) and polymerized at 37 °C for 15 min before addition of 300 μl OGM containing the same cytokines.

Live-cell imaging was performed using a Leica Episcope inverted fluorescence microscope in a 37 °C, 5% CO_2_ chamber, with images acquired every 30 min. Confocal imaging was performed 2 days after co-culture using a Leica SP8 WLL FLIM microscope. Images were analysed using Imaris (v.10.2.0).

For flow cytometry analysis, 2 days after co-culture, medium containing free CD8^+^ T cells was collected. Organoids were released from Matrigel using Cell Recovery Solution on ice for 30 min, and supernatants containing free CD8^+^ T cells were combined with the collected medium. Free CD8^+^ cells were pelleted by centrifugation at 500*g* for 5 min. To isolate organoid-incorporated CD8^+^ T cells, organoid pellets were incubated in 5 mM EDTA/PBS on ice for 10 min, dissociated mechanically by vigorous pipetting, and centrifuged at 500*g* for 5 min. Free and organoid-incorporated CD8^+^ T cells were analysed subsequently by flow cytometry.

### Mouse colitis model

Experimental colitis was initiated in mice by administering 2.5% DSS (36,000–50,000 molecular weight, MP Biomedicals, catalogue no. 160110) in drinking water for 6 days, followed by normal water for another 6–7 days. Body weight was measured daily, and mice that lost more than 25% of initial body weight were euthanized according to a predetermined humane endpoint.

### Adoptive transfer studies

Competitive adoptive transfer of IELs was performed as described previously^19^. For IELp transfers, thymocytes from CD45-disparate mice were enriched by depletion of CD8^+^ and CD4^+^ cells with Untouched Mouse CD4 or CD8 Cells Kits (Invitrogen, catalogue nos. 11415D, 11417D), stained, and purified by flow cytometric sorting as described^19^. A total of 20,000 IELps were injected intravenously into each *Rag2*^*−/−*^*Il2rg*^*−/−*^ recipient mouse. For conventional CD8αβ^+^ splenocyte transfers, 5 × 10^5^ flow-sorted TCRβ^+^CD4^−^CD8α^+^CD8β^+^ splenocytes were injected intravenously into each recipient mouse. Before transfer, CD45.1^+^CD45.2^+^ cells from WT or *Gpr15*^*−/−*^ donor mice were mixed 1:1 with WT CD45.1^+^ cells. Recipient spleens, small intestines and large intestines were collected 2 weeks after transfer for flow cytometry analysis.

### Macrophage depletion

Macrophage depletion was performed as described previously using clodronate-containing liposomes^51^. In brief, 8-week-old WT or *Gpr15*^*−/−*^ mice received intraperitoneal injections of 200 μl clodronate liposomes (Fisher Scientific, catalogue no. NC0337390) every other day for 10 days. DSS colitis was induced with 2.5% DSS in drinking water beginning 4 days after initiation of clodronate treatment. After 6 days, DSS water was replaced with normal drinking water, and mice were monitored as described above. Control mice received 200 μl PBS liposomes (Fisher Scientific, catalogue no. NC2245953).

### Flow cytometry and sorting

PBMCs, T cells, or isolated mouse cells were washed with PBS and stained with LIVE/DEAD Fixable Yellow Stain (Invitrogen, catalogue no. L34959) diluted 1:1,000 in PBS for 30 min at room temperature. Cells were washed with fluorescence-activated cell sorting (FACS) buffer (PBS containing 2% FBS and 0.1% NaN_3_ (Sigma-Aldrich, catalogue no. S2002-500G)), blocked with Fc Receptor Blocking Solution (BioLegend, catalogue no. 422302 for human, catalogue no. 101320 for mouse) diluted 1:100 for 10 min on ice, and stained with fluorochrome-conjugated antibodies for 30 min on ice. For intracellular Foxp3 staining, cells were fixed, permeabilized and stained using the eBioscience Foxp3/Transcription Factor Staining Buffer Set (Invitrogen, catalogue no. 00-5523-00) following the manufacturer’s instructions. Cells were washed three times with FACS buffer before acquisition. Antibodies used are listed in Supplementary Table 2.

Flow cytometry data were acquired on FACSymphony A3 or LSRFortessa (BD Biosciences) with FACSDiva (v.9.1, v.9.0, BD Biosciences) and analysed with FlowJo (v.10.9.0, Tree Star). Dead cells and doublets were excluded by LIVE/DEAD staining and forward scatter height-versus-width gating, respectively. Cell sorting was performed using FACSAria or FACS Fusion (BD Biosciences). Gating strategies are shown in Supplementary Figs. 2–4.

### Immunoblotting

HEK 293T cells were transfected with WT or mutant human *Gpr15* using Lipofectamine 3000 (Invitrogen, catalogue no. L3000075). After 48 h, cells were collected, washed with cold PBS and lysed in buffer containing 1% NP-40, 50 mM Tris-Cl (pH 8.0) and 150 mM NaCl supplemented with Halt Protease Inhibitor Cocktail (Thermo Scientific, catalogue no. 78439) and Phosphatase Inhibitor Cocktail (Thermo Scientific, catalogue no. 78427). Lysates were incubated on ice for 30 min and clarified by centrifugation at 14,000 rpm, 4 °C for 20 min. Protein concentrations were determined using the BCA Protein Assay Kit (Thermo Scientific, catalogue no. 23225). Equal amounts of protein were mixed with NuPAGE LDS Sample Buffer (4×) (Invitrogen, catalogue no. NP0008) and Sample Reducing Agent (10×) (Invitrogen, catalogue no. NP0004), separated on 4–12% Bis-Tris gels (Invitrogen, catalogue no. NP0336BOX) and transferred to nitrocellulose membranes using the Power Blotter System (Invitrogen, catalogue no. PB0012). Membranes were blocked in 5% milk in TBST (0.05% Tween-20) for 1 h, incubated with primary antibodies overnight at 4 °C, followed by horseradish peroxidase (HRP)-conjugated secondary antibodies, developed with SuperSignal West Dura substrate (Thermo Scientific, catalogue no. 34076), and imaged using an Azure C500 imaging system (v.1.7.6.1202). Antibodies: mouse anti-human GPR15 (R&D Systems, clone 367902, catalogue no. MAB3654, 1:1,000), mouse anti-HSP90 (BioLegend, clone K3720A, catalogue no. 661802, 1:5,000), rabbit anti-β-Arrestin-1/2 (Cell Signaling, clone D24H9, catalogue no. 4674, 1:1,000), rabbit anti-14-3-3 (pan) (Cell Signaling, clone E9S9M, catalogue no. 95422, 1:1,000), rabbit anti-Flag (Sigma, clone SIG1-25, catalogue no. F2555, 1:1,000), goat anti-mouse IgG HRP (SouthernBiotech, catalogue no. 1030-05, 1:2,000), goat anti-rabbit IgG HRP (SouthernBiotech, catalogue no. 4050-05, 1:2,000) and mouse anti-rabbit IgG HRP (Rockland, clone eB182, catalogue no. 18-8816-31, 1:1,000). Source data for uncropped immunoblots are provided in Supplementary Fig. 1b,c.

### Co-immunoprecipitation

WT and mutant human *Gpr15* cDNAs were cloned into c-Flag pcDNA3 (Addgene, catalogue no. 20011). HEK 293T cells were transfected with WT or mutant human *Gpr15* using Lipofectamine 3000. After 48 h, cells were lysed in 1% NP-40 lysis buffer on ice for 30 min and clarified by centrifugation at 14,000 rpm, 4 °C for 20 min. Lysates were precleared with Dynabeads Protein G (Invitrogen, catalogue no. 10003D) for 30 min at 4 °C. A total of 5 μg rat anti-Flag antibody (BioLegend, clone L5, catalogue no. 637303) was coupled to 1.5 mg Dynabeads Protein G in PBS containing 0.1% Tween-20 at 4 °C for 1 h and incubated with cell lysates at 4 °C for 1 h. Immunoprecipitates were washed five times with lysis buffer, eluted in 2× LDS Sample Buffer for 5 min at 70 °C, and analysed by immunoblotting.

### Cell immunofluorescence staining and confocal microscopy

HeLa cells stably transduced with WT or mutant human *Gpr15* were plated onto glass coverslips in 24-well plates. After overnight culture, cells were fixed with methanol for 10 min at −20 °C, permeabilized with 0.1% Triton X-100 in PBS for 20 min then blocked in PBS containing 2% BSA and 2% goat serum (Sigma-Aldrich, catalogue no. G9023) for 1 h at room temperature. Coverslips were incubated with primary antibodies overnight at 4 °C, washed with PBS and stained with fluorochrome-conjugated secondary antibodies and Hoechst 33342 (2 μg ml^−1^; Thermo Fisher, catalogue no. H3570) for 1 h at room temperature in the dark. Coverslips were mounted with ProLong Diamond Antifade Mountant (Invitrogen, catalogue no. P36961). Images were acquired using a Leica SP8 WLL FLIM confocal microscope and analysed with Imaris (v.10.2.0). Antibodies: mouse anti-GPR15 (R&D Systems, clone 367902, catalogue no. MAB3654, 1:100), rabbit anti-calnexin (Cell Signaling, clone C5C9, catalogue no. 2679, 1:200), rabbit anti-giantin (BioLegend, catalogue no. 909701, 1:1,000), Alexa Fluor Plus 594 goat anti-rabbit IgG (H+L) (Invitrogen, catalogue no. A32740, 1:1,000) and Alexa Fluor 488 donkey anti-mouse IgG (H+L) (Invitrogen, catalogue no. A21202, 1:1,000).

### Immunohistochemistry and multiplex immunofluorescence staining

Mouse intestinal tissues were fixed in 10% neutral buffered formalin, transferred to 70% ethanol, paraffin-embedded and sectioned at 5 μm. Human intestinal tissues and tissue microarrays from healthy donors and patients with sporadic IBD were obtained from US Biomax (catalogue nos. SIN241, SM2081, BC05002c, CO245 and CO246) and Zyagen (catalogue nos. HP-307, HP-308, HP-309 and HP-311). H&E staining was performed by Histoserv Inc.

For multiplex immunofluorescence staining, deparaffinization and staining were performed using a Leica BOND Rx autostainer (Leica Biosystems). Heat-induced epitope retrieval was carried out at 95 °C using citrate-based ER1 (Leica Biosystems, catalogue no. AR9961) or Tris-EDTA-based ER2 (Leica Biosystems, catalogue no. AR9640). Tyramide signal amplification Opal technology (Akoya Biosciences) was used for multiplex staining. Primary antibodies were incubated for 60 min, followed by HRP-conjugated secondary antibodies for 30 min and fluorescent signal amplification for each target. Opal fluorophores (Akoya Biosciences; Opal 480, 520, 570, 620, 690 and 780) were used at 1:150, except Opal 780, which was used at 1:50 and combined with Tyramide signal amplification-digoxigenin at 1:100. Slides were coverslipped using a Leica CV5030 automated glass coverslipper (Leica Biosystems). Whole-slide images were acquired at ×40 or ×20 magnification using a PhenoImager (Akoya Biosciences) and analysed with QuPath software (v.0.5.1). Antibodies: rabbit anti-CD4 (Abcam, clone EPR6855, catalogue no. ab133616, 1:1,000), rabbit anti-FOXP3 (Thermo Fisher, clone SP97, catalogue no. MA5-16365, 1:200), rabbit anti-CD8 (Abcam, clone EPR10640(2), catalogue no. ab189926, 1:1,000), mouse anti-CD3 (Leica, clone LN10, catalogue no. PA0553, ready-to-use), rabbit anti-GPR15 (Sigma, catalogue no. HPA013775, 1:50), rat anti-F4/80 antibody (Thermo Fisher, clone BM8, catalogue no. 14-4801-82, 1:150), rabbit anti-CD206 (Abcam, catalogue no. ab64693, 1:1,000) and rabbit anti-NOS2 (Abcam, catalogue no. ab15323, 1:100).

For iterative bleaching extends multiplexity (IBEX) staining, sections were deparaffinized, subjected to antigen retrieval in Borg Decloaker RTU (pH 9; Biocare Medical, catalogue no. BD1000G1) at 110 °C for 15 min, permeabilized with 0.3% Triton X-100 and 1% BSA in PBS, stained with primary antibodies and secondary antibodies, blocked with 10% rabbit or mouse serum (Invitrogen, catalogue nos. 31883, 31881) and stained with fluorochrome-conjugated antibodies. Fast staining was performed using a PELCO BioWave Pro-36500-230 microwave equipped with a PELCO SteadyTemp Pro-50062 thermoelectric recirculating chiller (Ted Pella) as reported previously^52^. Hoechst 33342 was included in all panels for image alignment. Slides were mounted with Fluoromount-G (Thermo Fisher, catalogue no. 00-4958-02) and imaged using a Leica SP8 inverted confocal microscope equipped with a ×40/1.3 numerical aperture oil objective. Spectral spillover was corrected by linear unmixing in LAS X software (Leica, v.3.5.7) and stage positions were recorded for iterative imaging. Fluorophores were bleached with 1 mg ml^−1^ lithium borohydride between staining cycles, and subsequent images were acquired using the recorded stage positions. Alignment of IBEX panels was performed using SimpleITK with Hoechst staining as the common registration channel^53^. Histocytometry analysis was performed as described previously^54^. In brief, imaging datasets were segmented on the basis of nuclear staining, and average voxel intensities were extracted in Imaris. Channel statistics were exported and analysed using FlowJo (v.10.9.0, Tree Star). Antibodies: rabbit anti-CD11c AF647 (Abcam, clone EP1347Y, catalogue no. ab275338, 1:50), mouse anti-CD68 iFluor 594 (Caprico Biotechnologies, clone KP-1, catalogue no. 164136, 1:50), mouse anti-HLA-DR AF532 (Novus Biologicals, clone TAL1B5, catalogue no. NB600-989AF532, 1:500), rabbit anti-CD3 (Abcam, clone SP7, catalogue no. ab16669, 1:50), goat anti-rabbit IgG (H+L) AF532 (Thermo Fisher, catalogue no. A11009, 1:250) and Hoechst 33342 (Biotium, catalogue no. 40046, 1:5,000). Histocytometry gating strategies are provided in Supplementary Fig. 5.

### Lentiviral and retroviral production and infection

WT and mutant human or mouse *Gpr15* cDNAs were cloned into *pLV-EF1a-IRES-Puro* (Addgene, catalogue no. 85132), *pLV-EF1a-IRES-eGFP* or *pMSCV-IRES-mCherry* (Addgene, catalogue no. 52114) vectors using Takara In-Fusion Snap Assembly Master Mix (Takara, catalogue no. 638952). Lentivirus and retrovirus were produced in Lenti-X 293T or Platinum-E cells, respectively, by co-transfection of viral backbone plasmids with psPAX2 and pMD2.G (Lentivirus; Addgene, catalogue nos. 12260, 12259) or pCL-Eco (Retrovirus; Addgene, catalogue no. 12371) using Lipofectamine 3000. Viral supernatants were collected 48 h after transfection, clarified by centrifugation at 500*g* for 15 min at 4 °C and concentrated using Lenti-X Concentrator (Takara, catalogue no. 631232) or Retro-X Concentrator (Takara, catalogue no. 631455), resuspended in Opti-MEM and stored at −80 °C. Cells were spin-infected at 2,000 rpm for 90 min at 32 °C in the presence of 10 μg ml^−1^ polybrene (Sigma-Aldrich, catalogue no. TR-1003-G). Primary T cells were transduced 2 days after CD3/CD28 bead activation in cRPMI containing IL-2. Transduced cells were selected with 4 μg ml^−1^ puromycin (Thermo Fisher, catalogue no. A1113803) or purified as mCherry^+^ cells by flow cytometric sorting 72 h after infection.

### scRNA-seq analysis of the human Gut Cell Atlas

Human intestinal scRNA-seq data were obtained from the Gut Cell Atlas and analysed using Scanpy (v.1.9.1) in Python^27^. Data was preprocessed, quality controlled and annotated as described previously^27^. Cells expressing fewer than 500 or more than 7,000 genes and genes detected in fewer than 20 cells were excluded. In total, 153,961 cells from the intestines of healthy adults and children were analysed. Gene expression values were normalized to 10,000 counts per cell and log-transformed. Batch correction was performed with bbknn (v.1.5.1), followed by principal component analysis (PCA), Leiden clustering and UMAP visualization.

### CITE-seq experimental methods

#### Single-cell CITE-seq processing

Human intestinal biopsies were obtained at the Gazi University Faculty of Medicine and stored at Hacettepe University (two healthy control individuals: HC1, 59-year-old male; HC2, 48-year-old female; and one patient: Pt 1.1, 18-year-old female) and at Boston Children’s Hospital (one patient: Pt 3.1, 17-year-old male). Control individuals were healthy relatives of patients with gastrointestinal disease (not relatives of patients in our study). Endoscopic biopsies were collected from eight intestinal segments: duodenum, terminal ileum, caecum, ascending colon, transverse colon, descending colon, sigmoid colon and rectum. Samples were immersed immediately in cold MACS Tissue Storage Solution (Miltenyi Biotec, catalogue no. 130-100-008) on ice and cryopreserved subsequently in CryoStor CS10 medium (STEMCELL Technologies, catalogue no. 100-1061). Tissue dissociation was performed within 2 h of retrieval. IELs and LP cells were isolated, and CD45^+^ lymphocytes were sorted and stained with Totalseq-C Hashtag antibodies (BioLegend) at 4 °C for 30 min, washed three times with CITE-seq staining buffer (1% BSA in PBS), pooled and then stained with a cocktail of TotalSeq-C Human Panel antibodies (BioLegend, catalogue no. 399905) (Supplementary Table 3) at 4 °C for 30 min.

Mouse CD8α^+^ large intestinal IELs and CD8αα^+^ splenocytes were sorted from WT or *Gpr15* mice, stained with Totalseq-A Hashtag antibodies (BioLegend) at 4 °C for 30 min, washed three times with CITE-seq staining buffer, pooled and then stained with a cocktail of TotalSeq-A mouse-selected panel antibodies (BioLegend), including 17 surface proteins and four isotype controls (Supplementary Table 3) at 4 °C for 30 min*.*

#### Single-cell CITE-seq library construction and sequencing

The pooled samples were washed, counted, resuspended in PBS and partitioned into single-cell Gel Beads-in-Emulsion (GEMs) together with reverse transcription reagents using 10x 5′ Chromium Single Cell Immune Profiling Next GEM v.1.1 (human) or 10x 3′ Chromium Single Cell Next GEM v.3.1 (mouse) chemistry (10x Genomics). Reverse transcription was conducted in a Veriti Thermal Cycler (Thermo Fisher Scientific). Single-cell gene expression, cell-surface protein tag and human TCR libraries were prepared following the protocols provided in the 10x Genomics user guides (https://www.10xgenomics.com/resources/user-guides/) and by BioLegend (https://biolegend.com/en-us/protocols/totalseq-a-dual-index-protocol). All libraries were quality controlled using a Bioanalyzer (Agilent), quantified using Qubit Fluorometric Quantitation (Thermo Fisher Scientific), pooled and sequenced on an Illumina NovaSeq X Plus 10B platform (human) or a BGI DNBSEQ-T7 PE100 platform (mouse) using the following sequencing parameters: Read1-28-cycle, i7-10-cycle, i5-10-cycle, Read2-90-cycle.

### CITE-seq data processing and statistical analyses

#### Single-cell sample preprocessing

Single-cell sequencing data were processed using 10x Genomics pipelines and analysed in Seurat (v.5.0.3) in R (v.4.4.0). Raw BCL files were demultiplexed and converted to FASTQ format. Human reads were aligned to the GRCh38 reference genome (2024-A) using CellRanger multi (v.9.0), whereas mouse reads were aligned to the mm10 reference genome (2020-A) using CellRanger count (v.6.1.2). Count matrices were generated using CellRanger, and hashtag oligonucleotide reads were used for sample demultiplexing with HTODemux in Seurat.

Filtering of low-quality cells and potential doublets was based on the number of detected genes, read counts and the percentage of mitochondrial reads. Human cells were retained if the numbers of detected genes and reads were within three median absolute deviations of the median and mitochondrial read percentages were fewer than three median absolute deviations above the median, whereas mouse cells with fewer than 200 detected genes or more than 5% mitochondrial reads were excluded. RNA data were normalized using Seurat’s SCTransform, which applies a regularized negative binomial regression model to correct for sequencing depth and technical variability.

Pt 1.1 and Pt 3.1 samples were merged while treating each library capture as a separate batch. Cell type annotation was performed using SingleR (v.2.6.0) with the human gut cell atlas (gut_healthyPedAdult) from ref. ^55^. CD8^+^ T cells were then extracted based on these annotations. For comparison, CD8^+^ T cells were integrated with cells from the healthy adult/paediatric gut atlas^27^ after excluding appendix, MLN, jejunum and proximal and middle ileum (ILE1 and ILE2) tissues to match the tissues represented in our samples. Integration was performed using the Seurat CCA workflow with 30 principal components, and clustering was conducted at a resolution of 0.4.

A total of 6,673 human intestinal CD8^+^ cells were analysed (16 control individuals: 4,220 cells; Pt 1.1: 1,719 cells; Pt 3.1: 734 cells). For the mouse dataset, 31,585 cells were sequenced (WT spleen: 7,691 cells; *Gpr15* spleen: 9,239 cells; WT colon: 11,553 cells; *Gpr15* colon: 3,102 cells).

#### Dimensionality reduction and clustering

Seurat objects were subjected to PCA, and principal components were selected based on the ElbowPlot. Cell clustering was performed using the Louvain algorithm implemented in the FindNeighbors and FindClusters functions, and clusters were visualized using UMAP with the RunUMAP function.

#### Differential expression and functional analysis

Differential gene expression analysis was performed using the Seurat FindMarkers function with the Wilcoxon rank-sum test. Differentially expressed genes were visualized using FeaturePlot, VlnPlot, DotPlot and analysed functionally using SIGNAL^56^ and DAVID^57^. Heatmaps, GSEA and volcano plots were generated using pheatmap (v.1.0.13), fgsea (v.1.36.2) and ggplot2 (v.4.0.2), respectively.

### Single-cell TCR RNA-seq analysis

Single-cell TCR V(D)J data were aligned to the human GRCh38 reference (CellRanger reference v.7.1.0) using the 10x Genomics CellRanger multi pipeline (v.9.0). Filtered contig data were processed using scRepertoire (v.2.4.0) in R following published vignettes^58,59^, and clonotype annotations were integrated into the scRNA-seq object as metadata. CD8^+^ T cells from HC samples (HC1 and HC2) were extracted and analysed in Python using Scanpy (v.1.9.1). Dimensionality reduction and clustering were performed using PCA (30 principal components), *k*-nearest neighbour graph construction, UMAP embedding and Leiden clustering (resolution = 0.5; random_state = 15). A total of 2,764 healthy human intestinal CD8^+^ cells were analysed (HC1: 1,088 cells; HC2: 1,676 cells).

Cells with captured TCRs were identified based on CTnt annotations, and paired αβ TCR clonotypes were defined from CTstrict entries containing both TRA and TRB chains. Clonotypes were categorized as small (1 × 10^−4^ < *X* ≤ 0.001), medium (0.001 < *X* ≤ 0.01) and large (0.01 < *X* ≤ 0.1) based on clonalProportion values (*X*) and visualized on UMAP embeddings. The number of clonotypes in each category was quantified per Leiden cluster and visualized as stacked bar plots. Clonal overlap between donors and samples was assessed using contingency tables and clustered heatmaps. Clonal diversity across Leiden clusters was evaluated using the Shannon index, Chao1 richness, Gini index and the number of unique clonotypes, and visualized using box plots with donor-level overlays.

### Exome and whole-genome sequencing and analysis

Genomic DNA was isolated from PBMCs or saliva of probands, parents and siblings as indicated in each pedigree. WES was performed using the IDT xGen Exome Capture Kit (IDT) or SureSelect Human All Exon 50 Mb Kit (Agilent Technologies) followed by DNA sequencing on an Illumina HiSeq 2500. Whole-genome sequencing (WGS) was performed using the Standard Human WGS from Broad Institute. WES produced coverage of around 50–100× and WGS produced 60× and 30× coverage for probands and family members, respectively. Short reads were aligned to the hg19 human reference genome using Burrows-Wheeler Aligner (v.0.7.17). Variants were called using the Genome Analysis ToolKit (v.3.5), annotated by their functional impact on encoded proteins, and prioritized based on computational deleteriousness prediction, inheritance model, population frequency from dbSNP (v.137), gnomAD and Yale database (2,500 European samples, including 1,894 Turkish exomes) and so on. All possible inheritance modes and variants including de novo, autosomal recessive and hemizygous were evaluated. Genes with rare homozygous or compound heterozygous variants and heterozygous variants with potential dominant or haploinsufficient effects were prioritized.

### Statistical analyses

Sample size was not predetermined by statistical methods. No data were excluded. Investigators were not blinded to genotype because group allocation was based on genotype. For histological image analysis, genotypes were blinded to ensure unbiased quantification. Except for sequencing data and population-based analyses, we used GraphPad Prism (v.10.2.0) to perform two-sided Mann–Whitney *U*-tests, unpaired two-sided *t*-test, Kruskal–Wallis tests with Dunn’s multiple-comparisons test, one-way ANOVA with Tukey’s or Dunnett’s multiple-comparisons tests, or two-way ANOVA with Geisser–Greenhouse correction, as indicated in the figure legends. *P* values < 0.05 were considered significant, whereas *P* values ≥ 0.05 were considered non-significant.

### Reporting summary

Further information on research design is available in the Nature Portfolio Reporting Summary linked to this article.

## Online content

Any methods, additional references, Nature Portfolio reporting summaries, source data, extended data, supplementary information, acknowledgements, peer review information; details of author contributions and competing interests; and statements of data and code availability are available at 10.1038/s41586-026-10749-4.

## Supplementary information

Supplementary Information Supplementary Notes containing patient clinical summaries and Supplementary Methods containing detailed methods for rare variant association and machine learning analyses of *GPR15* in IBD: rare variant association data analysis in IBD cohorts; machine learning analyses in the AoU population.

Reporting Summary

Supplementary Figures Supplementary Figs. 1–5.

Supplementary Table 1 Clinical and laboratory findings of GPR15 patients.

Supplementary Table 2 Flow cytometry antibody list.

Supplementary Table 3 Mouse TotalSeq-A and human TotalSeq-C antibody lists.

Supplementary Videos Supplementary Videos 1–6 showing *Gpr15*^*−/−*^ CD8^+^ T cells transduced with mCherry-tagged WT mouse GPR15 or mutant constructs were co-cultured with mouse intestinal organoids for 48 h. Transduced cells are shown in red. Supplementary Video 1 shows WT GPR15, Video 2 shows D306N, Video 3 shows Q281X, Video 4 shows Y215X, Video 5 shows Y132S and Video 6 shows EV.

## Source data

Source Data Fig. 1

Source Data Fig. 2

Source Data Fig. 3

Source Data Fig. 4

Source Data Fig. 5

Source Data Extended Data Fig. 1

Source Data Extended Data Fig. 2

Source Data Extended Data Fig. 3

Source Data Extended Data Fig. 4

Source Data Extended Data Fig. 6

Source Data Extended Data Fig. 7

Source Data Extended Data Fig. 9

Source Data Extended Data Fig. 10

## Data Availability

Human WES and WGS data from the four families evaluated at NIH have been deposited in dbGaP (phs003950.v1.p1). Raw WES data for the Turkish/Qatari and Iranian cohorts are available under controlled access owing to ethical and legal restrictions related to patient privacy and the terms of informed consent. Requests for access should be directed to Bernice Lo (blo@sidra.org) for the Turkish and Qatari cohorts and Hassan Abolhassani (seyed.abolhassani@ukbonn.de) for the Iranian cohort and are subject to institutional ethics approval and data use agreements. The requests for access will be evaluated by the Data Access Committee, and applicants can expect an initial response in about 2–4 weeks. Human and mouse scRNA-seq datasets generated in this study have been deposited in Gene Expression Omnibus (GEO) (GSE314446). Raw human scRNA-seq reads are available through dbGaP (phs003950.v1.p1). scRNA-seq data from healthy adult and paediatric guts, ulcerative colitis patients, Crohn’s disease patients and blood KIR^+^CD8^+^ T cells were obtained from the Gut Cell Atlas (https://www.gutcellatlas.org/; ArrayExpress: E-MTAB-9543, E-MTAB-9536, E-MTAB-9532, E-MTAB-9533 and E-MTAB-10386)^27^, GEO (GSE125527)^37^, the Broad Institute Single Cell Portal (https://singlecell.broadinstitute.org/single_cell/, SCP1884; Broad DUOS: DUOS-000146 CD_Atlas_2021_GIDER; DUOS-000145 CD_Atlas_2021_PRISM)^38^ and GEO (GSE193770)^16^. AoU datasets were obtained from the AoU Research Program (Controlled Tier Dataset v.8, https://www.researchallofus.org/)^26^. An exception to the Data and Statistics Dissemination Policy was granted by the AoU Resource Access Board (Record number: 160). UK Biobank analyses were performed through Genebass (https://app.genebass.org/)^25^. Population allele frequencies, variant deleteriousness (CADD) and protein structural predictions were obtained from gnomAD (https://gnomad.broadinstitute.org/)^60^, CADD (https://cadd.gs.washington.edu/)^61^ and AlphaFold (https://alphafold.ebi.ac.uk/)^62^. Human GPR15 schematics shown in Fig. 1c and Extended Data Fig. 4c were adaptively created using PROTTER (https://protter.ethz.ch/start/)^63^. Source data are provided with this paper.

## Code availability

Data were analysed using publicly available software and standard pipelines, as described in Methods. No new software or code package was developed for this study.
