# Supplementary material for: GPR15-guided CD8+ T regulatory cells control intestinal inflammation
Source: Nature. 2026 Jun 8;656(8129):1003–12. doi: 10.1038/s41586-026-10749-4 (PMC13518229; doi:10.1038/s41586-026-10749-4)
Supplement: Supplementary file 1 [file 41586_2026_10749_MOESM1_ESM.pdf]

nature portfolio

https://doi.org/10.1038/s41586-026-10749-4

Supplementary information

GPR15-guided CD8$^+$ T regulatory cells control intestinal inflammation

In the format provided by the
authors and unedited

Supplementary Notes

Patient Clinical Summaries

Family 1

Patient 1.1 (proband) is an 18-year-old female born to non-consanguineous parents who developed chronic abdominal pain, diarrhea, and weight loss at the age of 7 years. Abdominal ultrasound was unremarkable. An esophagogastroduodenoscopy (EGD) showed multiple duodenal ulcers and scalloping of duodenal folds. Histopathology revealed *Helicobacter pylori*-negative chronic gastritis, Marsh type 3 duodenal lesions, and eosinophilic enteritis. She had a positive endomysial antibody (EMA) and was found to be homozygous for HLA-DQ2, which was suggestive of celiac disease. She was started on a gluten-free diet and enteral nutrition. Although EMA became negative on a gluten-free diet, she had to be hospitalized for management of weight loss and malnutrition at the age of 8 years. She was found to have hypoalbuminemia, anemia, leukocytosis, thrombocytosis, elevated C-reactive protein, elevated fecal calprotectin, and a positive guaiac fecal occult blood test. Magnetic resonance enterography (MRE) showed increasing wall thickness in the descending colon and rectum. She had a colonoscopy and biopsies that showed eosinophilic ileitis and chronic inflammatory changes with necrotic exudates in the colonic mucosa. Zinc levels in the hair were within normal limits. Antinuclear antibodies (ANA) were positive (nucleolar pattern). She also had a positive anti-dsDNA. Thyroid function tests were normal. No thyroid autoantibodies were detected. Serum IgG subclasses, IgA, and IgM levels were within normal limits. Lymphocyte immunophenotyping in peripheral blood showed elevated $CD4^{-}CD8^{-}$ T cells, while γ/δ T cells were in the upper limit of normal. Allergy testing in blood showed no IgE sensitivity to any of the aeroallergens tested. She was started on oral azathioprine, methylprednisolone, a proton pump inhibitor, and montelukast for management of autoimmune/eosinophilic enteropathy. She also received iron, calcium and vitamin D supplementation. She was noted to have active hepatitis B virus infection while on immunosuppressants and was treated with lamivudine. Targeted genetic testing for known inborn errors of immunity (IEI) was unrevealing. A follow-up EGD done 7 months later showed multiple gastric ulcers, nodularity, and erosive ulcers in the bulb and second part of the duodenum. Histopathology showed that the small bowel (duodenum) mucosa had villous flattening and intraepithelial lymphocytosis. No significant abnormalities were observed in the gastric mucosa (corpus) or esophagus. Colonoscopy with biopsies revealed ileitis with architectural distortion and chronic and acute inflammation, including eosinophil infiltration. The cecum also showed chronic and acute inflammation with eosinophil infiltration and detached fragments of fibrinopurulent material. Colon biopsy showed ulcer and granulation tissue and fibrinopurulent material. The hepatic flexure and transverse colon had ulcers and granulation tissue. Descending colon, sigmoid colon, and rectum displayed acute and chronic inflammation and detached

fibrinopurulent material. Over the following years, her weight and height remained 2 standard deviations below the mean. She continued to be symptomatic, and infliximab was added to azathioprine at the age of 10 years. She was also switched from lamivudine to tenofovir due to the emergence of the tyrosine-methionine-aspartate-aspartate (YMDD) mutation in the Hepatitis B virus (HBV) polymerase gene and increasing HBV viremia. An EGD done 9 to 10 months later, while on azathioprine and infliximab, showed small bowel (duodenal bulb) mucosa with villous shortening, acute and chronic inflammation, chronic gastritis (antrum), and ulcerated gastric mucosa (corpus). Azathioprine and infliximab were stopped, and she was subsequently treated with a polymeric TGF-β2-enriched formula (Modulen). Adalimumab was added to this therapeutic regimen 4 weeks later. She continued to be symptomatic and an EGD with biopsies done 3 months later again showed small bowel (duodenum) villous flattening with acute and chronic inflammation. Histopathology of the gastric mucosa revealed chronic inflammation with detached fragments of fibrinopurulent material. Esophageal biopsy showed squamous mucosa with no significant changes. Adalimumab was stopped, and she was treated with a long taper of systemic corticosteroids (prednisolone) and oral budesonide with interval improvement. At age 11, she was started on vedolizumab while continuing low-dose oral prednisolone and oral budesonide. Seven months later, methotrexate was added to this regimen, and she was started on partial enteric nutrition. However, prednisolone was stopped a year later upon the emergence of severe osteoporosis. Supplementary enteric nutrition was also stopped. Budesonide was stopped at the age of 14 years. An EGD with biopsies done 2 years later on vedolizumab combined with methotrexate showed lobulated duodenal mucosa and hyperemic, nodular and patchy atrophic gastric mucosa (antrum, peripyloric area, corpus, and fundus). Histopathology showed intestinal metaplasia and chronic gastritis (antrum and corpus). Colonoscopy and biopsy were normal. At the age of 16, methotrexate was stopped, and a steroid inhaler for asthma was started 10 months later. Eosinophils in the blood and serum IgE were elevated during asthma exacerbations. She currently continues treatment on vedolizumab.

Family 2

Patient 2.1 (proband). A 23-year-old male born to non-consanguineous parents presented with abdominal pain and weight loss at the age of 10 years. Laboratory results were remarkable for iron deficiency and hypoalbuminemia. An EGD showed a large single duodenal ulcer. Colonoscopy revealed a cecal ulcer and two small ulcers in the terminal ileum. Histopathology showed chronic duodenitis with partial villous atrophy and inflammation in the terminal ileum with no evidence of granulomas. He also had multiple non-necrotizing granulomas, cryptitis, and crypt abscesses in the colon. The patient was diagnosed with Crohn's disease and was started on an oral prednisone taper and azathioprine. A year later, his gastrointestinal

symptoms recurred, and he was started on adalimumab with complete resolution of his gastrointestinal symptoms. However, he then developed abdominal pain, weight loss, and hematemesis at the age of 14 years. An EGD showed duodenal ulcers. Azathioprine was stopped and he was started on methotrexate while continuing adalimumab. Repeat EGD and colonoscopy performed a year later were unremarkable except for mild narrowing of the terminal ileum. Methotrexate was stopped and the patient subsequently developed severe abdominal pain at the age of 15 years. He was hospitalized and his symptoms improved with systemic corticosteroids. A year later, he developed abdominal pain and vomiting. An EGD showed multiple duodenal ulcers. Colonoscopy revealed a few ulcerations at the terminal ileum. Since diagnosis, the patient has had recurrent annual relapses. The patient's symptoms are currently well controlled on adalimumab monotherapy.

Patient 2.2 (affected heterozygous mother). A 48-year-old female presented with abdominal pain at the age of 23 years. A barium study showed narrowing of the terminal ileum. EGD and colonoscopy revealed Crohn's disease. She was treated with azathioprine and oral mesalazine. She also required intermittent courses of oral budesonide for control of her gastrointestinal symptoms. A capsule endoscopy done 8 years later showed no abnormality. She has had 3 acute exacerbations of her inflammatory bowel disease (IBD) symptoms over the last 20 years. She stopped all IBD medications in 2020 and has been asymptomatic since then.

Patient 2.3 (affected heterozygous sister). A 24-year-old female born to non-consanguineous parents presented with abdominal pain, weight loss, poor appetite, and diarrhea at the age of 8 years. Laboratory results revealed iron deficiency and hypoalbuminemia. An EGD was unremarkable. Colonoscopy revealed cecal deformity. Histopathology showed chronic duodenitis, granulomas in the stomach and cecum, and acute inflammation in the colon. She was diagnosed with Crohn's disease and was treated with a short course of oral prednisone followed by sulfasalazine and azathioprine. At the age of 12 years, adalimumab was added for worsening abdominal pain, poor weight gain, and iron deficiency. She continued to have abdominal pain and poor weight gain while on azathioprine and adalimumab. A repeat EGD done at the age of 14 years showed mild chronic gastritis. Colonoscopy was normal. The patient failed to gain any weight over the following 14 months. At that juncture, adalimumab was stopped and the patient was started on infliximab. She was maintained on infliximab infusions every 8 weeks. EGD and colonoscopy done at the age of 18 years were both normal. The patient is currently asymptomatic.

Patient 2.4 (affected heterozygous brother). A 12-year-old male born to non-consanguineous parents presented at the age of 11 years with perianal fistula, abdominal pain, and poor appetite. Laboratory tests showed elevated CRP and stool calprotectin. Endoscopy revealed a small antral ulcer, and colonoscopy demonstrated scattered ulcers in the cecum with an edematous ileocecal valve and transverse colon mucosa;

the remainder of the colon was unremarkable. Histopathology showed mild focal active duodenitis, mild chronic gastritis without *Helicobacter pylori*, focal active inflammation in the cecum, and no granulomas or chronic changes in the colon. MRI demonstrated moderate terminal and distal ileal inflammation with mural edema and mild luminal narrowing, and bilateral intersphincteric fistulas without abscess or supralevator extension. He was diagnosed with moderately active Crohn's disease. At presentation, the patient weighed 26 kg (below the 3rd percentile) and received four doses of infliximab (10 mg/kg every six weeks) along with three iron infusions.

Family 3

Patient 3.1 (proband). A 12-year-old Hispanic male presented with weight loss, loose stools, and rectal bleeding. He had a negative workup for infectious gastroenteritis and was noted to have anemia, elevated C-reactive protein, erythrocyte sedimentation rate, and fecal calprotectin. Lymphocyte immunophenotyping in peripheral blood showed elevated $CD4^{-}CD8^{-}$ T cells, while the other immune cells were within the normal range. Due to the COVID-19-related public health emergency, he was treated empirically with extended-release oral budesonide, and oral and rectal mesalamine for management of presumed IBD. A few weeks later, he presented with abdominal pain and was hospitalized for further evaluation and management. Laboratory results showed elevated serum lipase and abdominal ultrasound revealed pancreatic head swelling. EGD and colonoscopy showed patchy mildly erythematous mucosa without bleeding in the stomach. The esophagus and duodenum appeared normal. Localized moderate erythema, friability, loss of vascularity, and aphthous ulceration were noted in the most distal rectum. The remainder of the colon had a normal appearance. Histopathology showed *Helicobacter pylori*-negative chronic inactive gastritis and chronic mildly active colitis in the rectum. The latter is characterized by modest basal lymphoplasmacytosis and basal crypt architectural distortion. No abnormal findings in the esophagus, duodenum, terminal ileum, cecum, right/transverse/descending colon, or sigmoid. His hospital course was complicated by the recurrence of parotid gland swelling, which had been intermittent since 10 years of age. A parotid gland biopsy showed periductal inflammation with negative IgG4 staining. He was discharged on rectal mesalamine enemas and budesonide. There was a good clinical response, but he could not wean budesonide so infliximab was started 4 months later. His gastrointestinal symptoms improved, but he subsequently developed persistent neutropenia, so after 6 months, infliximab was discontinued and vedolizumab was started. He experienced a flare of his gastrointestinal symptoms 2 months after starting vedolizumab and required treatment with high-dose oral prednisolone, which was tapered and discontinued 3 months later. Five months later, he had symptomatic relapse due to C. difficile infection following cefdinir treatment for sinusitis. This was treated with vancomycin. EGD and colonoscopy performed after 1 year of vedolizumab

treatment revealed a normal-appearing esophagus, stomach and duodenum. Ileocolonoscopy was also normal apart from mild periappendiceal inflammation improved from diagnosis (Mayo 0). On histologic examination, gastritis had resolved. There was focal mild colitis in the cecum and focal moderately active colitis in the descending colon. The ileum, ascending and transverse colon and rectum were all normal. He has continued on vedolizumab 300 mg intravenously every 4 weeks. Six months after his most recent endoscopy, breakthrough symptoms and elevated fecal calprotectin in the setting of inconsistent mesalamine administration were treated successfully with oral prednisone and he also received intravenous iron infusions. At his most recent follow-up (five years after diagnosis), he had subsequently successfully weaned mesalamine and was in remission on vedolizumab monotherapy 300 mg intravenously every 4 weeks. His medical history is also significant for focal, presumably right centro-temporal epilepsy presenting with left face tonic seizures followed by generalized tonic-clonic seizures diagnosed at the age of 3 years. At the time of ulcerative colitis diagnosis, he was receiving valproic acid. Serial EEGs have been normal since 10 years of age and all antiepileptics were discontinued and he has remained seizure-free since 14 years of age. He has had transient elevations in serum and salivary amylase since the age of 7 years. He has had intermittent hematuria, first noted at the age of 6 years. This has been attributed to IgA nephropathy; however, he has not had a biopsy to confirm the diagnosis. Renal ultrasound, serum calcium, complement levels and ANA have been normal. Tissue transglutaminase IgA was 0.8 (upper limit of normal 6.9) at 13 years of age.

Family 4

Patient 4.1 (proband). A 4-year-old white male born to consanguineous parents presented with protein-losing enteropathy (PLE) and intestinal lymphangiectasia (IL), with symptom onset at the age of 2 years.

Initial laboratory evaluation demonstrated hypoproteinemia, hypoalbuminemia, and a mildly elevated CRP. Endoscopy showed hyperemic antral mucosa and white plaques over the duodenal bulb and postbulbar area, while histopathology confirmed intestinal lymphangiectasia with nonspecific chronic duodenal inflammation and podoplanin positivity on immunohistochemistry. The patient was diagnosed with primary intestinal lymphangiectasia and nonspecific IBD. He was started on intravenous immunoglobulin replacement therapy (IgRT) and albumin infusions.

Supplementary Methods

Rare variant association data analysis in IBD cohorts

IBD diagnoses in the local registries were confirmed by gastrointestinal specialists according to the ECCO–ESGAR consensus guidelines[64], using a multimodal diagnostic approach integrating clinical evaluation, endoscopic findings, histopathology, laboratory results, and cross-sectional imaging. Only patients meeting these criteria were included. Non-IBD controls were individuals with available WES data and no clinical diagnosis of IBD. Individuals with known inborn errors of immunity (IEI) were excluded.

a) The family-based study population included Turkish and Qatari patients with IBD and non-IBD families with available whole-exome sequencing (WES) data. WES for both the Turkish and Qatari cohorts was generated using the same Illumina sequencing platform and processed through a unified bioinformatics pipeline. We analyzed 647 individuals (51.2% male) from 73 IBD families and 119 non-IBD families. The cohort included 74 IBD patients and 573 individuals without a clinical diagnosis of IBD, including individuals with unsolved genetic diseases and healthy relatives. Protein-altering and deleterious GPR15 variants with a minor allele frequency (MAF) < 0.1% were selected for analysis. In total, 11 rare variants met these criteria, including 1 stop-gain and 10 missense variants. Because all variants were monoallelic, carriers were recoded as *WT/M* for downstream analyses.

To account for non-independence among related individuals, we applied generalized linear mixed-effects models (GLMMs)[65] with a logit link (IBD vs non-IBD) using the lme4 package (v1.1-38) in R (v4.5.1) to test the association between GPR15 genotype and IBD status. Family was included as a random intercept to account for shared genetic and environmental structure, while sex, age, and ethnicity were included as fixed effects. Odds ratios and 95% confidence intervals (CIs) were obtained by exponentiating model coefficients.

b) In the population-based Iranian cohort[66], 1,692 individuals (59.7% male) with WES data were evaluated after excluding those with known IEI. Among these, 109 individuals had a confirmed diagnosis of IBD.

Principal component analysis (PCA) was performed to compare the genetic structure of IBD patients carrying GPR15 variants with other unsolved IEI patients within the same cohort. Variant call format (VCF) files were filtered to retain high-quality variants (Phred-scaled quality score ≥ 30 and depth ≥ 10). Single-nucleotide polymorphisms (SNPs) with a MAF < 0.05 were excluded to focus on common variants. Linkage disequilibrium (LD) pruning was applied using PLINK (v1.9) with the independent-pairwise algorithm to retain one representative SNP per LD block. The resulting genotype matrix was converted to tabular format, standardized, and used as input for PCA implemented in scikit-learn (v1.3.2). PCA was used to assess genetic diversity, ethnicity, clinical IEI diagnosis, and *GPR15* variant carriers. The analysis showed that IBD cases carrying heterozygous GPR15 variants belonged to different ethnic backgrounds and did not cluster separately, indicating no obvious population stratification or shared polymorphic genetic modifiers. Among all individuals analyzed, GPR15 variants with a MAF < 0.1% were retained for evaluation. A total of 9 rare variants met these criteria, including 1 stop-gain and 8 missense variants. All carriers were monoallelic. Multivariable logistic regression was used to calculate odds ratios for carriers of GPR15 variants, adjusting for age, sex, race, ethnicity, and the top 10 principal components of genetic ancestry.

Statistical significance was assessed using two-sided Wald tests in R (v4.5.1). Sex and ethnicity distributions between groups were compared using Fisher's exact test, whereas age distributions were

summarized as interquartile ranges (IQRs) and compared using the two-sided Mann–Whitney U test. Population characteristics, variant lists, and patient information are provided in the Source Data related to Extended Data Fig. 4a, b.

Machine learning analyses of *GPR15* variants in IBD within the All of Us population

A machine learning model was developed for IBD phenotype prediction using patient electronic health records (EHRs) from the All of Us genomic and EHR dataset[26] (n = 317,969). Several language model backbones were evaluated under the computational resource constraints of the All of Us environment. T5 achieved performance comparable to Qwen3-0.6B[67,68]. Given its efficiency, the T5-based classifier was used for the final analyses, consisting of a T5 encoder followed by two additional feedforward layers. For training hyperparameters, we used a batch size of 64, a learning rate of 5e-5, and trained for 3 epochs, saving the best checkpoint at the end of the training.

To avoid circularity between genotypes and phenotype definitions, IBD phenotypes were defined solely using diagnostic codes. In total, 6,116 confirmed individuals with IBD codes were identified based on ICD-9, ICD-10, and SNOMED codes mapped to phecodeX (version 1.2) for Crohn's disease (GI_522.11) and ulcerative colitis (GI_522.12)[69]. These cases were randomly matched with an equal number of individuals with no-IBD codes drawn from the remaining participants. Of the 12,232 participants selected, 1,000 individuals (500 with IBD codes and 500 with no-IBD codes) were set aside as an independent validation set, and the remaining individuals were used for training. For each patient, up to the 100 most recent lab measurements (e.g. body weight, serum albumin, platelet count) were collected, with each measurement type represented at most once per day and duplicate entries removed. Because patients had varying numbers of tests, a traditional feature-based classification approach would have introduced substantial missing values. Therefore, the problem was reframed as a text-classification task: each patient's measurements, along with dates and ranges, were concatenated into a single paragraph of text as model input, and the output label indicated EHR-defined IBD vs non-IBD phenotypes.

After training, the model was applied to all 316,973 participants with available PCA data to generate an IBD phenotype confidence score. Patients were classified as having IBD-related phenotypes at a threshold of > 0.5. This threshold was selected to increase case detection, and individuals newly classified as having IBD-related phenotypes typically exhibited clinically relevant features such as low body weight/BMI, hypoalbuminemia, abdominal pain, gastrointestinal hemorrhage, diarrhea, fatigue, elevated inflammatory markers (e.g., CRP, ESR), and abnormal colonoscopy findings[64]. Among the 6,116 confirmed individuals with IBD codes, 3,319 (54.3%) had a confidence score > 0.5. Among the 310,857 individuals with no-IBD codes, 65,728 (21.1%) were newly classified as having IBD-related phenotypes at the same threshold. The most frequent IBD-related features in these newly classified cases were ranked according to their frequency in the model input.

Among the 316,973 individuals analyzed, GPR15 variants were filtered and grouped as follows: deleterious variants identified in the four families (Y215X, Y132S, and D306N), 27 predicted loss-of-function (pLoF) variants (13 frameshift variants and 14 stop-gain mutations), 325 missense variants with a minor allele frequency (MAF) < 0.001, and 33 missense variants in the C-terminal region (G283-V358) of GPR15 with a CADD score > 20. Three synonymous variants in the GPR15 coding sequence were used as negative controls. Multivariable logistic regression was used to calculate odds ratios for these variant groups, adjusting for age, sex, race, ethnicity, and the top 10 principal components of genetic ancestry. Statistical

significance was assessed using two-sided Wald tests, and *P* values were adjusted for multiple comparisons using the Benjamini–Hochberg false discovery rate method implemented in statsmodels (v0.14.4) in Python (v3.10.16). The full list of variants and the top 15 IBD-related features are provided in the Source Data related to Extended Data Fig. 4f-h.

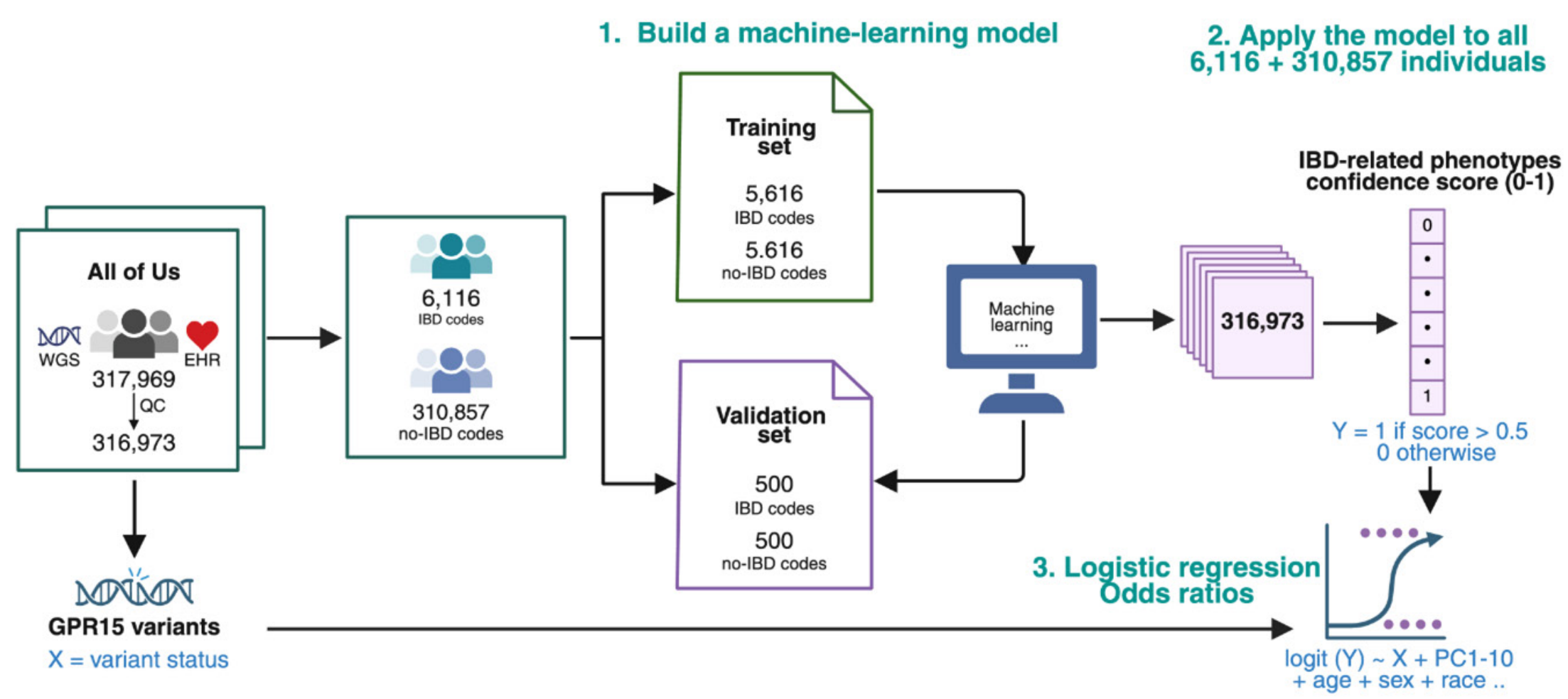

Schematic overview of machine learning-based phenotyping and GPR15 variant association analysis in the *All of Us* cohort. WGS, whole-genome sequencing; EHR, electronic health record. Odds ratios were calculated using a multivariable logistic regression model adjusted for age, sex, race, ethnicity, and the top 10 principal components (PC1-10) from genomic sequencing covariates for SNV analyses. Illustrations were created in BioRender. Cui, J. (2026) https://BioRender.com/2i0g4zm

Supplementary References

64 Sturm, A. *et al.* ECCO-ESGAR Guideline for Diagnostic Assessment in IBD Part 2: IBD scores and general principles and technical aspects. *J Crohns Colitis* **13**, 273-284 (2019).

65 Bolker, B. M. *et al.* Generalized linear mixed models: a practical guide for ecology and evolution. *Trends Ecol Evol* **24**, 127-135 (2009).

66 Aghamohammadi, A. *et al.* Consensus Middle East and North Africa Registry on Inborn Errors of Immunity. *J Clin Immunol* **41**, 1339-1351 (2021).

67 Raffel, C. *et al.* Exploring the Limits of Transfer Learning with a Unified Text-to-Text Transformer. *J Mach Learn Res* **21** (2020).

68 Yang, A. *et al.* Qwen3 technical report. *arXiv preprint arXiv:2505.09388* (2025).

69 Shuey, M. M. *et al.* Next-generation phenotyping: introducing phecodeX for enhanced discovery research in medical phenomics. *Bioinformatics* **39** (2023).
