# Supplementary material for: GPR15-guided CD8+ T regulatory cells control intestinal inflammation
Source: Nature. 2026 Jun 8;656(8129):1003–12. doi: 10.1038/s41586-026-10749-4 (PMC13518229; doi:10.1038/s41586-026-10749-4)
Supplement: Supplementary file 2 [file 41586_2026_10749_MOESM2_ESM.pdf]

nature portfolio

Corresponding author(s): Michael Lenardo

Last updated by author(s): May 6, 2026

Reporting Summary

Nature Portfolio wishes to improve the reproducibility of the work that we publish. This form provides structure for consistency and transparency in reporting. For further information on Nature Portfolio policies, see our Editorial Policies and the Editorial Policy Checklist.

Statistics

For all statistical analyses, confirm that the following items are present in the figure legend, table legend, main text, or Methods section.

n/a	Confirmed	
☐	☒	The exact sample size (*n*) for each experimental group/condition, given as a discrete number and unit of measurement
☐	☒	A statement on whether measurements were taken from distinct samples or whether the same sample was measured repeatedly
☐	☒	The statistical test(s) used AND whether they are one- or two-sided<br>*Only common tests should be described solely by name; describe more complex techniques in the Methods section.*
☐	☒	A description of all covariates tested
☐	☒	A description of any assumptions or corrections, such as tests of normality and adjustment for multiple comparisons
☐	☒	A full description of the statistical parameters including central tendency (e.g. means) or other basic estimates (e.g. regression coefficient) AND variation (e.g. standard deviation) or associated estimates of uncertainty (e.g. confidence intervals)
☐	☒	For null hypothesis testing, the test statistic (e.g. *F*, *t*, *r*) with confidence intervals, effect sizes, degrees of freedom and *P* value noted<br>*Give P values as exact values whenever suitable.*
☒	☐	For Bayesian analysis, information on the choice of priors and Markov chain Monte Carlo settings
☒	☐	For hierarchical and complex designs, identification of the appropriate level for tests and full reporting of outcomes
☒	☐	Estimates of effect sizes (e.g. Cohen's *d*, Pearson's *r*), indicating how they were calculated

Our web collection on statistics for biologists contains articles on many of the points above.

Software and code

Policy information about availability of computer code

Data collection	Flow cytometry data were acquired using FACSDiva (v9.1, v9.0, BD Biosciences) on FACSymphony A3 (BD Biosciences) and LSRFortessa (BD Biosciences), and cell sorting was performed on FACSAria and FACS Fusion (BD Biosciences). Chemiluminescence data were collected using Azure C500 imaging system (v1.7.6.1202). Immunofluorescence images were captured on ZEISS Axio Vert.A1 TL/FL-LED using ZEN (v3.10, ZEISS), Leica SP8 WLL FLIM, or PhenoImager (Akoya Biosciences). Whole-exome sequencing was performed on Illumina HiSeq 2500, and single-cell CITE-seq and TCR V(D)J libraries were generated using 10x Genomics Chromium platform for single-cell capture and cDNA preparation and Illumina NovaSeq Xplus 10B or DNBSEQ-T7 PE100 (BGI) for sequencing.
Data analysis	Flow cytometry data were analyzed using FlowJo (Tree Star, v10.9.0). Images were processed with ImageJ (v2.14.0), Imaris (v10.2.0), QuPath (v0.5.1), LAS X(Leica, v3.5.7), and SimpleITK (PMID: 29181613).<br>Single-cell RNA-seq, CITE-seq, and TCR V(D)J analyses were processed with 10x Genomics CellRanger multi (v9.0) and CellRanger count (v6.1.2), and analyzed in R (v4.4.0) using Seurat (v5.0.3), SingleR (v2.6.0), and scRepertoire (v2.4.0). Functional enrichment analyses were performed with SIGNAL (PMID: 33894945) and DAVID (PMID: 19131956). Heatmaps were generated using the pheatmap package (v1.0.13). Gene set enrichment analysis (GSEA) was performed using the fgsea package (v1.36.2). Volcano plots were generated using the ggplot2 package (v4.0.2). Additional analyses were conducted in Python using Scanpy (v1.9.1).<br>Bulk exome and genome sequencing reads were aligned using the Burrows–Wheeler Aligner (BWA, v0.7.17), and variants were identified using the Genome Analysis Toolkit (GATK, v3.5). Genetic association and population structure analyses were performed using PLINK (v1.9), scikit-learn (v1.3.2), generalized linear mixed-effects models implemented with lme4 (v1.1-38) in R(v4.5.1), and multivariable logistic regression in R (v4.5.1) or with statsmodels (v0.14.4) in Python (v3.10.16).<br>General statistical analyses and data visualization were conducted using GraphPad Prism (v10.2.0).

For manuscripts utilizing custom algorithms or software that are central to the research but not yet described in published literature, software must be made available to editors and reviewers. We strongly encourage code deposition in a community repository (e.g. GitHub). See the Nature Portfolio guidelines for submitting code & software for further information.

Data

Policy information about availability of data

All manuscripts must include a data availability statement. This statement should provide the following information, where applicable:

- Accession codes, unique identifiers, or web links for publicly available datasets
- A description of any restrictions on data availability
- For clinical datasets or third party data, please ensure that the statement adheres to our policy

Human whole exome and genome sequencing data from the four families evaluated at NIH have been deposited in dbGaP (phs003950.v1.p1).
Raw WES data for the Turkish/Qatari and Iranian cohorts are available under controlled access due to ethical and legal restrictions related to patient privacy and the terms of informed consent. Requests for access should be directed to Bernice Lo (blo@sidra.org) for the Turkish and Qatari cohorts and Hassan Abolhassani (Seyed.Abolhassani@ukbonn.de) for the Iranian cohort and are subject to institutional ethics approval and data use agreements.
Human and mouse single-cell RNA-Seq datasets generated in this study have been deposited in Gene Expression Omnibus (GEO) (GSE314446). Raw human scRNA-seq reads are available through dbGaP (phs003950.v1.p1).
Single-cell RNA-seq data from healthy adult and pediatric guts were obtained from the Gut Cell Atlas (https://www.gutcellatlas.org/), with raw data available at ArrayExpress (E-MTAB-9543, E-MTAB-9536, E-MTAB-9532, E-MTAB-9533 and E-MTAB-10386) (PMID: 34497389).
Single-cell RNA-seq data from the UC patients were obtained from GEO (Accession GSE125527) (PMID: 32826341).
Single-cell RNA-seq data from the CD patients were obtained from the Broad Institute Single Cell Portal (project SCP1884; https://singlecell.broadinstitute.org/single_cell/), with raw data available at Broad DUOS (Accession DUOS-000146 CD_Atlas_2021_GIDER; DUOS-000145 CD_Atlas_2021_PRISM) (PMID: 38091954).
Single-cell transcriptomic data of blood KIR+CD8+ T cells was obtained from GEO (Accession GSE193770) (PMID: 35258337).
AoU dataset were obtained from the All of Us Research Program (Controlled Tier Dataset v8, https://www.researchallofus.org/) (PMID: 31412182).
UK Biobank analysis were assessed through GeneBass (https://app.genebass.org/) (PMID: 36778668).
Population allele frequencies were obtained from gnomAD (https://gnomad.broadinstitute.org/) (PMID: 32461654).
Variant deleteriousness scores were derived from CADD (https://cadd.gs.washington.edu/) (PMID: 24487276).
Protein structural predictions were obtained from AlphaFold (https://alphafold.ebi.ac.uk/) (PMID: 34265844).

Research involving human participants, their data, or biological material

Policy information about studies with human participants or human data. See also policy information about sex, gender (identity/presentation), and sexual orientation and race, ethnicity and racism.

Reporting on sex and gender	Patients and relatives were enrolled for evaluation without regard to sex or gender.
Reporting on race, ethnicity, or other socially relevant groupings	Patients and relatives were enrolled for evaluation without regard to race, ethnicity, or other socially relevant groupings.
Population characteristics	Participants included individuals with and without GPR15 mutations, healthy relatives, individuals with unsolved genetic diseases, and patients with IBD. Covariate-relevant characteristics, including age, sex, race, or ethnicity, disease status, and GPR15 genotype, were recorded and considered in analyses.
Recruitment	Participants were recruited during clinical care or derived from public datasets, including the All of Us Research Program. Clinical recruitment may introduce selection bias toward affected individuals, while population datasets may reflect self-selection bias; however, genotype- and disease-based grouping is unlikely to materially affect the primary conclusions.
Ethics oversight	Study subjects, including patients, relatives, and healthy controls, provided written informed consent under National Institutes of Health (NIH) protocol 06-I-0015 (ClinicalTrials.gov NCT00246857), protocol 1500768 approved by the Sidra Medicine and Marmara Medical Center Institutional Review Board (IRBs), and protocol IR.TUMS.CHMC.REC.1398.030 approved by the Tehran University of Medical Sciences Ethics Committee, in accordance with local ethics and IRB regulations. Collection of blood and leftover biopsy samples (collected when medically indicated) are covered by these protocols. Blood samples from anonymized healthy donors were obtained at the NIH Clinical Center under approved protocols.

Note that full information on the approval of the study protocol must also be provided in the manuscript.

Field-specific reporting

Please select the one below that is the best fit for your research. If you are not sure, read the appropriate sections before making your selection.

☒ Life sciences ☐ Behavioural & social sciences ☐ Ecological, evolutionary & environmental sciences

For a reference copy of the document with all sections, see nature.com/documents/nr-reporting-summary-flat.pdf

Life sciences study design

All studies must disclose on these points even when the disclosure is negative.

Sample size	Sample size was not pre-determined by statistical calculation. For in vitro and in vivo experiments, at least n = 3 biological replicates per group were used. For single-cell RNA-seq analyses, some groups included data from two individuals, with large cell numbers supporting robust

analysis. Sample sizes were determined based on similar previously published studies (e.g., PMID: 32647003, PMID: 24165795, PMID: 26206937, PMID: 38172257, PMID: 35303419, PMID: 33771991).

Data exclusions	No data were excluded.
Replication	All experiments were reproducible and often performed independently by different members of the research team. Where applicable, independent experiments were repeated 3–5 times. Exceptions include single-cell RNA-seq analyses and histological/endoscopic assessments in IBD patients, in which two independent endoscopic examination results during active IBD were collected per patient. Reproducibility of histological findings was confirmed by adjacent-section staining.
Randomization	For animal experiments, WT and mutant mice were randomly allocated to untreated or treated conditions. For human samples, randomization was not performed, as comparisons were based on biological groupings, including GPR15 mutation status and disease status. For population-based analyses, samples were similarly grouped by clinical diagnosis or GPR15 genotype rather than random assignment. Potential covariates, including age, sex, and race or ethnicity, were accounted for in the analyses.
Blinding	Blinding was not applied during group allocation. In both animal and human studies, investigators were not blinded to genotype, as group assignment was based on GPR15 genotype and required accurate sample identification. For histological image analysis, genotypes were blinded to ensure unbiased quantification.

Reporting for specific materials, systems and methods

We require information from authors about some types of materials, experimental systems and methods used in many studies. Here, indicate whether each material, system or method listed is relevant to your study. If you are not sure if a list item applies to your research, read the appropriate section before selecting a response.

Materials & experimental systems

n/a	Involved in the study	
☐	☒	Antibodies
☐	☒	Eukaryotic cell lines
☒	☐	Palaeontology and archaeology
☐	☒	Animals and other organisms
☒	☐	Clinical data
☒	☐	Dual use research of concern
☒	☐	Plants

Methods

n/a	Involved in the study	
☒	☐	ChIP-seq
☐	☒	Flow cytometry
☒	☐	MRI-based neuroimaging

Antibodies

Antibodies used

Immunoblotting:
mouse anti-human GPR15 367902 R&D Systems MAB3654 1:1000
mouse anti-HSP90 K3720A BioLegend 661802 1:5000
rabbit anti-β-Arrestin 1/2 D24H9 Cell Signaling 4674 1:1000
rabbit anti-14-3-3 (pan) E9S9M Cell Signaling 95422 1:1000
rabbit anti-Flag SIG1-25 Sigma F2555 1:1000
goat anti-mouse IgG HRP SouthernBiotech 1030-05 1:2000
goat anti-rabbit IgG HRP SouthernBiotech 4050-05 1:2000
mouse anti-rabbit IgG HRP (TrueBlot) eB182 Rockland 18-8816-31 1:1000

Co-immunoprecipitation:
Rat anti-Flag L5 BioLegend 637303 Co-IP: 5 μg antibody/1.5 mg Dynabeads Protein G per sample

Cell immunofluorescence staining and confocal microscopy:
rabbit anti-calnexin C5C9 Cell Signaling 2679 1:200
rabbit anti-giantin Poly23A01 BioLegend 621352 1:1000
Alexa Fluor Plus 594 Goat anti-Rabbit IgG (H+L) Invitrogen A32740 1:1000
Alexa Fluor 488 Donkey anti-Mouse IgG (H+L) Invitrogen A21202 1:1000

Immunohistochemistry (IHC) and multiplex immunofluorescence staining:
rabbit anti-CD11c AF647 EP1347Y Abcam ab275338 1:50
mouse anti-CD68 iFluor 594 KP-1 Caprico Biotechnologies 164136 1:50
mouse anti-HLA-DR AF532 TAL1B5 Novus Biologicals NB600-989AF532 1:500
rabbit anti-CD3 SP7 Abcam ab16669 1:50
Goat anti-Rabbit IgG (H+L) AF532 ThermoFisher A11009 1:250
rabbit anti-CD4 EPR6855 Abcam ab133616 1:1000
rabbit anti-FOXP3 SP97 ThermoFisher MA5-16365 1:200
rabbit anti-CD8 EPR10640(2) Abcam ab189926 1:1000
mouse anti-CD3 LN10 Leica PA0553 Ready-to-use
rabbit anti-human GPR15 Sigma HPA013775 1:50
rat anti-F4/80 BM8 ThermoFisher 14-4801-82 1:150
rabbit anti-CD206 Abcam ab64693 1:1000

rabbit anti-NOS2 Abcam ab15323 1:100

Macrophage killing blocking antibodies:
anti-mouse TNFα QA19A26 BioLegend 935903 10 μg/mL
anti-mouse IFNγ R4-6A2 BioLegend 505708 10 μg/mL
anti-mouse FasL MFL3 BioLegend 106611 10 μg/mL
anti-mouse TWEAK MTW-1 BioLegend 120009 10 μg/mL
anti-mouse TRAIL N2B2 BioLegend 109315 10 μg/mL
anti-mouse H-2 M1/42 BioLegend 125502 10 μg/mL

Human flow antibodies:
APC/Cyanine7 anti-human CD3 344818 BioLegend SK7 1:100
APC anti-human GPR15 373006 BioLegend SA302A10 1:100
PE anti-human CD197 (CCR7) 353204 BioLegend G043H7 1:100
PerCP/Cyanine5.5 anti-human CD4 300530 BioLegend RPA-T4 1:100
PE/Cyanine7 anti-human CD45RA 983006 BioLegend HI100 1:100
Alexa Fluor 700 anti-human CD8 344724 BioLegend SK1 1:100
PE anti-human CD183 (CXCR3) 989502 BioLegend G025H7 1:100
Alexa Fluor 488 anti-human CD38 303512 BioLegend HIT2 1:100
Brilliant Violet 421 anti-human HLA-DR 307636 BioLegend L243 1:100
Alexa Fluor 488 anti-human CD196 (CCR6) 353414 BioLegend G034E3 1:100
PE/Cyanine7 anti-human CD27 302838 BioLegend O323 1:100
APC/Cyanine7 anti-human CD19 363009 BioLegend SJ25C1 1:100
Brilliant Violet 510 anti-human IgD 348220 BioLegend IA6-2 1:100
Alexa Fluor 700 anti-human CD20 302322 BioLegend 2H7 1:100
FITC anti-human CD14 325604 BioLegend HCD14 1:100
PE anti-human CD56 362508 BioLegend 5.1H11 1:100
PerCP/Cyanine5.5 anti-human CD123 306016 BioLegend H6 1:100
PE/Cyanine7 anti-human CD11c 371504 BioLegend S-HCL-3 1:100
Brilliant Violet 510 anti-human CD16 302048 BioLegend 3G8 1:100
Alexa Fluor 488 anti-human CD127 351314 BioLegend A019D5 1:100
PE anti-human FOXP3 320008 BioLegend 150D 1:100
Brilliant Violet 605 anti-human CD25 302631 BioLegend M-A251 1:100
APC/Cyanine7 anti-human CD45RO 304227 BioLegend UCHL1 1:100
Brilliant Violet 711 anti-human CD3 344838 BioLegend SK7 1:100
Alexa Fluor 488 anti-human CD56 (NCAM) 362518 BioLegend 5.1H11 1:100
FITC anti-human CD69 310904 BioLegend FN50 1:100
PE anti-human CD25 985802 BioLegend M-A251 1:400

Mouse flow antibodies:
PE anti-mouse GPR15 154604 BioLegend S15042I 1:150
PerCP/Cyanine5.5 anti-mouse TCR γ/δ 118118 BioLegend GL3 1:200
Brilliant Violet 650 anti-mouse CD8a 100742 BioLegend 53-6.7 1:200
PE/Cyanine7 anti-mouse TCR β chain 109222 BioLegend H57-597 1:150
APC/Cyanine7 anti-mouse CD3 100222 BioLegend 17A2 1:150
Brilliant Violet 421 anti-mouse CD8β 126629 BioLegend Ly-3 1:200
Brilliant Violet 711 anti-mouse CD4 100557 BioLegend RM4-5 1:200
Alexa Fluor 700 anti-mouse CD45 103128 BioLegend 30-F11 1:200
Brilliant Violet 605 anti-mouse CD19 115540 BioLegend 6D5 1:100
Alexa Fluor 647 anti-mouse CD45.1 110720 BioLegend A20 1:200
Alexa Fluor 700 anti-mouse CD45.2 109822 BioLegend 104 1:200
PE anti-mouse F4/80 157303 BioLegend QA17A29 1:100
PE-Cy7 Hamster Anti-Mouse CD11c 558079 BD Biosciences HL3 1:100
BUV737 Rat Anti-CD11b 612800 BD Biosciences M1/70 1:100
BUV395 Rat Anti-Mouse I-A, I-E (MHC-II) 569244 BD Biosciences 2G9 1:400
APC anti-mouse CD103 156914 BioLegend QA17A24 1:150
BUV737 anti-mouse CD4 612761 BD Biosciences GK1.5 1:200
APC-eFluor 780 anti-mouse CD8a 47-0081-82 eBioscience 53-6.7 1:200
PerCP/Cyanine5.5 anti-mouse CD5 100624 BioLegend 53-7.3 1:200
BV711 anti-mouse TCR β chain 563135 BD Biosciences H57-597 1:200
APC anti-mouse CD122 (IL-2Rβ) 123213 BioLegend TM-β1 1:100
PE-Cy7 anti-mouse MHC Class I (H-2Kb) 25-5958-82 eBioscience AF6-88.5.5.3 1:200
Alexa Fluor 700 anti-mouse CD44 56-0441-82 eBioscience IM7 1:200
eFluor 450 anti-mouse CD279 (PD-1) 48-9985-82 eBioscience J43 1:200

Flow blocking:
Human TruStain FcX 422302 BioLegend RUO 1:100
TruStain FcX PLUS (anti-mouse CD16/32) Antibody 156604 BioLegend S17011E 1:100

Mouse Total SeqA antibodies:
CD4 RM4-5 AACAAGACCCTTGAG BioLegend CD4 1:200
CD8a 53-6.7 TACCCGTAATAGCGT BioLegend CD8a 1:200
TCRb H57-597 TCCTATGGGACTCAG BioLegend TCR β chain 1:200
CD44 IM7 TGGCTTCAGGTCCTA BioLegend CD44 1:100
CD62L MEL-14 TGGGCCTAAGTCATC BioLegend CD62L 1:100
TCRgd GL3 AACCCAAATAGCTGA BioLegend TCRγ/δ 1:100

CD8b YTS156.7.7 TTCCCTCTATGGAGC BioLegend CD8b 1:100
GPR56 CG4 GCCTAGTTTCCGTTT BioLegend GPR56 1:100
CD94 18d3 CACAGTTGTCCGTGT BioLegend CD94 1:100
Ly49D 4E5 TATATCCCTCAACGC BioLegend Ly49D 1:100
Ly49G AT8 CGTATCTGTCATTAG BioLegend Ly49G 1:100
Ly49H 3D10 CCAGTAGGCTTATTA BioLegend Ly49H 1:100
CD103 2E7 TTCATTAGCCCGCTG BioLegend CD103 1:50
CCR7 4B12 TTATTAACAGCCCAC BioLegend CCR7 1:50
PD1 RMP1-30 GAAAGTCAAAGCACT BioLegend PD1 1:50
CD69 H1.2F3 TTGTATTCCGCCATT BioLegend CD69 1:50
CXCR4 L276F12 GTCGTGGTGTTGTTC BioLegend CXCR4 1:50
Mouse IgG1, κ isotype Ctrl Antibody MOPC-21 GCCGGACGACATTAA BioLegend Mouse IgG1, κ isotype Ctrl 1:200
Rat IgG2a, κ Isotype Ctrl Antibody RTK2758 AAGTCAGGTTCGTTT BioLegend Rat IgG2a, κ Isotype Ctrl 1:200
Rat IgG2b, κ Isotype Ctrl Antibody RTK4530 GATTCTTGACGACCT BioLegend Rat IgG2b, κ isotype Ctrl 1:200
Armenian Hamster IgG Isotype Ctrl Antibody HTK888 CCTGTCATTAAGACT BioLegend Armenian Hamster IgG Isotype Ctrl 1:200

Mouse Hashtag antibodies:
TotalSeq™-A0301 anti-mouse Hashtag 1 Antibody M1/42 ACCCACCAGTAAGAC BioLegend 155801 1:50
TotalSeq™-A0302 anti-mouse Hashtag 2 Antibody M1/42 GGTCGAGAGCATTCA BioLegend 155803 1:50
TotalSeq™-A0303 anti-mouse Hashtag 3 Antibody M1/42 CTTGCCGCATGTCAT BioLegend 155805 1:50
TotalSeq™-A0304 anti-mouse Hashtag 4 Antibody M1/42 AAAGCATTCTTCACG BioLegend 155807 1:50

Human Total SeqC antibodies:
Hu.GPR15 SA302A10 ATGATCTGTGCCGGA BioLegend 373021 1:50
TotalSeq™-C Human Universal Cocktail V1.0 BioLegend 399905 Read-to-use

Human Hashtag antibodies:
HTO-1 LNH-94; 2M2 GTCAACTCTTTAGCG BioLegend 394661 1:50
HTO-2 LNH-94; 2M2 TGATGGCCTATTGGG BioLegend 394663 1:50
HTO-3 LNH-94; 2M2 TTCCGCCTCTCTTTG BioLegend 394665 1:50
HTO-4 LNH-94; 2M2 AGTAAGTTCAGCGTA BioLegend 394667 1:50
HTO-5 LNH-94; 2M2 AAGTATCGTTTCGCA BioLegend 394669 1:50
HTO-6 LNH-94; 2M2 GGTTGCCAGATGTCA BioLegend 394671 1:50
HTO-7 LNH-94; 2M2 TGTCTTTCCTGCCAG BioLegend 394673 1:50
HTO-8 LNH-94; 2M2 CTCCTCTGCAATTAC BioLegend 394675 1:50
HTO-9 LNH-94; 2M2 CAGTAGTCACGGTCA BioLegend 394677 1:50

Validation

All antibodies used came from commercial vendors, and we based specificity on their provided description and data sheets.

Immunoblotting:
mouse anti-human GPR15 https://www.rndsystems.com/products/human-gpr15-antibody-367902_mab3654
mouse anti-HSP90 https://www.biolegend.com/en-us/products/purified-anti-hsp90alpha-beta-antibody-10275
rabbit anti-β-Arrestin 1/2 https://www.cellsignal.com/products/primary-antibodies/beta-arrestin-1-2-d24h9-rabbit-monoclonal-antibody/4674
rabbit anti-14-3-3 (pan) https://www.cellsignal.com/products/primary-antibodies/14-3-3-pan-e9s9m-rabbit-monoclonal-antibody/95422
rabbit anti-Flag https://www.sigmaaldrich.com/US/en/product/sigma/f2555
goat anti-mouse IgG HRP https://www.southernbiotech.com/goat-anti-mouse-igg-human-ads-hrp-1030-05
goat anti-rabbit IgG HRP https://www.southernbiotech.com/goat-anti-rabbit-igg-h-l-mouse-human-ads-hrp-4050-05
mouse anti-rabbit IgG HRP (TrueBlot) https://www.rockland.com/categories/trueblot/rabbit-trueblot-ultra-anti-rabbit-igg-hrp-18-8816-31/

Co-immunoprecipitation:
Rat anti-Flag https://www.biolegend.com/en-us/products/purified-anti-dykddddk-tag-antibody-4905

Cell immunofluorescence staining and confocal microscopy:
rabbit anti-calnexin https://www.cellsignal.com/products/primary-antibodies/calnexin-c5c9-rabbit-monoclonal-antibody/2679
rabbit anti-giantin https://www.biolegend.com/en-us/products/purified-anti-giantin-antibody-23907
Alexa Fluor Plus 594 Goat anti-Rabbit IgG (H+L) https://www.fishersci.com/shop/products/goat-anti-rabbit-igg-h-l-highly-cross-adsorbed-secondary-antibody-alexa-fluor-plus-594/PIA32740#?keyword=A32740
Alexa Fluor 488 Donkey anti-Mouse IgG (H+L) https://www.fishersci.com/shop/products/donkey-anti-mouse-igg-h-l-highly-cross-adsorbed-secondary-antibody-alexa-fluor-488/A21202#?keyword=A21202

Immunohistochemistry (IHC) and multiplex immunofluorescence staining:
rabbit anti-CD11c AF647 https://www.abcam.com/en-us/products/primary-antibodies/alexa-fluor-647-cd11c-antibody-ep1347y-ab275338
mouse anti-CD68 iFluor 594 https://www.capricobio.com/product/cd68-kp1-ifluor-594/
mouse anti-HLA-DR AF532 https://www.rndsystems.com/products/hla-dr-antibody-tal-1b5_nb600-989af532
rabbit anti-CD3 https://www.abcam.com/en-us/products/primary-antibodies/cd3-epsilon-antibody-sp7-ab16669
Goat anti-Rabbit IgG (H+L) AF532 https://www.fishersci.com/shop/products/goat-anti-rabbit-igg-h-l-cross-adsorbed-secondary-antibody-alexa-fluor-532/A11009#?keyword=A11009
rabbit anti-CD4 https://www.abcam.com/en-us/products/primary-antibodies/cd4-antibody-epr6855-ab133616
rabbit anti-FOXP3 https://www.fishersci.com/shop/products/foxp3-monoclonal-antibody-sp97/PIMA516365?searchHijack=true&searchTerm=MA5-16365&searchType=RAPID&matchedCatNo=MA5-16365
rabbit anti-CD8 https://www.abcam.com/en-us/products/primary-antibodies/cd8-alpha-antibody-epr106402-n-terminal-ab189926
mouse anti-CD3 https://shop.leicabiosystems.com/us/ihc-ish/ihc-primary-antibodies/pid-cd3?

srsltid=AfmBOorPCL3JXJeEQaIHyk02vyq2b33zqpSeUFXDr8IJZM2eWoDRsAdX
rabbit anti-human GPR15 https://www.sigmaaldrich.com/US/en/product/sigma/hpa013775
rat anti-F4/80 https://www.fishersci.com/shop/products/f4-80-monoclonal-antibody-bm8-ebioscience-invitrogen/501129661?searchHijack=true&searchTerm=14-4801-82&searchType=RAPID&matchedCatNo=14-4801-82
rabbit anti-CD206 https://www.abcam.com/en-us/products/primary-antibodies/mannose-receptor-antibody-ab64693
rabbit anti-NOS2 https://www.abcam.com/en-us/products/primary-antibodies/inos-antibody-ab15323

Macrophage killing blocking antibodies:
anti-mouse TNFα https://www.biolegend.com/en-us/products/ultra-leaf-purified-anti-mouse-tnf-alpha-recombinant-antibody-20426
anti-mouse IFNγ https://www.biolegend.com/en-us/products/ultra-leaf-purified-anti-mouse-ifn-gamma-antibody-18657
anti-mouse FasL https://www.biolegend.com/en-us/products/ultra-leaf-purified-anti-mouse-cd178-fasl-antibody-18343
anti-mouse TWEAK https://www.biolegend.com/en-us/products/ultra-leaf-purified-anti-mouse-cd255-tweak-antibody-19384
anti-mouse TRAIL https://www.biolegend.com/en-us/products/ultra-leaf-purified-anti-mouse-cd253-trail-antibody-18254
anti-mouse H-2 https://www.biolegend.com/en-us/products/purified-anti-mouse-h-2-antibody-5344

Human flow antibodies
APC/Cyanine7 anti-human CD3 https://www.biolegend.com/en-us/products/apc-cyanine7-anti-human-cd3-antibody-6940
APC anti-human GPR15 https://www.biolegend.com/en-us/products/apc-anti-human-gpr15-antibody-13952
PE anti-human CD197 (CCR7) https://www.biolegend.com/en-us/products/pe-anti-human-cd197-ccr7-antibody-7498
PerCP/Cyanine5.5 anti-human CD4 https://www.biolegend.com/en-us/products/percp-cyanine5-5-anti-human-cd4-antibody-4216
PE/Cyanine7 anti-human CD45RA https://www.biolegend.com/en-us/products/pe-cyannine7-anti-human-cd45ra-21929
Alexa Fluor 700 anti-human CD8 https://www.biolegend.com/en-us/products/alexa-fluor-700-anti-human-cd8-antibody-9062
PE anti-human CD183 (CXCR3) https://www.biolegend.com/en-us/products/pe-anti-human-cd183-25983
Alexa Fluor 488 anti-human CD38 https://www.biolegend.com/en-us/products/alexa-fluor-488-anti-human-cd38-antibody-3263
Brilliant Violet 421 anti-human HLA-DR https://www.biolegend.com/en-us/products/brilliant-violet-421-anti-human-hla-dr-antibody-7226
Alexa Fluor 488 anti-human CD196 (CCR6) https://www.biolegend.com/en-us/products/alexa-fluor-488-anti-human-cd196-ccr6-antibody-7562
PE/Cyanine7 anti-human CD27 https://www.biolegend.com/en-us/products/pe-cyanine7-anti-human-cd27-antibody-8434
APC/Cyanine7 anti-human CD19 https://www.biolegend.com/en-us/products/apc-cyanine7-anti-human-cd19-antibody-10302
Brilliant Violet 510 anti-human IgD https://www.biolegend.com/en-us/products/brilliant-violet-510-anti-human-igd-antibody-8012
Alexa Fluor 700 anti-human CD20 https://www.biolegend.com/en-us/products/alexa-fluor-700-anti-human-cd20-antibody-3400
FITC anti-human CD14 https://www.biolegend.com/en-us/products/fitc-anti-human-cd14-antibody-3951
PE anti-human CD56 https://www.biolegend.com/en-us/products/pe-anti-human-cd56-ncam-antibody-9958
PerCP/Cyanine5.5 anti-human CD123 https://www.biolegend.com/en-us/products/percp-cyanine5-5-anti-human-cd123-antibody-4244
PE/Cyanine7 anti-human CD11c https://www.biolegend.com/en-us/products/pe-anti-human-cd11c-antibody-13285
Brilliant Violet 510 anti-human CD16 https://www.biolegend.com/en-us/products/brilliant-violet-510-anti-human-cd16-antibody-8003
Alexa Fluor 488 anti-human CD127 https://www.biolegend.com/en-us/products/alexa-fluor-488-anti-human-cd127-il-7ralpha-antibody-7171
PE anti-human FOXP3 https://www.biolegend.com/en-us/products/pe-anti-mouse-rat-human-foxp3-antibody-2889
Brilliant Violet 605 anti-human CD25 https://www.biolegend.com/en-us/products/brilliant-violet-605-anti-human-cd25-antibody-7658
APC/Cyanine7 anti-human CD45RO https://www.biolegend.com/en-us/products/apc-cyanine7-anti-human-cd45ro-antibody-7372
Brilliant Violet 711 anti-human CD3 https://www.biolegend.com/en-us/products/brilliant-violet-711-anti-human-cd3-antibody-12007
Alexa Fluor 488 anti-human CD56 (NCAM) https://www.biolegend.com/en-us/products/alexa-fluor-488-anti-human-cd56-ncam-antibody-10245
FITC anti-human CD69 https://www.biolegend.com/en-us/products/fitc-anti-human-cd69-antibody-1671
PE anti-human CD25 https://www.biolegend.com/en-us/products/pe-anti-human-cd25-antibody-20955

Mouse flow antibodies:
PE anti-mouse GPR15 https://www.biolegend.com/en-us/products/pe-anti-mouse-gpr15-bob-antibody-15609
PerCP/Cyanine5.5 anti-mouse TCR γ/δ https://www.biolegend.com/en-us/products/percp-cyanine5-5-anti-mouse-tcr-gamma-delta-antibody-6702
Brilliant Violet 650 anti-mouse CD8a https://www.biolegend.com/en-us/products/brilliant-violet-650-anti-mouse-cd8a-antibody-7635
PE/Cyanine7 anti-mouse TCR β chain https://www.biolegend.com/en-us/products/pe-cyanine7-anti-mouse-tcr-beta-chain-antibody-4144
APC/Cyanine7 anti-mouse CD3 https://www.biolegend.com/en-us/products/apc-cyanine7-anti-mouse-cd3-antibody-6068
Brilliant Violet 421 anti-mouse CD8β https://www.biolegend.com/en-us/products/brilliant-violet-421-anti-mouse-cd8b-ly-3-antibody-17374
Brilliant Violet 711 anti-mouse CD4 https://www.biolegend.com/en-us/products/brilliant-violet-711-anti-mouse-cd4-antibody-7925
Alexa Fluor 700 anti-mouse CD45 https://www.biolegend.com/en-us/products/alexa-fluor-700-anti-mouse-cd45-antibody-3407
Brilliant Violet 605 anti-mouse CD19 https://www.biolegend.com/en-us/products/brilliant-violet-605-anti-mouse-cd19-antibody-7645
Alexa Fluor 647 anti-mouse CD45.1 https://www.biolegend.com/en-us/products/alexa-fluor-647-anti-mouse-cd45-1-antibody-3104
Alexa Fluor 700 anti-mouse CD45.2 https://www.biolegend.com/en-us/products/alexa-fluor-700-anti-mouse-cd45-2-antibody-3393
PE anti-mouse F4/80 https://www.biolegend.com/en-us/products/pe-anti-mouse-f4-80-recombinant-antibody-18755
PE-Cy7 Hamster Anti-Mouse CD11c https://www.bdbiosciences.com/en-us/products/reagents/flow-cytometry-reagents/research-reagents/single-color-antibodies-ruo/pe-cy-7-hamster-anti-mouse-cd11c.558079?tab=product_details
BUV737 Rat Anti-CD11b https://www.bdbiosciences.com/en-us/products/reagents/flow-cytometry-reagents/research-reagents/single-color-antibodies-ruo/buv737-rat-anti-cd11b.612800?tab=product_details
BUV395 Rat Anti-Mouse I-A, I-E (MHC-II) https://www.bdbiosciences.com/en-us/products/reagents/flow-cytometry-reagents/research-reagents/single-color-antibodies-ruo/buv395-rat-anti-mouse-i-a-i-e.569244?tab=product_details
APC anti-mouse CD103 https://www.biolegend.com/en-us/products/apc-anti-mouse-cd103-recombinant-antibody-21155

BUV737 anti-mouse CD4 https://www.bdbiosciences.com/en-pt/products/reagents/flow-cytometry-reagents/research-reagents/single-color-antibodies-ruo/buv737-rat-anti-mouse-cd4.612761?utm_source=chatgpt.com&tab=product_details
APC-eFluor 780 anti-mouse CD8a https://www.thermofisher.com/antibody/product/CD8a-Antibody-clone-53-6-7-Monoclonal/47-0081-82?utm_source=chatgpt.com
PerCP/Cyanine5.5 anti-mouse CD5 https://www.biolegend.com/en-us/products/percp-cyanine5-5-anti-mouse-cd5-antibody-9760
BV711 anti-mouse TCR β chain https://www.bdbiosciences.com/en-us/products/reagents/flow-cytometry-reagents/research-reagents/single-color-antibodies-ruo/bv711-hamster-anti-mouse-tcr-chain.563135?tab=product_details
APC anti-mouse CD122 (IL-2Rβ) https://www.biolegend.com/en-us/products/apc-anti-mouse-cd122-il-2rbeta-antibody-8908
PE-Cy7 anti-mouse MHC Class I (H-2Kb) https://www.thermofisher.com/antibody/product/MHC-Class-I-H-2Kb-Antibody-clone-AF6-88-5-5-3-Monoclonal/25-5958-82?utm_source=chatgpt.com
Alexa Fluor 700 anti-mouse CD44 https://www.thermofisher.com/antibody/product/CD44-Antibody-clone-IM7-Monoclonal/56-0441-82?utm_source=chatgpt.com
eFluor 450 anti-mouse CD279 (PD-1) https://www.thermofisher.com/antibody/product/PD-1-CD279-Antibody-clone-J43-Monoclonal/48-9985-82?utm_source=chatgpt.com

Flow blocking:
Human TruStain FcX https://www.biolegend.com/en-us/products/human-trustain-fcx-fc-receptor-blocking-solution-6462
TruStain FcX PLUS (anti-mouse CD16/32) Antibody https://www.biolegend.com/en-us/products/trustain-fcx-plus-anti-mouse-cd16-32-antibody-17085

Mouse Total SeqA antibodies:
CD4 https://www.biolegend.com/en-gb/products/totalseq-a0001-anti-mouse-cd4-antibody-16176
CD8a https://www.biolegend.com/en-gb/products/totalseq-a0002-anti-mouse-cd8a-antibody-15920
TCRb https://www.biolegend.com/en-gb/products/totalseq-a0120-anti-mouse-tcr-%CE%B2-chain-antibody-16424
CD44 https://www.biolegend.com/en-gb/products/totalseq-a0073-anti-mouse-human-cd44-antibody-15923
CD62L https://www.biolegend.com/en-gb/products/totalseq-a0112-anti-mouse-cd62l-antibody-16419
TCRgd https://www.biolegend.com/en-gb/products/totalseq-a0121-anti-mouse-tcr-gamma-sigma-antibody-16151
CD8b https://www.biolegend.com/en-gb/products/totalseq-a0230-anti-mouse-cd8b-ly-3-antibody-17124
GPR56 https://www.biolegend.com/en-gb/products/totalseq-a0912-anti-human-gpr56-antibody-18966
CD94 https://www.biolegend.com/en-gb/products/totalseq-a1009-anti-mouse-cd94-antibody-18686
Ly49D https://www.biolegend.com/en-gb/products/totalseq-a0841-anti-mouse-ly49d-antibody-17508
Ly49G https://www.biolegend.com/en-gb/products/totalseq-a0840-anti-mouse-ly49g-antibody-20699
Ly49H https://www.biolegend.com/en-gb/products/totalseq-a0839-anti-mouse-ly49h-antibody-18102
CD103 https://www.biolegend.com/en-gb/products/totalseq-a0201-anti-mouse-cd103-antibody-16456
CCR7 https://www.biolegend.com/en-gb/products/totalseq-a0377-anti-mouse-cd197-ccr7-antibody-17283
PD1 https://www.biolegend.com/en-gb/products/totalseq-a0004-anti-mouse-cd279-pd-1-antibody-15917
CD69 https://www.biolegend.com/en-gb/products/totalseq-a0197-anti-mouse-cd69-antibody-16422
CXCR4 https://www.biolegend.com/en-gb/products/totalseq-a0444-anti-mouse-cd184-cxcr4-antibody-17270
Mouse IgG1, κ isotype Ctrl Antibody https://www.biolegend.com/en-gb/products/totalseq-a0090-mouse-igg1-kappa-isotype-control-15774
Rat IgG2a, κ Isotype Ctrl Antibody https://www.biolegend.com/en-gb/products/totalseq-a0238-rat-igg2a-kappa-isotype-ctrl-antibody-16185
Rat IgG2b, κ Isotype Ctrl Antibody https://www.biolegend.com/en-gb/products/totalseq-a0095-rat-igg2b-kappa-isotype-ctrl-16228
Armenian Hamster IgG Isotype Ctrl Antibody https://www.biolegend.com/en-gb/products/totalseq-a0241-armenian-hamster-igg-isotype-ctrl-17278

Mouse Hashtag antibodies:
TotalSeq™-A0301 anti-mouse Hashtag 1 Antibody https://www.biolegend.com/en-gb/products/totalseq-a0301-anti-mouse-hashtag-1-antibody-16103
TotalSeq™-A0302 anti-mouse Hashtag 2 Antibody https://www.biolegend.com/en-gb/products/totalseq-a0302-anti-mouse-hashtag-2-antibody-16104
TotalSeq™-A0303 anti-mouse Hashtag 3 Antibody https://www.biolegend.com/en-gb/products/totalseq-a0303-anti-mouse-hashtag-3-antibody-16105
TotalSeq™-A0304 anti-mouse Hashtag 4 Antibody https://www.biolegend.com/en-gb/products/totalseq-a0304-anti-mouse-hashtag-4-antibody-16106

Human Total SeqC antibodies:
Hu.GPR15 https://www.biolegend.com/en-gb/products/totalseq-a1298-anti-human-gpr15-antibody-25399
TotalSeq™-C Human Universal Cocktail V1.0 https://www.biolegend.com/en-gb/products/totalseq-c-human-universal-cocktail-v1-0-19736

Human Hashtag antibodies:
HTO-1 https://www.biolegend.com/en-gb/products/totalseq-c0251-anti-human-hashtag-1-antibody-17162
HTO-2 https://www.biolegend.com/en-gb/products/totalseq-c0252-anti-human-hashtag-2-antibody-17163
HTO-3 https://www.biolegend.com/en-gb/products/totalseq-c0253-anti-human-hashtag-3-antibody-17164
HTO-4 https://www.biolegend.com/en-gb/products/totalseq-c0254-anti-human-hashtag-4-antibody-17165
HTO-5 https://www.biolegend.com/en-gb/products/totalseq-c0255-anti-human-hashtag-5-antibody-17166
HTO-6 https://www.biolegend.com/en-gb/products/totalseq-c0256-anti-human-hashtag-6-antibody-18373
HTO-7 https://www.biolegend.com/en-gb/products/totalseq-c0257-anti-human-hashtag-7-antibody-18374
HTO-8 https://www.biolegend.com/en-gb/products/totalseq-c0258-anti-human-hashtag-8-antibody-18375
HTO-9 https://www.biolegend.com/en-gb/products/totalseq-c0259-anti-human-hashtag-9-antibody-18376

Eukaryotic cell lines

Policy information about cell lines and Sex and Gender in Research

Cell line source(s)	HEK 293T cells (cat: CRL-3216), Jurkat T cells, clone E6-1 (cat: TIB-152), and HeLa 229 cells (cat: CCL-2.1) were purchased from the American Type Culture Collection (ATCC). Lenti-X 293T cells (cat: 632180) were purchased from Takara Bio. Platinum-E (Plat-E) cells (cat: RV-101) were purchased from Cell Biolabs. CHO-K1 cells stably transfected with Gα16 G protein were a gift from Eugene C. Butcher.
Authentication	Cell lines were not independently authenticated after purchase or receipt from the providing laboratories. Authentication was based on information provided by the vendors or source laboratories.
Mycoplasma contamination	The cell lines were not tested for mycoplasma contamination.
Commonly misidentified lines (See ICLAC register)	No commonly misidentified cell line used.

Animals and other research organisms

Policy information about studies involving animals; ARRIVE guidelines recommended for reporting animal research, and Sex and Gender in Research

Laboratory animals	Gpr15−/− mice were a kind gift from Eugene C. Butcher and were generated by backcrossing B6;129P2-Gpr15tm1.1Litt/J (The Jackson Laboratory, Strain #: 008769) to C57BL/6 wild-type mice over 12 generations. Rag2−/−Il2rg−/− mice (Line #: 4111), CD45.1 C57BL/6 mice (Line #: 4007), and CD45.2 C57BL/6 mice (Line #: B6) were purchased from Taconic. Gpr15fl/fl (Strain #: 028324), CD8-cre (Strain #: 008766), and Foxp3-cre (Strain #: 016959) mice were purchased from The Jackson Laboratory. Mice were housed in a specific pathogen-free facility under a 12-h light:dark cycle at 22±2°C with 70% humidity. After weaning, mice were separated by sex and co-housed by genotype. Sex-matched male and female littermates aged 8-12 weeks were used for all experiments.
Wild animals	This study did not involve wild animals.
Reporting on sex	We used both sexes in animal studies.
Field-collected samples	This study did not involve field-collected samples.
Ethics oversight	All animal protocols (ASP#: LISB-7E, EIB-104, EIB-105) were approved by the Animal Care and Use Committee of the National Institute of Allergy and Infectious Diseases (NIAID) or the National Cancer Institute (NCI) in accordance with NIH and Department of Health and Human Services (HHS) guidelines.

Note that full information on the approval of the study protocol must also be provided in the manuscript.

Plants

Seed stocks	N/A
Novel plant genotypes	N/A
Authentication	N/A

Flow Cytometry

Plots

Confirm that:

- [x] The axis labels state the marker and fluorochrome used (e.g. CD4-FITC).
- [x] The axis scales are clearly visible. Include numbers along axes only for bottom left plot of group (a 'group' is an analysis of identical markers).
- [x] All plots are contour plots with outliers or pseudocolor plots.
- [x] A numerical value for number of cells or percentage (with statistics) is provided.

Methodology

Sample preparation

Human peripheral blood mononuclear cells (PBMCs) were isolated using Ficoll-Paque PLUS (Cytiva, cat: GE17-1440-02) by density-gradient centrifugation and washed twice with PBS.

For isolation of human intestinal lymphocytes, frozen biopsies were thawed at 37 °C, washed in cRPMI, and minced into small pieces. Intraepithelial lymphocytes (IELs) were isolated by rotating tissues in HBSS containing 10% FCS, 5 mM EDTA, and 5 mM dithiothreitol (Sigma, cat: 10197777001) at 37 °C for 40 min, then filtered through 100-µm cell strainers. Remaining tissue was digested in RPMI containing 1 mg/mL Collagenase IV (Sigma, C5138) and 10 U/mL DNase I (Roche, cat: 04716728001) at 37 °C for 1 h to isolate lamina propria (LP) cells. Cells were filtered and centrifuged on a 40% Percoll gradient at 1,800 rpm, 4 °C for 10 min.

For mouse lymphocytes preparation, spleen, mesenteric lymph nodes (MLN), thymus, small intestine, and large intestine were collected from euthanized mice. Spleen, MLN, and thymus were mechanically dissociated and filtered through 70-µm cell strainers in RPMI to generate single-cell suspensions. For intestinal cell isolation, visible fat and luminal contents were removed, intestines were opened longitudinally, and Peyer's patches were removed from the small intestine. Tissues were cut into 0.5–1 cm pieces and incubated at 37 °C for 30 min in HBSS containing 5 mM EDTA and 1 mM dithiothreitol to isolate intestinal epithelial cells (IECs) and IELs. Cell suspensions were filtered through 100-µm mesh filters, washed with RPMI containing 3% FBS, and centrifuged at 800 × g for 10 min. IECs and IELs were separated by centrifugation in 40% Percoll (GE Healthcare, cat: GE17-0891-01) at 1,800 rpm for 10 min. Remaining intestinal tissue was minced and digested at 37 °C for 40 min in RPMI containing 0.1 mg/mL Liberase (Roche, cat: 05401020001) and 10 U/mL DNase I. Digested tissue was filtered through a 70-µm cell strainer and centrifuged at 500 × g for 5 min. Intestinal LP lymphocytes were isolated by centrifugation in 40% Percoll at 1,800 rpm for 10 min.

For flow cytometry analysis of CD8+ T cell migration to mouse intestinal organoids, 2 days after co-culture, medium containing free CD8+ T cells was collected. Organoids were released from Matrigel using Cell Recovery Solution on ice for 30 min, and supernatants containing free CD8+ T cells were combined with the collected medium. Free CD8+ cells were pelleted by centrifugation at 500 × g for 5 min. To isolate organoid-incorporated CD8+ T cells, organoid pellets were incubated in 5 mM EDTA/PBS on ice for 10 min, mechanically dissociated by vigorous pipetting, and centrifuged at 500 × g for 5 min. Free and organoid-incorporated CD8+ T cells were subsequently analyzed by flow cytometry.

Instrument

Flow cytometry data were acquired on FACSymphony A3 or LSRFortessa (BD Biosciences)

Software

We used the software FACSDiva (v9.1, v9.0, BD Biosciences) to collect the data and the software FlowJo (Treestar Inc., v10.9.0) to analyze the data.

Cell population abundance

Cell numbers for flow cytometry were titrated, based on past experience.

Gating strategy

For all experiments, we first selected lymphocytes based on FSCxSSC; then, we selected the singlets based on FSC-H x FSC-W, then SSC-H x SSC-W; then we selected viable cells based on FSC x Live-Dead gating.

☒ Tick this box to confirm that a figure exemplifying the gating strategy is provided in the Supplementary Information.
