# Supplementary material for: GPR15-guided CD8+ T regulatory cells control intestinal inflammation
Source: Nature. 2026 Jun 8;656(8129):1003–12. doi: 10.1038/s41586-026-10749-4 (PMC13518229; doi:10.1038/s41586-026-10749-4)
Supplement: Supplementary file 3 [file 41586_2026_10749_MOESM3_ESM.pdf]

# Supplementary Figures

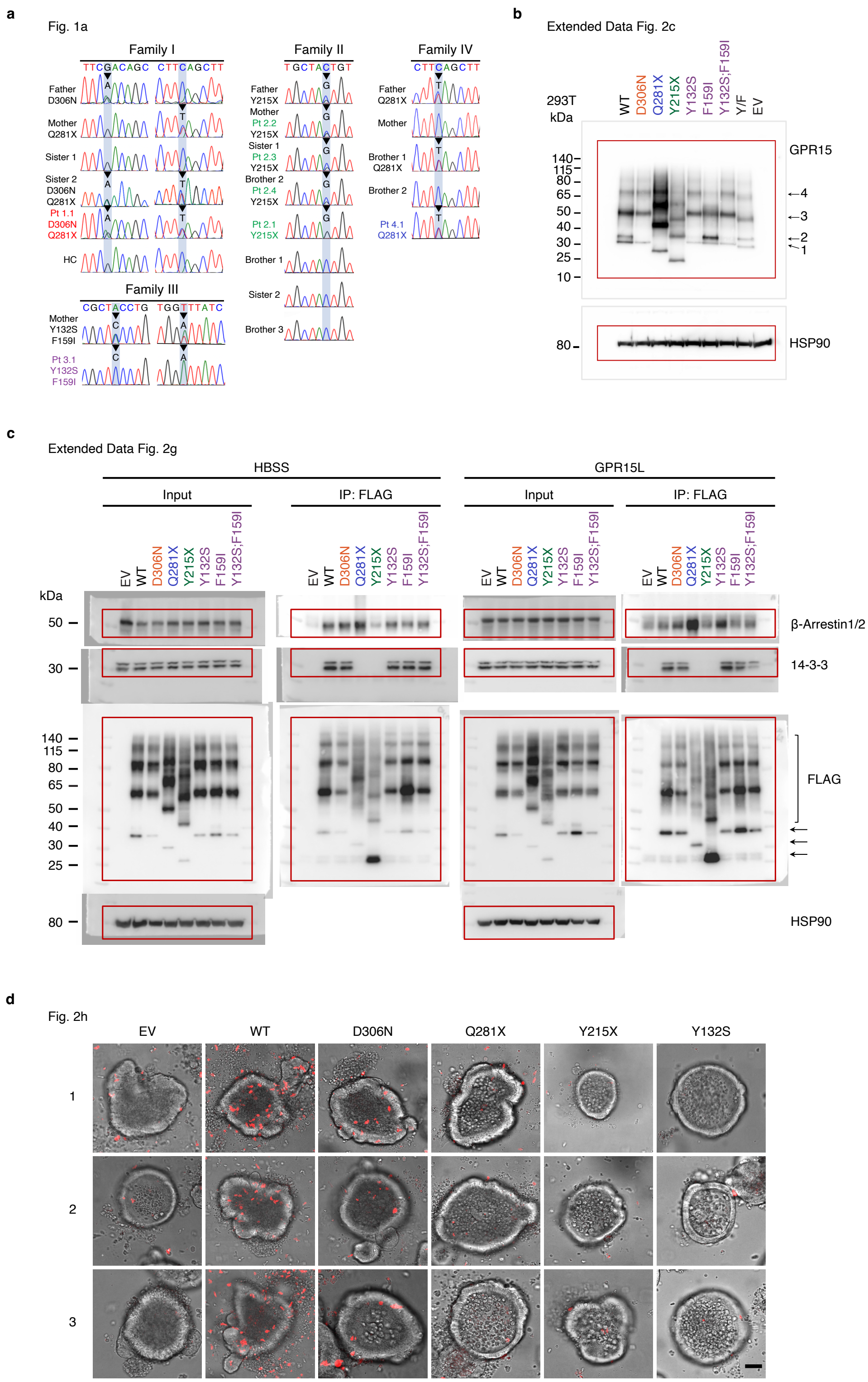

**Supplementary Figure 1 | Sanger sequencing confirmation of GPR15 mutations and source data for immunoblots and microscopy images. a**, Sanger sequencing confirmation of GPR15 mutations identified in four families. Arrowheads indicate the mutant nucleotides. **b, c**, Uncropped immunoblots corresponding to the indicated figure panels. Red boxes represent the cropped regions. Molecular weight markers (kDa) and the antibodies used for immunoblotting are indicated. **d**, Representative confocal images of *Gpr15*$^{-/-}$ CD8$^{+}$ T cells transduced with mCherry-tagged WT, mutants (equivalent to patient variants), or empty vector (EV), co-cultured with mouse intestinal organoids for 48 h (Fig. 2h). Red, mCherry$^{+}$ T cells. Scale bars, 30 μm.

a

Fig. 1d

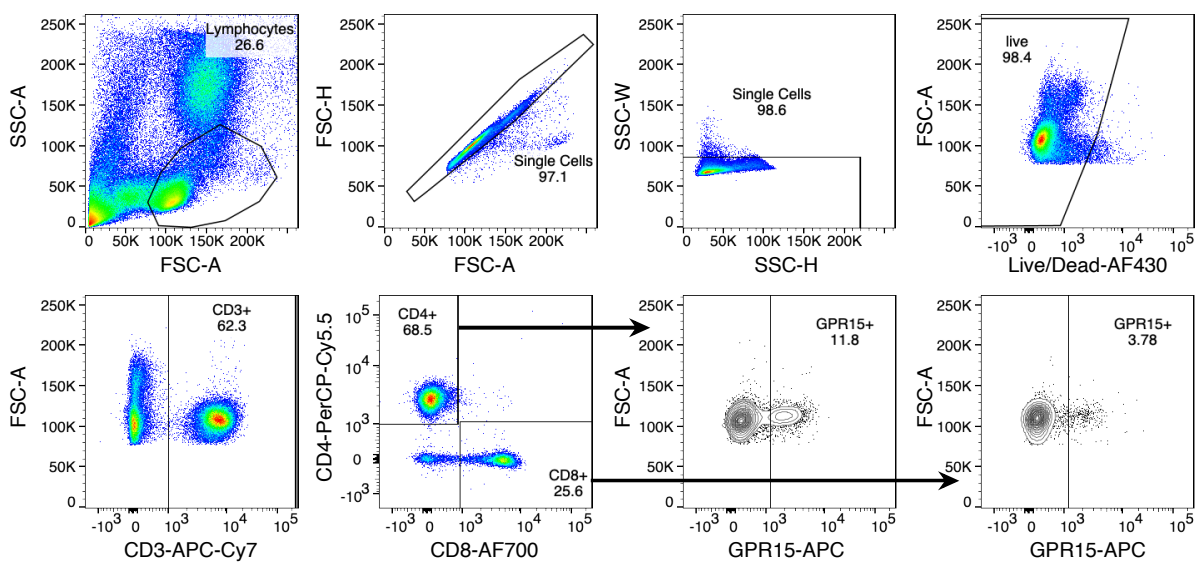


b

Fig. 1e

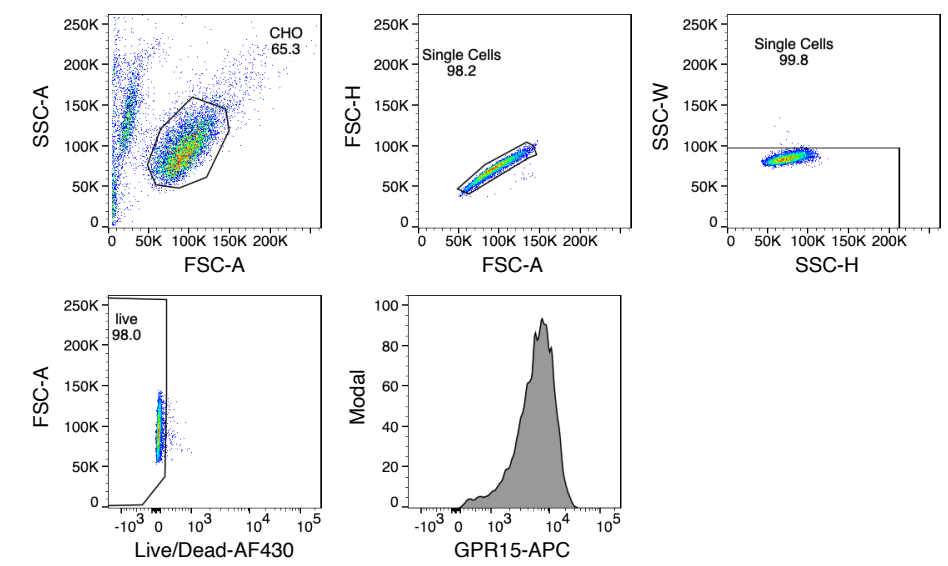


c

Fig. 1g

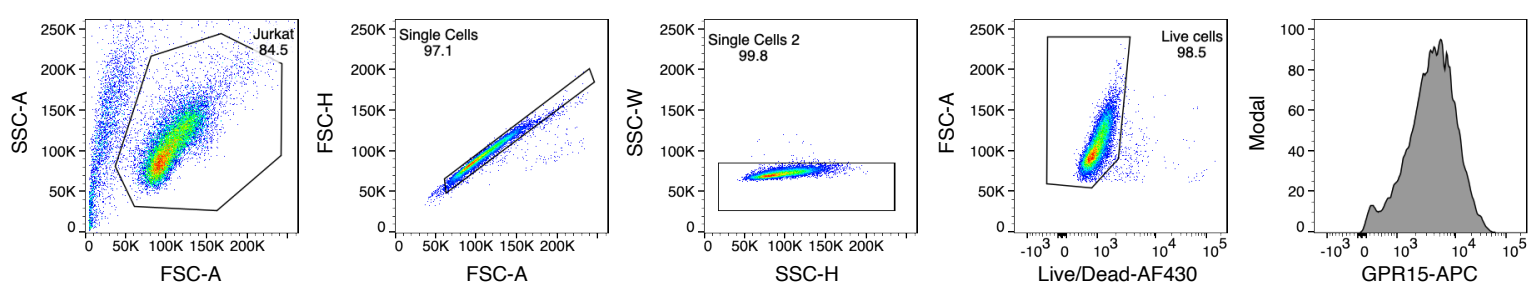


d

Fig. 1j

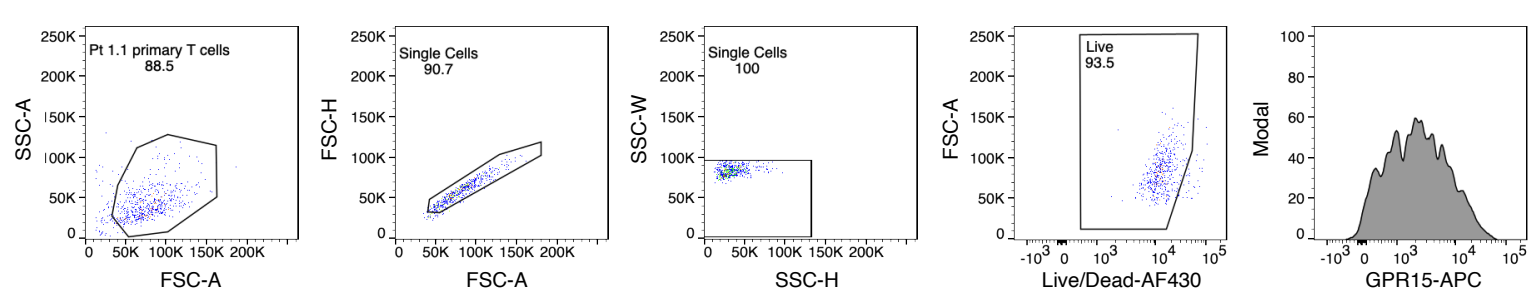


e

Extended Data Fig. 1e

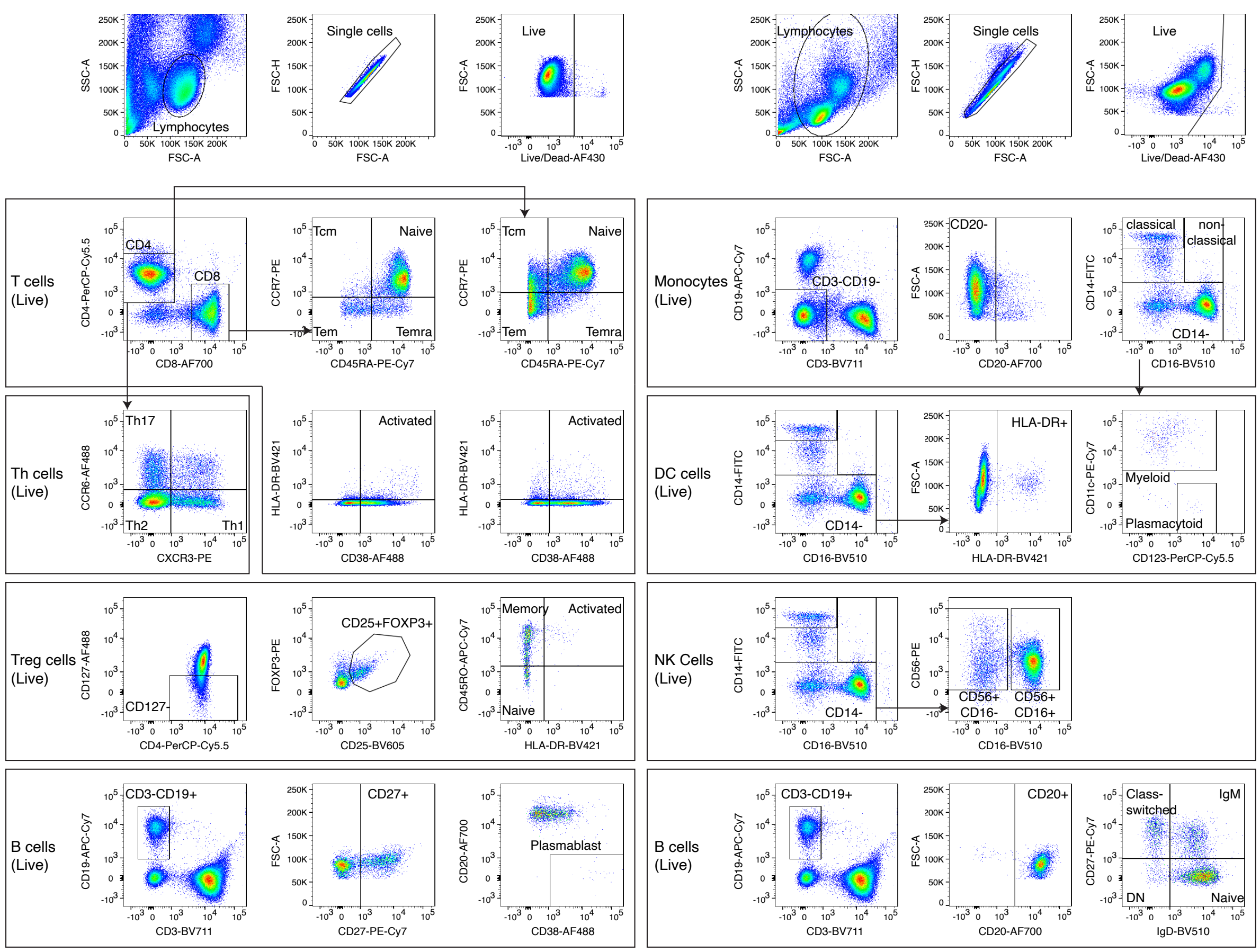

**Supplementary Figure 2 | Flow cytometry gating strategies for GPR15 expression and human immune cell immunophenotyping. a**, Gating strategy used to determine the frequency of $GPR15^+$ cells among human peripheral $CD4^+$ and $CD8^+$ T cells (Fig. 1d). **b-d**, Representative gating strategies used to quantify surface GPR15 expression in CHO cells (Fig. 1e), Jurkat T cells (Fig. 1g), and primary patient T cells (Fig. 1j) transduced with wild-type (WT) GPR15. The same gating strategy was applied to GPR15 mutants and empty vector (EV) controls. **e**, Gating strategies used for immunophenotyping of peripheral blood from healthy controls (HCs), patients, and family members from Families I, III, and IV (Extended Data Fig. 1e).

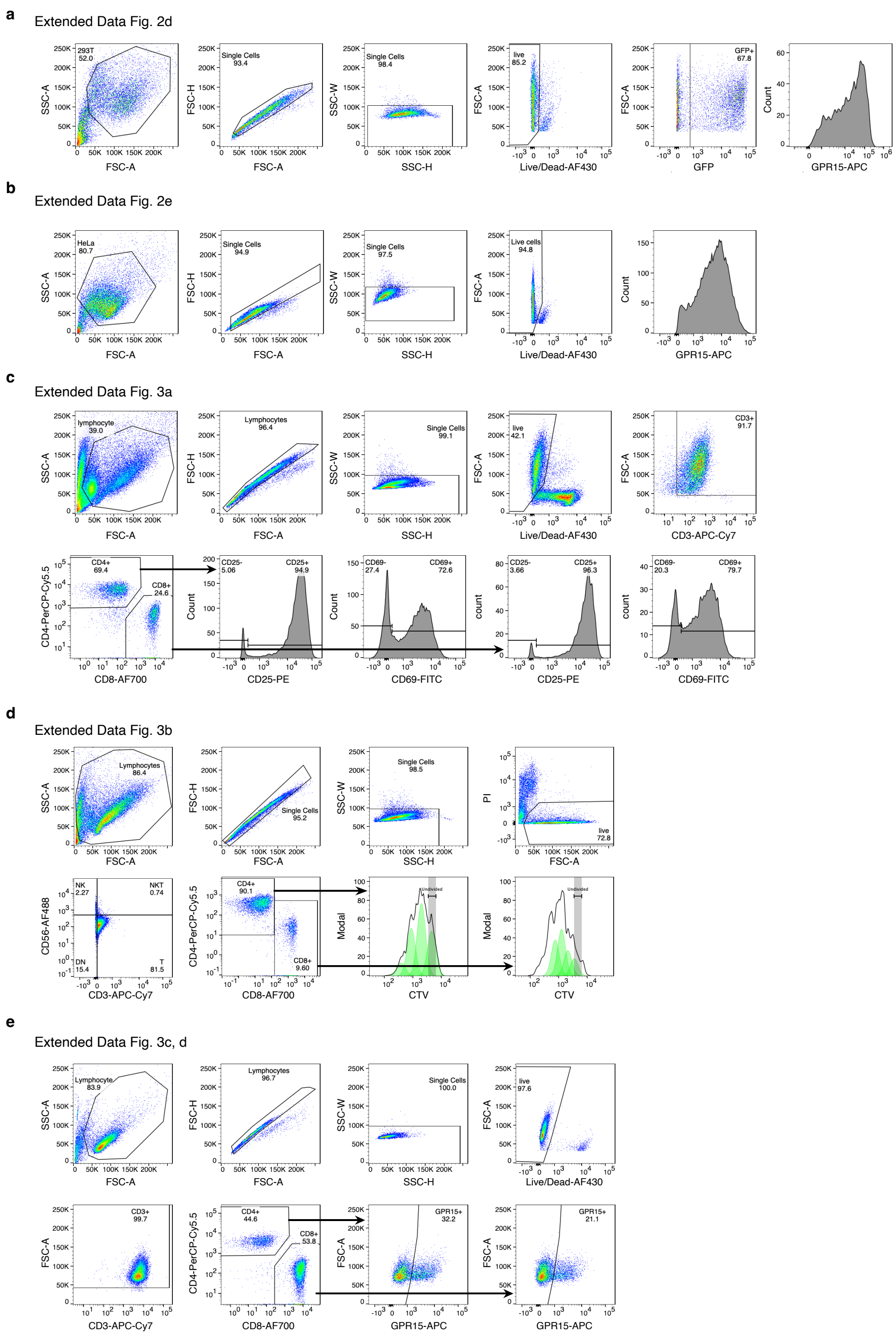


**Supplementary Figure 3 | Flow cytometry gating strategies for GPR15 expression and T cell functional assays.** **a, b**, Representative gating strategies used to quantify surface GPR15 expression in HEK 293T (Extended Data Fig. 2d) and HeLa cells (Extended Data Fig. 2e) transduced with wild-type (WT) GPR15. The same gating strategy was applied to GPR15 mutants and empty vector (EV) controls. **c-e**, Gating strategies for analysis of activated human primary T cells following anti-CD3/CD28 stimulation. **c**, Quantification of $CD25^+$ and $CD69^+$ cells among $CD4^+$ and $CD8^+$ T cells (Extended Data Fig. 3a). **d**, Assessment of proliferation by CellTrace Violet (CTV) dilution in $CD4^+$ and $CD8^+$ T cells (Extended Data Fig. 3b). **e**, Determination of $GPR15^+$ cell frequencies among $CD4^+$ and $CD8^+$ T cells (Extended Data Fig. 3c, d).

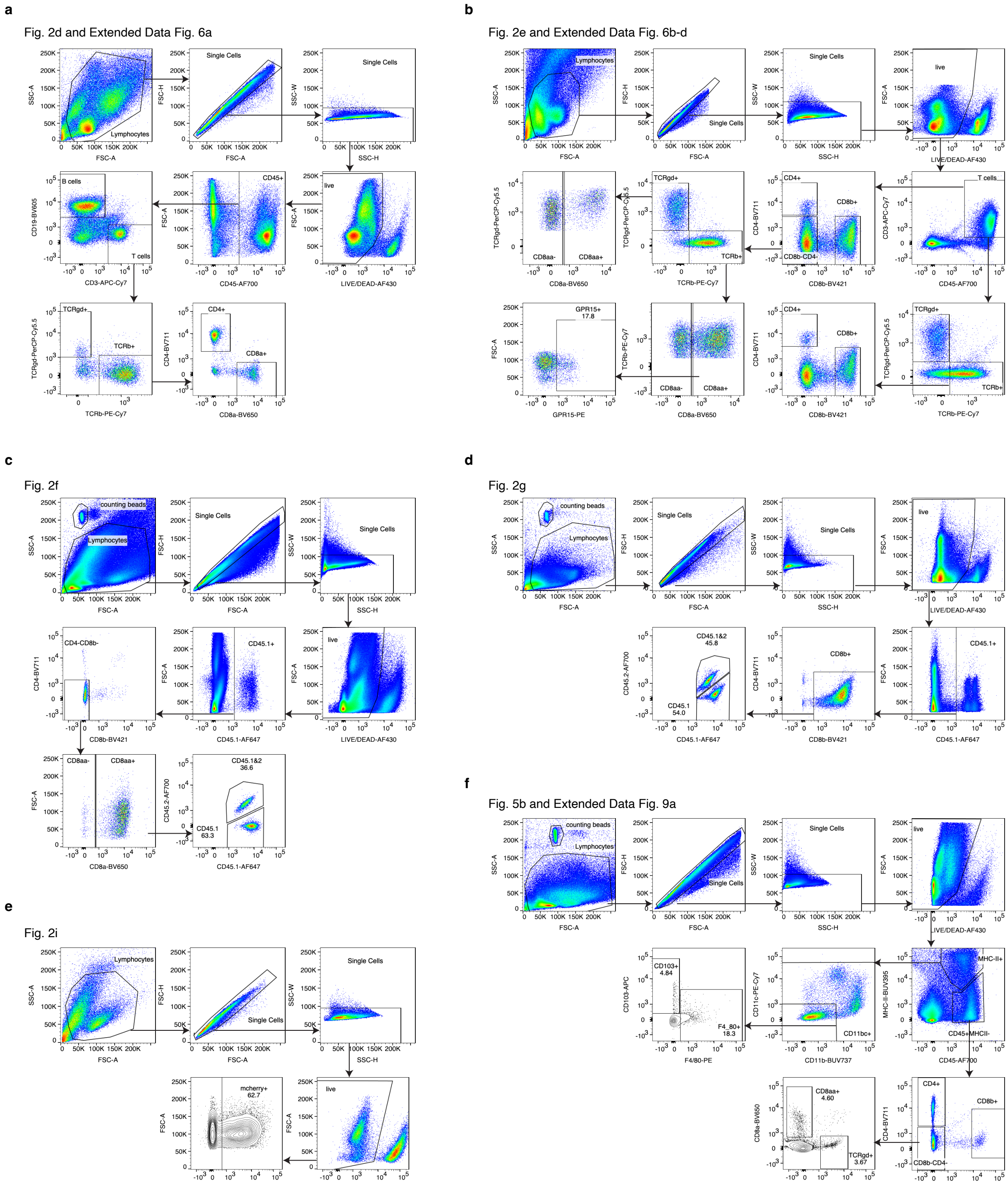


**Supplementary Figure 4 | Flow cytometry gating strategies for murine intestinal immune cell analyses. a**, Gating strategy for quantification of mouse intestinal lymphocytes in the large intestinal epithelium (LI-IEL) and lamina propria (LI-LP) (Fig. 2d and Extended Data Fig. 6a). **b**, Gating strategy for analysis of mouse intestinal intraepithelial lymphocyte (IEL) subsets (Fig. 2e and Extended Data Fig. 6b-d). **c, d**, Representative gating strategies used to determine tester-to-competitor ratios of TCRαβ$^{+}$CD8αα$^{+}$ cells from the small intestinal IEL (**c**) and TCRαβ$^{+}$CD8αβ$^{+}$ cells from splenocytes (**d**) in recipient mice following adoptive transfer (Fig. 2f, g). The same gating strategies shown in a-d were applied to all analyzed tissues. **e**, Gating strategy for analysis of mCherry$^{+}$ CD8$^{+}$ T cells migrated into mouse intestinal organoids (Fig. 2i). **f**, Gating strategy for quantification of IELs, F4/80$^{+}$ macrophages, and CD103$^{+}$ dendritic cells (DCs) in the mouse LI-LP 3 days after DSS treatment (Fig. 5b and Extended Data Fig. 9a).

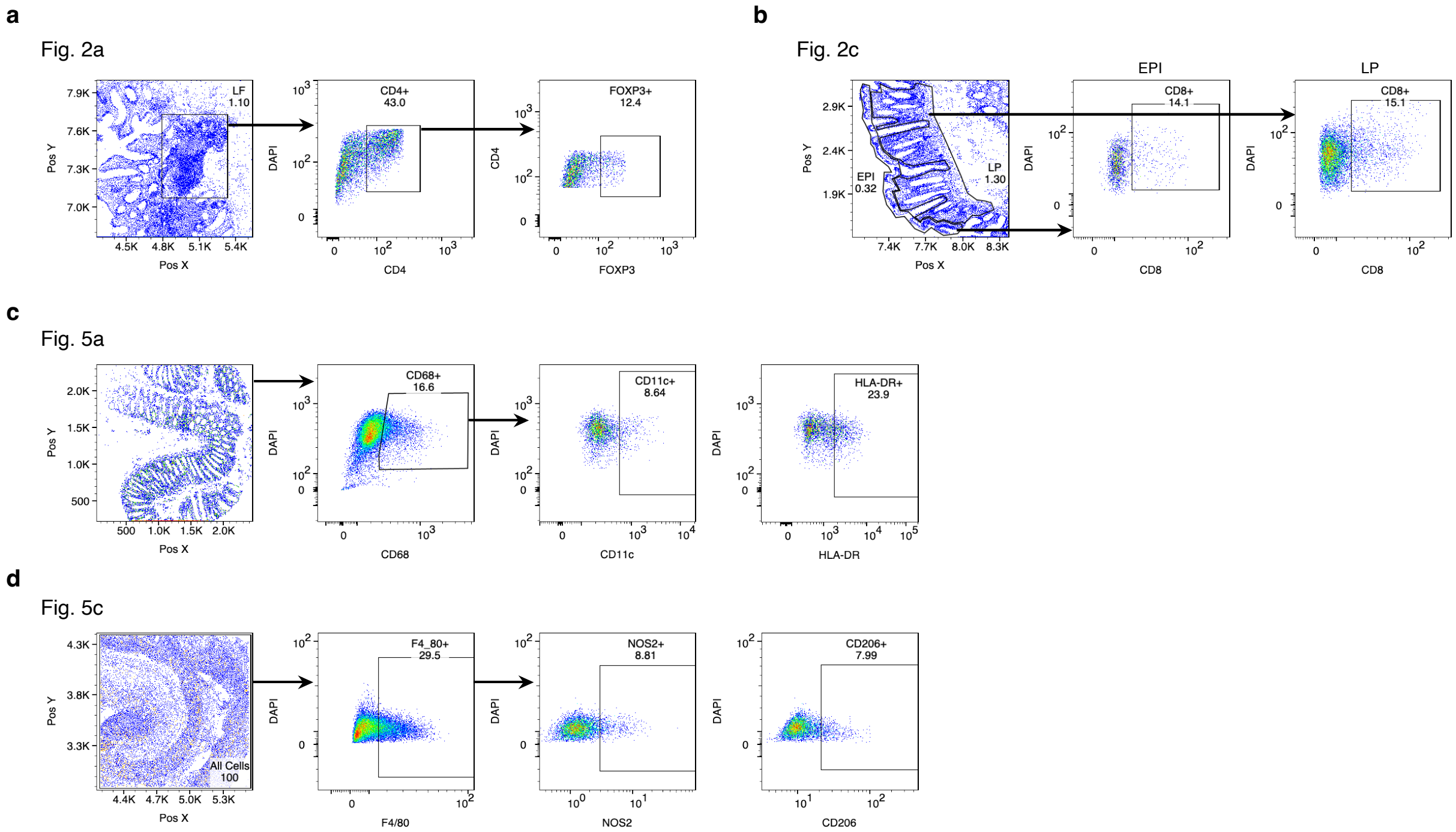


**Supplementary Figure 5 | Gating strategies for multiplex immunofluorescence histocytometry analyses. a, b**, Representative gating strategies used to quantify FOXP3$^+$CD4$^+$ T cells (**a**) and CD8$^+$ T cells (**b**) within colonic lymphoid follicles (LF), epithelial (EPI), and lamina propria (LP) compartments in human intestinal biopsy sections (Fig. 2a, c). **c**, Gating strategy to quantify the percentage of CD11c$^+$ and HLA-DR$^+$ cells among macrophages (CD68$^+$) in human colonic biopsy sections (Fig. 5a). **d**, Gating strategy used to quantify the percentage of NOS2$^+$ and CD206$^+$ cells among macrophages (F4/80$^+$) in mouse colonic sections 5 days after DSS treatment (Fig. 5c).
